# Graphical Modeling of Gene Expression in Monocytes Suggests Molecular Mechanisms Explaining Increased Atherosclerosis in Smokers

**DOI:** 10.1371/journal.pone.0050888

**Published:** 2013-01-23

**Authors:** Ricardo A. Verdugo, Tanja Zeller, Maxime Rotival, Philipp S. Wild, Thomas Münzel, Karl J. Lackner, Henri Weidmann, Ewa Ninio, David-Alexandre Trégouët, François Cambien, Stefan Blankenberg, Laurence Tiret

**Affiliations:** 1 INSERM UMR_S 937, Pierre and Marie Curie University, Paris, France; 2 University Heart Center Hamburg, Department of General and Interventional Cardiology, Hamburg, Germany; 3 Department of Medicine II, University Medical Center Mainz, Mainz, Germany; 4 Clinical Epidemiology, Center for Thrombosis and Haemostasis, University Medical Center Mainz, Mainz, Germany; 5 Institute for Clinical Chemistry and Laboratory Medicine, University Medical Center Mainz, Mainz, Germany; Queen's University Belfast, United Kingdom

## Abstract

Smoking is a risk factor for atherosclerosis with reported widespread effects on gene expression in circulating blood cells. We hypothesized that a molecular signature mediating the relation between smoking and atherosclerosis may be found in the transcriptome of circulating monocytes. Genome-wide expression profiles and counts of atherosclerotic plaques in carotid arteries were collected in 248 smokers and 688 non-smokers from the general population. Patterns of co-expressed genes were identified by Independent Component Analysis (ICA) and network structure of the pattern-specific gene modules was inferred by the PC-algorithm. A likelihood-based causality test was implemented to select patterns that fit models containing a path “smoking→gene expression→plaques”. Robustness of the causal inference was assessed by bootstrapping. At a FDR ≤0.10, 3,368 genes were associated to smoking or plaques, of which 93% were associated to smoking only. *SASH1* showed the strongest association to smoking and *PPARG* the strongest association to plaques. Twenty-nine gene patterns were identified by ICA. Modules containing *SASH1* and *PPARG* did not show evidence for the “smoking→gene expression→plaques” causality model. Conversely, three modules had good support for causal effects and exhibited a network topology consistent with gene expression mediating the relation between smoking and plaques. The network with the strongest support for causal effects was connected to plaques through *SLC39A8*, a gene with known association to HDL-cholesterol and cellular uptake of cadmium from tobacco, while smoking was directly connected to *GAS6*, a gene reported to have anti-inflammatory effects in atherosclerosis and to be up-regulated in the placenta of women smoking during pregnancy. Our analysis of the transcriptome of monocytes recovered genes relevant for association to smoking and atherosclerosis, and connected genes that before, were only studied in separate contexts. Inspection of correlation structure revealed candidates that would be missed by expression-phenotype association analysis alone.

## Introduction

Smoking is a major risk factor for atherosclerosis and its complications, particularly coronary artery disease (CAD) and peripheral arterial disease [1]–[3]. Pathophysiological mechanisms by which smoking promotes atherogenesis are relatively well known, in particular through alterations of lipid metabolism [4], [5] and endothelial function [6]. However, the molecular mechanisms by which smoking exerts its adverse effects at the cellular level are less documented. The advent of transcriptomic studies allowing investigation of all the genes expressed in a given type of cell has opened a new window for exploring in a global way the biological mechanisms underlying pathophysiological conditions. Using such transcriptomic approach, widespread perturbation of gene expression by smoking has been recently shown in whole blood [7], circulating lymphocytes [8], and monocytes [9] of humans.

Increasing evidence supports the hypothesis that oxidative stress and activation of the immune system provide a pathophysiological link between cigarette smoking and CAD [10], [11]. Monocytes are key cells of the immune system involved in the inflammatory response to external agents. We hypothesized that the effect of smoking on atherosclerosis might be reflected by perturbation of gene expression in circulating monocytes and that it might be possible to identify gene networks causally involved in the relationship linking smoking to atherosclerosis.

Questions about causal effects in observational studies can be addressed by statistical methods that can translate statements about correlations and conditional independencies into structural equations [12] or Bayesian Networks [13]. Implementation of both techniques, however, can be difficult when the number of variables is large and inference of the “true” network that generated the data may not be feasible, even with large sample sizes. In addition, current implementations of Bayesian Network inference restrict to systems of Gaussian only, binomial (or multinomial) only or hybrids where binomials can only precede but not be caused by Gaussian variables [14]. A third class of methods based on information theory has been developed for the problem of identification of large networks of direct gene interactions, which does not rely on the correct specification of distribution functions, but where such interactions do not have a causal interpretation (e.g. ARACNE [15], SA-CLR [16], and Parmigene [17], among others). For the problem under study here, an approach that is not limited in the types of variables that can be modeled, i.e. binary, continuous, and counts, or in how they may be associated, that allows inferences about causal relations, and that can deal with large number of variables was needed.

The objective of the present study was to identify groups of genes that may help explain the causal effect of smoking on extent of atherosclerosis. For this purpose, genomewide gene expression in monocytes was modeled as a molecular phenotype potentially linking smoking to carotid atherosclerosis. Data were obtained from the Gutenberg Health Study (GHS), a community-based project primarily aimed at improving cardiovascular risk prediction [9]. We devised a stepwise approach to: 1. Identify patterns of expression associated to smoking and/or atherosclerosis using Independent Component Analysis (ICA); 2. Select expression patterns showing relatively high support for a causal role in the mediation between smoking and atherosclerosis using graphical modeling and Bayesian Network (BN) inference; 3. For patterns compatible with a potential causal role, infer the network skeleton connecting smoking, genes and atherosclerosis.

Our approach identified three gene networks that were compatible with a causal effect of gene expression mediating the relation between smoking and atherosclerosis where a few genes are candidates for mediating the perturbing effect of smoking in these networks. By performing causal inference on independent patterns of expression instead of the expression of a single gene, we not only dramatically reduced the space of models to be tested, but also applied BN inference in a space that is less prone to the effects of hidden variables. We restricted to detecting classes of best fitting models rather than a single causal model and present arguments for why some causal models may be favored in this study. We also provide cautionary statements to avoid misinterpretation of the reported causal models.

## Results

### Smoking and extent of atherosclerosis in the GHS cohort

Association between smoking habits and atherosclerosis was investigated in a cohort of subjects of both sexes aged 35 to 74 years who participated in the GHS [9]. Study participants were classified into current (≥1 cigarette/day) smokers (*n* = 248) and nonsmokers (*n* = 688). Occasional smokers (*n* = 42) and ex-smokers (*n* = 547) were excluded from the study (Methods). Characteristics of the study population are given in Table 1.

**Table 1:** Characteristics of the Gutenberg Health Study population.

Characteristics	Variable name	Females	Males	p-value†
Number of individuals	N	522 (55.77%)	414 (44.23%)	
Age (years)	age	54.6 (0.49)	54.2 (0.53)	0.5825
Body mass index (kg/m^2^)	bmi	26.2 (0.22)	27.3 (0.2)	0.0003
Triglycerides (mg/dl) (log)	TRIGLY	4.63 (0.019)	4.78 (0.025)	4.6E-06
HDL cholesterol (mg/dl)	HDL_CHOL	63.8 (0.69)	50.6 (0.6)	2.3E-40
LDL cholesterol (mg/dl)	LDL_CHOL	144 (1.7)	142 (1.8)	0.6472
Systolic blood pressure (mmHg)	sbp	129 (0.83)	134 (0.77)	2.2E-06
Diabetes (type I or II)	diabetes	30 (5.7%)	30 (7.2%)	0.3538
Current smoker	smoking	115 (22%)	133 (32.1%)	0.0005
C-reactive protein (mg/l) (log)	CRP	0.932 (0.033)	0.832 (0.038)	0.0455
Homocysteine (mmol/l) (log)	HCY	2.25 (0.013)	2.44 (0.015)	1.3E-21
Myeloperoxidase (pmol/l) (log)	MPO	5.76 (0.015)	5.7 (0.02)	0.0157

† p-values calculated from a χ^2^ test for smoking and diabetes (number of subjects), and from an F test for all others. Standard errors or percents of individuals are in parenthesis.

Atherosclerosis extent was defined as the total number of atherosclerotic plaques measured in the two carotid arteries by ultrasound echography (Methods). Carotid intima-media thickness and carotid plaque are well-recognized markers of subclinical atherosclerosis [18] which are influenced by smoking [19]. The number of plaques observed per person ranged from 0 to 11, with a skewed distribution and an average of 0.72 plaques (variance 1.95). The prevalence of atherosclerosis, defined as the presence of at least one plaque, was 31.2% in this middle-aged population. The prevalence and the extent of atherosclerosis were higher in men than in women and in smokers than in non-smokers (Table 2).

**Table 2:** Mean number of carotid atherosclerotic plaques by sex and smoking status.

Sex	Smoking	N	Plaques Average†	Plaques>0	
				n¥	%
Females	Nonsmokers	407	0.51	107	26.3
	Smokers	115	0.56	30	26.1
	All	522	0.52	137	26.2
Males	Nonsmokers	281	0.74	93	33.1
	Smokers	133	1.42	62	46.6
	All	414	0.96	155	37.4
Both	Nonsmokers	688	0.61	200	29.1
	Smokers	248	1.02	92	37.1
	**All**	**936**	**0.72**	**292**	**31.2**

† average number of plaques per individual;

¥ number of individuals with at least 1 plaque.

In the following, the phenotype considered was the number of atherosclerotic plaques, referred to as “plaques”. Because the distribution showed overdispersion, a negative binomial distribution was used for modeling plaques as a function of covariables (see Text S1). The major determinants of plaques were age, sex and smoking, which all together explained 30% of the variability of plaques. The effect of smoking on plaques was stronger in men than in women although the significance of the interaction test was borderline (p = 0.027). Additional cardiovascular factors tested for association resulted in a modest increase of the explained variance of plaques (from 30% to 34%) (Table S1).

### Gene expression in monocytes is associated to smoking and plaques

**Figure 1:**
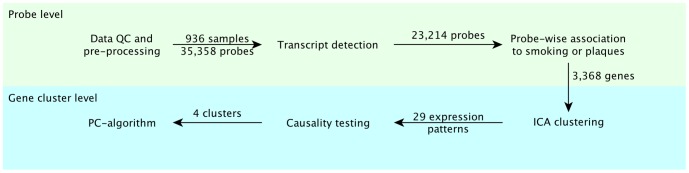
Analysis workflow. Microarray expression data were analyzed at two levels, a probe level (top) and a gene cluster level (bottom). Of the 35,358 probes with a “good” or “perfect” score according to ReMOAT, 23,214 were detected in monocytes of 936 subjects. Of these, 3,960 probes were associated to smoking or atherosclerotic plaques at an FDR ≤0.1, corresponding to 3,368 unique genes that were further clustered in 29 expression patterns by ICA. Causality testing revealed 4 patterns that were compatible with expression mediating the relationship between smoking and plaques. The skeleton of the network connecting genes, smoking, risk factors and plaques was then inferred using the PC algorithm.

The analysis workflow of expression data is outlined in Figure 1. Expression of 18,364 genes was detected in total RNA from circulating monocytes by 23,214 probes in the *Illumina* Human HT-12 BeadChip (Methods). Association of probe expression level with smoking or plaques (log-transformed) was assessed by linear model adjusted for age and sex, as well as for the 6 first singular value decomposition (SVD) components of the expression matrix taken as surrogate variables for technical sources of variability (Methods and Text S1).

In a first step, we identified genes whose expression was associated to either smoking or plaques by univariate analysis. At FDR ≤0.1, we found 3,774 probes (3,062 distinct genes) associated to smoking. The list of smoking-associated genes was enriched in three “biological processes” in the Gene Ontology (GO) database: platelet activation, interferon-gamma-mediated signaling pathway, and Toll signaling pathway (Table 3). Association between plaques and gene expression was much less prevalent than with smoking, with only 258 probes (236 distinct genes) associated to plaques at FDR ≤0.1. No GO terms were significantly enriched for genes associated to plaques.

**Table 3:** Gene Ontology categories enriched for smoking-associated gene expressions.

Term	Genes in GO class	Smoking-associated genes	p-value	Bonferroni corrected*
***GO: Biological Process (BP)***				
platelet activation	224	74	5.50E-07	0.0055
interferon-γ -mediated signaling pathway	65	29	4.50E-06	0.0451
Toll signaling pathway	71	30	4.60E-06	0.0461
***GO: Cellular Component (CC)***				
cytosol	1889	469	4.30E-15	6.02E-12
soluble fraction	315	91	2.50E-06	0.0035
focal adhesion	100	38	2.80E-06	0.0039
melanosome	87	33	3.40E-06	0.0048
cytoplasm	7509	1634	3.70E-06	0.0052
MHC class II protein complex	12	9	3.00E-05	0.0420
***GO: Molecular Function (MF)***				
protein binding	6620	1433	1.70E-12	4.38E-09

* Bonferroni correction on the number of GO terms represented in the reference set: 5010 (BP), 700 (CC), 1289 (MF).

Table 4 shows the 10 genes with strongest association to smoking or plaques, respectively. The whole list of associated genes to either phenotype is given in Table S2. There were 2 members common to both top 10 gene lists, *SASH1* and *PTGDS*. In addition to *SASH1* and *PTGDS*, 4 genes of the top 10 list for smoking were ranked among the top 100 genes for plaques: *FUCA1, LOC157627, MMP25* and *PID1* (Table S2). Smoking was associated to a much larger variability of gene expression (from 35% to 15% for the top 10 smoking-related genes) than plaques (from 5% to 2% for the top 10 plaques-related genes). The correlation of 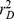 values for smoking and plaques across all genes was 0.4, indicating a strong association between smoking and plaques effects on gene expression as a result of their confounding effects.

**Table 4:** Top 10 genes associated to smoking or plaques.

ProbeID	Symbol	beta	r^2^_D_	p-value
**Top 10 genes associated to smoking**				
ILMN_2185984	SASH1	0.58	0.35	1.4E-88
ILMN_1660031	P2RY6	0.20	0.26	2.0E-61
ILMN_1717207	MMP25	0.59	0.22	7.5E-53
ILMN_1752728	FUCA1	0.33	0.19	7.2E-45
ILMN_1671891	PID1	0.37	0.19	4.3E-44
ILMN_1664464	PTGDS	−0.68	0.19	6.7E-44
ILMN_1656300	GFRA2	0.42	0.17	1.2E-38
ILMN_1708303	CYP4F22	−0.42	0.16	1.3E-37
ILMN_1655987	STAB1	0.29	0.16	3.4E-37
ILMN_1818677	LOC157627	0.12	0.15	1.8E-34
**Top 10 genes associated to plaques**				
ILMN_1800225	PPARG	0.09	0.05	2.26E-11
ILMN_2185984	SASH1	0.16	0.04	1.77E-09
ILMN_2103107	ADAMDEC1	0.07	0.03	1.70E-08
ILMN_1664464	PTGDS	−0.24	0.03	4.61E-08
ILMN_1706304	EIF2C4	0.05	0.03	1.31E-07
ILMN_2352633	ARHGAP24	0.05	0.03	2.40E-07
ILMN_1794914	UBTD1	0.05	0.03	2.93E-07
ILMN_1802808	SLC25A37	0.12	0.03	3.17E-07
ILMN_1752478	DHRS3	−0.12	0.03	5.35E-07
ILMN_1687592	WWC3	0.06	0.02	1.46E-06

Association of probe level was tested separately with smoking and ln(plaques+1) by linear model adjusted for age, sex and the 6 first SVD components. The beta regression coefficient is shown. Probes were ranked according to decreasing r^2^_D_ for smoking (plaques, respectively). In case of several probes by gene (e.g. *SASH1* and *PPARG*), the probe with the highest r^2^_D_ is shown.

### Comparison of smoking-associated gene expressions in monocytes and lymphocytes

We investigated the robustness of the association between gene expression and smoking by comparing the list of smoking-associated genes in monocytes to a list of 323 genes that have been found associated to smoking in lymphocytes [8]. The two studies used different microarray platforms and the 13,707 genes common to both studies were taken as the reference set. Of the 323 genes associated to smoking in lymphocytes, 268 were detected in monocytes of GHS of which 151 were associated to smoking (56.4%). This represented a 2.5-fold enrichment versus the reference (p = 4.6×10^−34^). Using a more stringent FDR threshold of 0.05 rather than 0.10 as in [8] did not significantly affect the results (2,477 unique genes associated to smoking in GHS; 2.6-fold enrichment, p = 2.4×10^−34^). Results from both studies did not only overlap in the list of genes associated to smoking but also in the magnitude and the direction of the effects (Pearson correlation coefficient of 0.72 between the 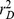 estimated in GHS and the corresponding correlation values reported in [8]) (Figure S1).

### Association between plaques and smoking conditional on a single gene expression

In a second step, we investigated whether single gene expressions might mediate the effects of smoking on plaques. For this purpose, we modeled plaques as a function of smoking and each single gene expression. In this one-dimensional scan, no gene could entirely explain the association between plaques and smoking by conditioning on its expression. *SASH1* was the gene that, once accounted for, contributed to the largest reduction in the covariation between plaques and smoking. *PTGDS* and *PPARG* were the second and third genes contributing the most to this reduction (Table S3). These results suggested that, not unexpectedly, the underlying link between smoking and plaques might involve more complex networks, including several genes and/or hidden variables. Next, we devised an approach to explore a more comprehensive set of models on multiple genes and other variables at a time.

### Clusters of genes associated to plaques or smoking

Unsupervised gene clustering was used to reduce the dimensionality of the data before testing causal models involving multiple genes. Prior to this, we reduced the set of gene expressions by considering only genes that were significantly associated to smoking or to plaques when tested either separately or jointly (see Methods). This constituted a set of 3,960 probes. The probe with the highest variance in intensity across samples was chosen for each gene, leaving a set of 3,368 distinct gene profiles associated to plaques or smoking.

Independent component analysis (ICA) was used to identify patterns of co-expression in this set of 3,368 genes. ICA is an efficient algorithm that factorizes a matrix of multivariate data into a mixing matrix **A** of *patterns* for independent “hidden” components causing correlation among variables and an **S** matrix of *signatures*, which are vectors of coefficients associating variables (genes) to components (see Methods and [20] for details and [21] for a recent application to gene expression data). A pattern is a linear combination of gene expressions whose level varies among individuals. A signature is a vector of the contributions of a component to each gene expression that can be characterized by the genes that are most affected by that component (see *module* below). In the following, the terms *pattern*, *signature* or *module* are used for referring to a component according to the context where it applies (i.e. individuals or genes).

The number of components to extract was determined by a permutation test, which indicated that 59 components could be detected in this dataset (see Methods). Components that did not meet pre-specified quality control criteria were discarded, leaving 29 components for analysis (see Methods and Text S1 for details).

Each component was associated to a specific *module* of genes characterizing its *signature*. A module was defined as the subset of genes that were selected on either tail of the signature distribution by controlling local FDR ≤0.001, as done previously [21]. This resulted in 29 modules of 9 to 125 genes (Table S4). Two modules were enriched in GO pathways: module 18 (interferon-gamma-mediated signaling pathway) and module 39 (antigen processing and presentation via MHC class II) (Table S5).

### Association of ICA expression patterns with smoking and plaques

As mentioned above, the patterns obtained by ICA factorization are linear combinations of gene expressions whose level can be interpreted as reflecting the “degree of activation” of subsets of co-expressed genes among individuals. Association of expression patterns with smoking or plaques was investigated in a similar fashion to that employed for individual gene expressions, except that we used a Bonferroni-corrected significance threshold (0.05/29 = 0.0017) instead of a FDR. At this significance threshold, 14 of the 29 patterns were associated to smoking and 7 to plaques, 5 being common to both (Table 5).

**Table 5:** Association between ICA gene expression patterns (G), smoking (S) and plaques (P).

Pattern	n	b		G∼S		G∼P		P∼S|G	
		S	P	p-value	r^2^_D_	p-value	r^2^_D_	p-value	r^2^_D_
Pattern43	34	+	+	**4E-166**	0.56	**4E-07**	0.03	**4E-06**	0.02
Pattern21	36	−	−	**2E-23**	0.10	**7E-06**	0.02	**4E-14**	0.06
Pattern51	9	−	−	**5E-11**	0.05	**4E-06**	0.02	**2E-16**	0.07
Pattern39	47	+	+	**8E-10**	0.04	**1E-04**	0.02	**8E-17**	0.07
Pattern29	98	+	+	**3E-15**	0.07	**0.000**	0.02	**5E-17**	0.07
Pattern18	98	+		**7E-20**	0.09	3E-02	0.01	**1E-17**	0.08
Pattern11	141	−		**3E-23**	0.10	0.005	0.01	**4E-18**	0.08
Pattern54	17		−	2E-02	0.01	**0.000**	0.02	**1E-18**	0.08
Pattern34	58	+		**0.000**	0.03	1E-02	0.01	**3E-19**	0.08
Pattern28	70			0.007	0.01	0.032	0.00	**2E-19**	0.08
Pattern15	14	+		**6E-04**	0.01	0.200	0.00	**9E-20**	0.09
Pattern45	51			7E-02	0.00	0.413	0.00	**8E-20**	0.09
Pattern36	64	−		**0.000**	0.03	0.477	0.00	**7E-20**	0.09
Pattern19	115			2E-02	0.01	0.425	0.00	**5E-20**	0.09
Pattern52	49	+		**0.001**	0.01	0.127	0.00	**4E-20**	0.09
Pattern41	45			1E-01	0.00	0.849	0.00	**4E-20**	0.09
Pattern14	67	−		**0.000**	0.01	0.815	0.00	**4E-20**	0.09
Pattern12	103			0.902	0.00	0.179	0.00	**4E-20**	0.09
Pattern49	35			0.941	0.00	0.585	0.00	**3E-20**	0.09
Pattern58	23			0.027	0.01	0.934	0.00	**3E-20**	0.09
Pattern33	43			0.971	0.00	0.073	0.00	**2E-20**	0.09
Pattern17	31			0.791	0.00	0.651	0.00	**2E-20**	0.09
Pattern27	13			0.121	0.00	0.283	0.00	**2E-20**	0.09
Pattern23	40			9E-01	0.00	0.360	0.00	**2E-20**	0.09
Pattern30	97	+		**0.000**	0.01	0.936	0.00	**2E-20**	0.09
Pattern42	71			0.138	0.00	0.038	0.00	**2E-20**	0.09
Pattern4	125			0.106	0.00	0.203	0.00	**2E-20**	0.09
Pattern31	85		+	0.072	0.00	0.006	0.01	**3E-21**	0.09
Pattern48	22	−		**6E-06**	0.02	0.071	0.00	**1E-21**	0.09

n: number of genes in the pattern-specific module, b: sign of the regression coefficient of G on S and G on P, respectively. All models included age, sex and the first 6 SVD components. Only the 29 patterns that passed quality control are shown. P-values ≤0.05/29 = 0.0017 are in bold. Genes are ranked by increasing r^2^_D_ associated to S in the model P∼S|G, which corresponds to decreasing reduction of the amount of covariation between smoking and plaques explained by pattern expression.

Worthy of note, ICA was able to recover a strong signature of smoking effects on gene expression in monocytes (pattern 43), as reflected by the high proportion of variance of that pattern (56%) explained by smoking (Table 5). The module associated to pattern 43 comprised 34 genes listed in Table S4. At the core of module 43 was *SASH1*, which had the highest coefficient for signature 43 in the **S** matrix and therefore was the most correlated to the overall pattern of expression.

Significant overlap with genes associated to smoking in lymphocytes [8] was tested for each ICA module in the same manner as for the full set of genes associated to smoking (above). Six of the 29 modules were significantly enriched for genes associated to smoking in lymphocytes (Table S6). The most enriched module was module 43, for which 12 of the 34 genes were also observed in lymphocytes (OR = 27.35; p = 9.1×10^−13^).

As in the single-gene case, no single pattern was able to entirely account for the association between smoking and plaques. The top-ranking pattern by amount of covariation explained between *P* and *S* was pattern 43 (Table 5). When this pattern was introduced in the model relating plaques to smoking, the proportion of variability of plaque counts explained by smoking (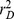) decreased from 8.8% to 2.3%.

### Selection of expression patterns with potential causal role in the relationship between smoking and plaques

**Figure 2:**
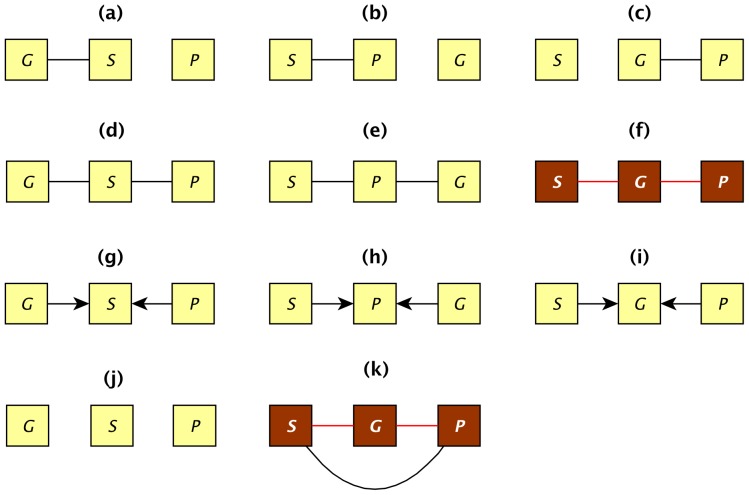
Graphical models for equivalence classes tested among smoking, gene expression and atherosclerotic plaques. Variables are represented by squares and causal associations are indicated by directed edges among nodes. Undirected edges indicate bidirected edges. The two classes colored in brown represent the causal models of interest where gene expression (*G*) mediates the association between smoking (*S*) and plaques (*P*). In class (f), the covariation between *S* and *P* is entirely explained by *G*, whereas in class (k), there is residual covariation between *S* and *P* after conditioning on *G*.

Though no single pattern could entirely explain the relationship between smoking and plaques, we sought to determine whether some ICA patterns may show evidence for a causal effect partially mediating the link of smoking to plaques. For this analysis, we used graphical modeling (Methods). For each expression pattern, the best-supported causal model involving smoking (*S*), plaques (*P*) and gene expression pattern (*G*) was selected by a likelihood-based model selection approach. All equivalence classes of graphical models among these three variables were enumerated (Figure 2). Two classes, *f* and *k,* were of primary interest because both comprise models with a path *S*→*G→P*, which is the causal relation of interest. Under model class *f*, all the association between smoking and plaques can be explained by the effect of one single pattern. In model *k,* the pattern does not explain all the covariation between smoking and plaques, but it is associated to both (Text S1). Maximum likelihood was used to identify the equivalence class that was best supported by the data for each pattern. The process was repeated for 1000 bootstraps of the data to account for uncertainty in model selection.

**Figure 3:**
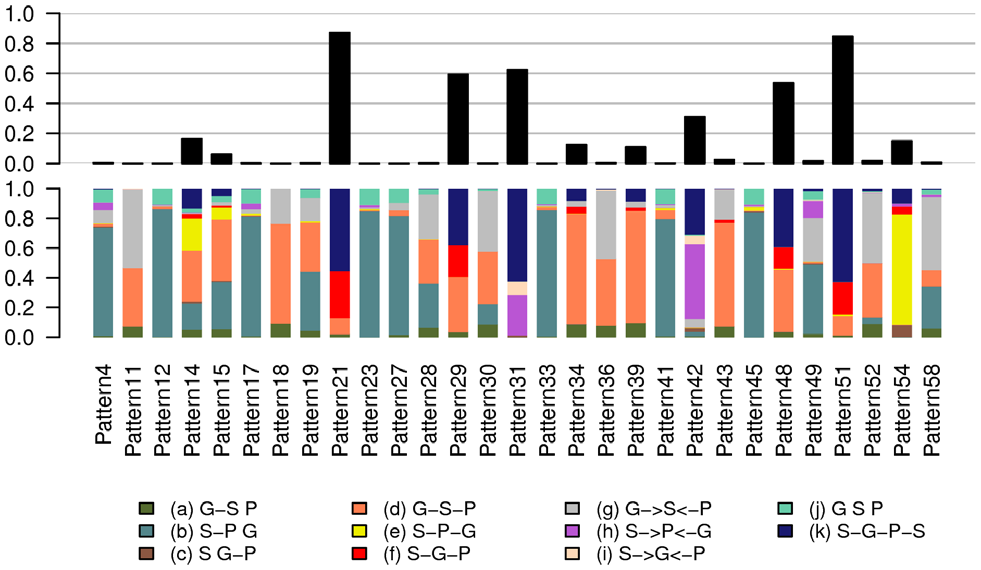
Probability of selection of the different causality models for each ICA expression pattern. Probability was estimated from 1000 bootstraps of the data. The bottom of the graph shows the probabilities of the 11 models described in Figure 2. The top of the graph shows the sum of the probabilities for models (f) and (k) representing the causal classes.

The results of causal model inferences are summarized in Figure 3 and Table S4 (spreadsheet “Causality”). The probabilities of the different models across the 1000 bootstraps for each pattern are shown in bottom half of plot in Figure 3. The probability of selecting a model from a *causal class* was defined as the sum of probabilities for the model classes *f* and *k* (top half of plot in Figure 3). According to this criterion, 4 patterns (21, 29, 31 and 51) had a relatively high support for causality (probability ≥0.6). Worthy of note, pattern 43, the one the most influenced by smoking, was associated with the model *G–S–P* with a probability of 0.7, which is incompatible with *S*→*G→P* causality.

### Inference of the gene network underlying ICA expression patterns

To further characterize the ICA patterns showing some support for causality, we inferred the topology of the networks underlying patterns. For each pattern, the network was constructed from the subset of genes composing the module specific to that pattern. We applied the PC algorithm 1 to discover the skeleton of conditional independencies (algorithm 1 in [22]; see Methods). The network was represented as an undirected graph. To decrease the possibility of hidden variables for the network, we considered in these analyses all cardiovascular risk factors that were associated to each pattern by stepwise regression (Table S7). This is important to avoid spurious edges resulting from untested confounding variables. To assess uncertainty in the inference, the process was repeated 1000 times by bootstrapping individuals and the proportion of data samples that recovered an edge was recorded. We considered only edges with a recovery probability ≥0.6 (see Methods). Graphic representations for all networks are found in Text S2.

### Networks selected by causality test

For the 4 modules with suggestive support for causal effects (21, 29, 31 and 51), we estimated the minimum path(s) between smoking and plaques, starting from each gene directly connected to smoking. Pattern 31 did not reveal any path because no gene was connected to plaques in more than 60% of bootstraps.

**Figure 4:**
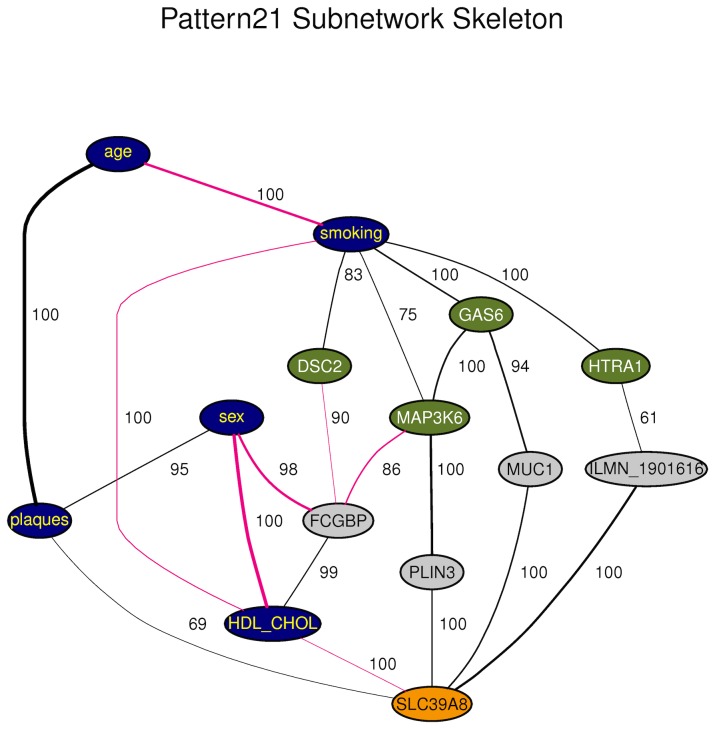
Subnetwork of PC skeleton for Module 21. This graph represents a consensus network from 1000 bootstraps. Edges among variables are drawn if detected in at least 60% of bootstrapped samples. The recovery percentages are indicated to the right of the medial section of each edge. Line thickness is proportional to the edge's partial correlation. Black edges denote positive and pink edges negative partial correlations. Plaques and risk factors are in blue. Genes directly connected to smoking are in green and those directly connected to plaques are in orange. Other genes are in gray. Only genes that are involved in the shortest paths connecting smoking to plaques are shown. The full network for this and other patterns are found in Text S2.

For pattern 21 (Figure 4), the different paths connecting smoking to plaques involved four genes directly connected to smoking (*MAP3K6, GAS6, HTRA1* and *DSC2*) and only one gene directly connected to plaques (*SLC39A8*) (Table 6). Therefore, the *SLC39A8* gene funneled all information paths between smoking and plaques in this network. *SLC39A8* expression level was positively correlated to plaques and smoking was positively correlated to all the genes in the cluster, suggesting that an up-regulation of genes of the cluster was associated to increased atherosclerosis extent. Additionally, *SLC39A8* was negatively correlated to HDL-cholesterol levels (∼1 probability), which is consistent with the protective role of HDL-cholesterol in atherosclerosis.

**Table 6:** Shortest gene paths connecting smoking to plaques.

Pattern	Paths
Pattern21	smoking - GAS6 - MUC1 - SLC39A8 - plaques
	smoking - HTRA1 - ILMN_1901616 - SLC39A8 - plaques
	smoking - MAP3K6 - PLIN3 - SLC39A8 - plaques
	smoking - DSC2 - GK - FCGR1A - MUC1 - SLC39A8 - plaques
Pattern29	smoking - DHRS9 - ACOX2 - COQ2 - RASD1 - plaques
	smoking - FAM20C - NEK6 - ACOX2 - COQ2 - RASD1 - plaques
	smoking - FPR3 - AVPI1 - RASD1 - plaques
	smoking - CXCL16 - UTP6 - FAR2 - RASD1 - plaques
	smoking - TBC1D8 - CMTM4 - FFAR2 - RASD1 - plaques
	smoking - PDE4B - PFKFB3 - COQ2 - RASD1 - plaques
	smoking - PTGFRN - CDC42EP2 - COQ2 - RASD1 - plaques
Pattern51	smoking - CYP1B1 - TMEM136 - plaques
	smoking - HOXA10 - MYB - CYP1B1 - TMEM136 - plaques

Only networks passing the causality tests are shown.

In pattern 29, there were 7 genes directly connected to smoking (*CXCL16, DHRS9, FAM20C, FPR3, PDE4B, PTGFRN*, and *TBC1D8*) and only one gene connected to plaques (*RASD1*) (Table 6). *RASD1* was positively correlated to plaques, although this association was recovered with probability of only 0.64. This was a large network with 98 genes (Table S4). However, smoking and plaques were separated by a relatively low number of genes where the 7 paths had between 3 to 5 connecting genes (Table 6).

Pattern 51 was the smallest (15 nodes) and among the most interconnected networks, with an average number of connections per node of 4.3. Among the different paths connecting smoking to plaques, 2 genes were directly connected to smoking (*CYP1B1* and *HOXA10*) and only one gene was directly, negatively, connected to plaques (*TMEM136*) (Table 6).

### *PPARG* network

*PPARG* was the gene showing the strongest association to plaques and the third in the reduction of the *r^2^_D_* of smoking to plaques. In addition, this gene is known to be involved in atherosclerosis [23]. This prompted us to examine in more details the network(s) comprising *PPARG*. Actually, *PPARG* was only present in module 42, which included 71 genes (Table S4). Pattern 42 was not significantly associated to smoking or plaques (Table 5). However, causality testing gave inconclusive results, with two model classes, *h* (*S→P←G*) and *k* (*S—G—P—S*), alternatively selected by bootstrapping with probabilities 0.5 and 0.31, respectively (Table S4). In model *h* which had the best support, smoking and pattern expression were associated only when conditioned on plaques, meaning that both have causal effects on plaques but independently from one another. Conversely, the topology of the network suggested that the shortest path from smoking to plaques was through *PPARG* only (Text S2). This path, which was recovered in 96% of bootstraps, supported a causal effect of *PPARG*. This discrepancy between *PPARG* and pattern 42 might be explained by the fact that the contribution of pattern 42 to *PPARG* was weak (ranking 67 out of 71 genes of the module) and therefore, the behavior of *PPARG* did not exactly match that of the entire pattern.

## Discussion

We present results from a genome-wide survey of gene expression in monocytes that revealed widespread effects from smoking, with >3000 genes either over- or under- expressed in smokers. Because no information was collected about number of cigarettes smoked per day, we could not test dose-dependence of the effects on gene expression. Due to the non-stringent FDR adopted, the list was rather large because our primary objective was not to miss any gene of potential interest. The list of smoking-associated genes showed significant overlap with those observed in lymphocytes from a large cohort of Mexican Americans [8] indicating high robustness of smoking effects across different circulating cell types and genetic background. By contrast, the number of gene expressions associated to atherosclerosis was much lower (236 genes), which is probably explained by the fact that atherosclerosis is a complex and distal phenotype with multiple genetic and non genetic determinants.

As expected, a one-dimensional scan across the transcriptome revealed that no single gene could explain the association between smoking and plaques, leading us to search for networks of genes that might be more relevant. Using ICA, we identified 29 patterns of co-expressed genes, 14 of which were strongly associated to smoking. Two patterns were enriched in functional GO categories (interferon-gamma-mediated signaling pathway and MHC class II antigen processing) but none of these two patterns was related to atherosclerosis after conditioning on smoking. Worthy of note, one of the patterns (pattern 43 driven by *SASH1*) could be interpreted as a robust signature of the impact of smoking on the transcriptome of circulating blood cells, as demonstrated by the substantial overlap of smoking-associated genes between monocytes and lymphocytes [8]. Actually, all the genes directly connected to smoking in network 43 (i.e. *SASH1*, *MMP25*, *P2RY6*, *FUCA1*, *PID1*, *DTNA*, *GFRA2*, *CLEC10A* and *PTGDS*) had been previously identified as the strongest correlates of smoking in GHS [9]. Surprisingly, none of these genes was found differentially expressed in a recent experimental study performed in a human monocytic cell line (THP-1 cells) exposed to cigarette smoke extract [24]. This discrepancy suggests that *in vivo* chronic exposure to cigarette smoke may have a different impact from *in vitro* acute exposure, in particular because of the important role played *in vivo* by the lung, kidney and liver in metabolizing xenobiotics.

We then tested causality by graphical modeling. Actually, two model classes, *S–G–P* and *S–G–P–S* contained the causal path of interest *S→G→P*. Although both of these graphs describe multiple Bayesian networks, *a priori* information can be used to favor only a few of them. Indeed, genes expressed in monocytes are unlikely to affect smoking behavior, eliminating *S*←*G* edges. Supporting this assumption, only three genes across all networks have been reported by GWAS to be associated to smoking behavior and none of them were in modules selected for causal effects (Table S8). On the other hand, by excluding ex-smokers, we reduced the possibility that smoking behavior might be modified by the presence of atherosclerosis, making *S*←*P* edges less likely (see Text S1 for details). Therefore, the networks most likely underlying the two graphical models of interest were *S→G→P* for the two-edge case and *S→G→P*←*S* or *S→G*←*P*←*S* for the three-edge case.

We identified four patterns of expression (21, 29, 31, and 51) that were compatible with an effect of expression partially mediating the relation between smoking and atherosclerosis. Pattern 31 did not have a gene network topology consistent with any gene mediating smoking to plaques effects. Pattern 29 was associated to a large module (94 genes) with some support for causal effects (0.59 probability). All the paths in this network were connected to plaques through a single gene, *RASD1.* This gene, whose exact function is unknown, encodes a Ras-related protein stimulated by dexamethasone, a drug with anti-inflammatory and immunosuppressive actions. Because of the relatively low probability of causality of pattern 29 and the modest recovery of the *RASD1–*plaques edge (0.64 frequency), caution is needed in the interpretation of this result.

Pattern 51 had a relatively high support for causality (0.85 probability). In this network, smoking was directly connected to 2 genes, *CYP1B1* and *HOXA10*, which both have relevance to atherosclerosis and smoking. *CYP1B1* encodes a member of the cytochrome P450 protein superfamily that localize to the endoplasmic reticulum and metabolize procarcinogens including polycyclic aromatic hydrocarbons in tobacco-smoke [25]. *CYP1B1* expression showed the strongest difference in placenta from smoking and nonsmoking women [26] and was increased in THP-1 cells after *in vitro* exposure to cigarette smoke [24]. In endothelial cells, *CYP1B1* expression is regulated by shear stress and is associated to anti-atherogenic effects [27] and decreased oxidative stress [28]. *HOXA10* encodes a transcription factor associated to cell proliferation in the monocytes cell lineage [29] and repression of PHOX genes involved in oxidative stress [30]. It is expressed in the endothelium in a location-dependent manner, with lower expression in atheroprone than in atheroresistant arteries [31], [32]. *HOXA10* expression in endometrium has been shown to be directly affected by cigarette-smoke extract both in humans and mice [33]. Therefore, there is support in the literature linking at least two genes of the network to smoking and plaques. However, *TMEM136,* which was the only gene of the network directly connected to plaques, was expressed at low levels in monocytes, only exceeding detection threshold in 5% of male non-smokers and a few individuals outside this group. *TMEM136* encodes a transmembrane protein of unknown function. Therefore, it is possible that association between plaques and this gene is instead reflecting association to a different unobserved network member.

Pattern 21 which had the highest support for causality (0.87 probability) also appeared to have the highest relevance in the context of smoking-induced atherosclerosis. In this network, connection to plaques was mediated by *SLC39A8* (aka ZIP8), a transmembrane zinc transporter. Genetic variants in *SLC39A8* have previously been associated to several cardiovascular risk factors such as HDL-cholesterol [34], [35], blood pressure [36], [37], obesity [38], and activation of plasminogen [39]. In addition to being connected to plaques, *SLC39A8* was directly connected to HDL-cholesterol in the network (Figure 4), a result consistent with the association found with *SLC39A8* genetic variants [34], [35] and supporting a causal role of *SLC39A8* in atherosclerosis. However, here we provide evidence for an effect on plaques not completely mediated by HDL-cholesterol, since a direct plaques–*SLC39A8* edge was recovered with 0.69 probability. *SLC39A8* is known to have a cytotoxic role by intracellular transport of cadmium, a toxic heavy metal and carcinogen that is abundant in cigarette smoke [40], [41]. In lung epithelia, *SLC39A8* expression is increased by TNFα, a pro-inflammatory cytokine that is abundant in smoker's lung [42]. In addition, the cadmium-mediated toxicity induced by cigarette smoke has been shown to be enhanced through NF-κB-mediated activation of *SLC39A8* expression [42].

The shortest path from smoking to plaques in network 21 was smoking–*GAS6*–*MUC1–SLC39A8*–plaques. *MUC1* (mucin 1) encodes a membrane protein involved in cell adhesion and signal transduction, not previously associated to smoking effects or atherosclerosis. By contrast, *GAS6* (growth arrest-specific gene 6) has a strong relevance to atherosclerosis. It belongs to a family of vitamin K-dependent coagulation proteins and has a pleiotropic role in atherosclerosis, with pro- and anti-atherogenic effects [43]. In human atherosclerotic plaques, which are the focus of the present study, *GAS6* has been shown to be expressed mainly by vascular smooth muscle cells and to have an anti-inflammatory action by stimulating the anti-inflammatory cytokine TGFß and inhibiting expression of TNFα [44]. Therefore, TNFα may be a molecular signal underlying the correlation between *SLC39A8* and *GAS6*, but this hypothesis needs to be confirmed. *GAS6* was also among the genes reported to be up-regulated in the placenta of women smoking during pregnancy [26]. To the best of our knowledge, *GAS6* and *SLC39A8* have not been connected before in the context of atherosclerosis and their functional link needs to be experimentally confirmed.

Because ICA was performed on a subset of genes pre-selected by their association with smoking or atherosclerosis, we cannot exclude the possibility that we missed some genes that could be important nodes in the networks subsequently identified. However, not doing this pre-selection would have led to a larger number of ICA components (91 components were actually detected in our previous ICA application in the complete GHS expression dataset [21]), most of them being irrelevant for the problem under study. Also, we cannot exclude the possibility of spurious edges in networks resulting from untested confounding variables.

Networks discovered in this study may represent diverse mechanisms of gene-by-gene co-expression. For instance, expression of genes may be co-regulated by a signaling pathway in a single cell type or they may represent coordinated variation in the proportion of cell population subclasses of monocytes. Both possibilities are equally interesting since causal effects may be mediated by either mechanism. For instance, HDL levels modulate monocytes proliferation and activation, changing the composition of the myeloid cell lineage, which is thought to explain in part its anti-inflammatory and athero-protective effects [45], [46]. Further studies are needed to determine what specific molecular mechanisms underlie the correlation patterns reported here. Additionally, replication of candidate gene networks in independent datasets should be performed before laborious functional studies are undertaken.

A word of caution is in place about inferring causal associations from observational data. Direct associations in causal graphs are appropriate only in the context of the variables that are included in the system, i.e. the variables that were measured. Causal graphs are considered complete only in the sense of common causes between variables but they do not include all causes of variables [47]. Therefore, a direct causal association may represent the net effect of a large number of direct causal associations among variables that were not measured and that mediate the effects among the variables that were observed [48]. A causal graph may change if variables are removed or new ones are included. Furthermore, the real causal associations that can be inferred from graphical models are not in the edges but in the lack of edges between variables, that is, two variables are not causally associated given that we account for the other variables in the system. Instead the presence of an arrow only indicates the possibility of a causal association, which has to be determined from data [49]. The method used here can only identify networks, which in the context of the variables measured, present a correlation structure that is not incompatible with the causal effects of interest *S→G→P*. An effort was made to include all other variables that may be relevant in the system, but of course there is no guarantee that all relevant variables were included. In addition, although the method does not always allow identifying the best model, providing the best fitting class of models is an honest and useful summary of the information encoded in these data.

In conclusion, we have used a graphical modeling approach to investigate the potential role of gene expression in monocytes in mediating smoking effects on atherosclerosis. The analytic approach implemented here allowed discriminating among competing causal models affecting multiple genes and revealed gene networks that included multiple members with known causal roles in atherosclerosis or mediation of smoke-tobacco effects. To the best of our knowledge, this is the first application of causal inference on gene modules rather than individual genes. Our results put together previously unconnected genes that led to the formulation of new hypotheses about potential molecular mechanisms linking smoking effects to atherosclerosis phenotypes. Therefore, inspection of the correlation structure of risk factors, gene expression and atherosclerosis, revealed candidate genes that would have been missed by looking at strength of gene-phenotype associations alone.

## Materials and Methods

More details are provided in Text S1.

### Subjects

Study participants of both sexes aged 35–74 yr, were successively enrolled into the GHS, a community-based cohort study in the Rhein-Main region in western mid-Germany. Participants were of European origin. More details about the GHS study are provided in [9]. There were 1,536 individuals with microarray expression data that passed quality control tests. Nine individuals with missing number of plaques were removed. Smoking status was recorded by interview at recruitment. Current smokers (individuals smoking ≥1 cigarette per day since at least 6 months before recruitment, *n* = 248) and nonsmokers (individuals who had never been smoking over a period of at least 6 months, *n* = 688) were used for analyses. Occasional smokers (*n* = 42), ex-smokers (*n* = 547) and individuals with missing information on smoking status (*n* = 2) were excluded.

### Ethics statement

All subjects gave written informed consent. Ethical approval was given by the local ethics committee and by the local and federal data safety commissioners (Ethik-Kommission der Landesärztekammer Rheinland-Pfalz 22/03/2007 Number 837.020.07 (5555)).

### Evaluation of the number of atherosclerotic plaques

Intima-media thickness (IMT) in the common carotid arteries was assessed with an ie33 ultrasound system (Philips, NL) using an 11 to 3 MHz linear array transducer. Measurements were performed by an experienced technologist and evaluated in the QLab software (Philips, NL). The presence of an atherosclerotic plaque was determined by an increment of 1.5 mm or more in IMT when compared to a region without plaque 1 cm before the carotid bulb, averaging from left and right carotids. Plaques were counted in the common, external, and internal carotid arteries on both left and right sides [9]. The phenotype considered was the count of plaques summed over both carotid arteries.

### RNA samples processing

Total RNA from circulating monocytes was extracted as previously described [9]. Briefly, monocytes were isolated from blood samples with the RosetteSep Monocyte Enrichment Cocktail (StemCell Technologies, Vancouver, Canada), cells were resuspended in Trizol (Invitrogen, Karlsruhe, Germany), RNA was extracted with the RNeasy Mini kit (Qiagen, Hilden, Germany) and controlled for quality in an Agilent Bioanalyzer 2100 (Agilent Technologies, Boeblingen, Germany).

### Microarray data processing

RNA samples were hybridized to the *Illumina* HT-12 BeadChip v3 (*Illumina*, San Diego CA) containing 48,803 50-mer DNA probes. Probe-mapping to the genome was obtained from ReMOAT annotations [50] in the illuminaHumanv3.db package from Bioconductor v2.8 (http://www.bioconductor.org). There were 13,445 probes with “Bad” scores for genome alignment, which were discarded. Probes were annotated with RefSeq and EntrezGene ids using the org.Hs.eg.db package from Bioconductor. Of the remaining 35,358 probes, 28,515 were annotated to Ensembl transcripts and 28,137 to EntrezGene ids. For the purpose of counting number of genes throughout, unannotated (but of good quality) probes were considered as targeting distinct genes. Note that probe annotations from *Illumina* were not used to discard probes, which is different from our previous analyses on this dataset [9], [21]. However, 98% of probes that were removed in our previous analyses were also removed by at least one of the filtering steps applied here.

Bead-level data were processed with BeadStudio (*Illumina*, San Diego CA) to perform quality control and summarization of intensity values at probe level. Data were further processed by quantile normalization. An *arcsinh* transformation was applied to stabilize the variance [51]. A transcript was considered detected when the normalized intensity reported by its targeting probe was significantly above that of negative control probes on the same array (detection p-value<0.04). Probes with “detected” calls in ≥5% of samples within any smoking/sex group were considered for analysis, representing 23,214 probes. All analyses were performed with R v2.13 [52].

### Correction for technical sources of variability in gene expression

Major components of variance in the gene expression dataset were identified by singular value decomposition (SVD) using the La.svd function in R. The six largest components were not associated to any individual characteristic, and therefore were thought to reflect systematic effects from sample-processing protocols. The first 6 SVD components were then used as surrogate variables of technical effects and were adjusted for in expression analyses [53]. In addition, potential sample contamination with B and T cells, and megakaryocytes was corrected as previously described [21] (Text S1).

### Modeling the number of plaques

In analyses modeling the number of plaques as dependent variable, we used a negative binomial (NB-2) distribution [54]. Other distribution functions were considered but not used (see Text S1 for details). The negative binomial model was fitted by the *glm.nb* function with a log link using the R MASS package. A pseudo-*R^2^* coefficient was defined as 
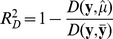
, where 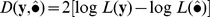 is the deviance around means predicted by model 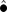, **y** is the saturated model (one coefficient per observation), 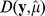 is the deviance in the full model and 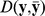 is the deviance in the model with an intercept only [55]. In GLM models of Gaussian response variables, this reduces to the *R^2^* coefficient of multiple determination. Similarly, a pseudo-coefficient of partial determination was computed to measure the marginal contribution of one independent variable in predicting a response when contribution from all other variables in the model is accounted for [56]. This coefficient for an independent variable *x* was defined as 
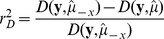
, where 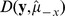 is the deviance of a reduced model with *x* dropped. Model selection was performed by the Bayesian Information Criterion 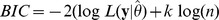, where 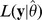 is the maximum likelihood of the data given parameters 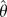, *k* is the number of parameters in 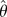 and *n* is the number of observations. When comparing different negative binomial models, the dispersion parameter α was held constant and fixed at the estimated value from the model with age, sex and smoking effects (α = 1.34). Individuals with missing values for any variables being considered were removed before model fitting.

### Single gene expression association analysis

Analysis of expression data was performed at the probe level. Association of a single probe expression with plaques counts and smoking was performed by linear regression analysis where the dependent variable was the probe expression level and the independent variables were plaques and smoking, tested either individually or simultaneously. In these models, the variable considered for plaques was *ln*(plaques+1). All models were systematically adjusted for age, sex and the 6 first SVD components. Linear models were tested by the *glm* function in *R*. An FDR ≤0.1 was used for selecting the probes associated to plaques or to smoking [57]. See Text S1 for details.

The list of 3,368 distinct gene expressions associated to smoking was compared to the list of 323 gene expressions previously reported to be associated to smoking in lymphocytes [8]. This study used the *Illumina* Human WG-6 v1 microarray platform, which is an earlier version of the HT-12 used in GHS. Updated gene annotations were obtained from Bioconductor. There were 19,614 unique genes in WG-6 of which 17,243 were also queried in HT-12. Of these, 13,707 were detected in monocytes of GHS, which were used as the reference set. Gene set enrichment analysis was performed by the Fisher's exact test implemented in the *fisher.test* function in R.

### GO enrichment analysis

Gene set enrichment analysis of gene ontology (GO) terms was performed with the *topGO* (v 2.4.0) package in R. GO data were obtained from the *GO.db* Bioconductor metadata package v 2.5.0 (March 2011). Enrichment was tested by a Fisher's exact test using the set of 18,364 unique known detected genes as a reference. In order to account for the redundancy and hierarchy of GO terms, the *weight01* algorithm was used, which is a hybrid between the *elim* and *weight* algorithms described in [58]. Control for multiple testing was done by a Bonferroni correction on the number of GO terms represented in the reference set.

### Covariation between plaques and smoking explained by single genes

To test whether a single gene expression could explain part of the covariation between plaques and smoking, we modeled plaques as a function of smoking and gene expression using the *glm.nb* function. The dispersion parameter *θ* was held constant across genes to a value of 0.75, which was estimated with the *glm.nb* function on the reduced model without gene effect. The strength of the association of smoking before and after including gene expression was tested by the 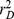 coefficient as described above. The difference between these values was regarded as covariation explained by a single gene.

### Clustering gene expressions by Independent Component Analysis (ICA)

The fastICA algorithm was used to factorize the matrix of 3,368 gene expressions associated to smoking or plaques [20]. A single probe per gene was selected, that was the probe showing the largest variance across samples. This approach avoids the bias that would be introduced by, for instance using the probe with the strongest association to smoking or plaques and favors probes with more information content. Using the mean expression across probes for a gene was not considered because of the added noise that that may result from errors in probe annotation and because the largely uneven number of probes per gene would dramatically affect the distribution of technical error across genes. All unannotated probes were kept.

Normalized data from each probe were centered and standardized. SVD was initially performed to determine the *a priori* number of components for the ICA algorithm as explained in [21]. This number was found to be 59.

The fastICA algorithm identifies major variance components by iteratively estimating the “mixing” matrix **A** that satisfies the equation **X = SA**, where **X** is an *m*×*n* data matrix, **S** is a *m*×*p* matrix of signatures across genes, **A** is *p*×*n* matrix of patterns across samples, *n* is the number of samples (*n* = 936), *m* is the number of genes (*m* = 3,368), and *p* is the number of ICA components set *a priori* (*p* = 59). The iterative algorithm minimizes dependency among signatures (columns of **S**), while maximizing non-Gaussianity, i.e. negentropy, of signature distributions [20]. The fastICA function in the R-package of same name was used [59]. The algorithm was run multiple times to avoid trapping in a local maximum. The results were processed as previously described in [21] to remove components that were not consistently detected across random start points. Briefly, fastICA was repeated 500 times and the best run (with the maximal negentropy) was selected. The stability of components over the 500 runs was calculated. Components that did not meet quality control criteria were discarded (see Text S1 and Tables S9, S10, S11).

For each component, a signature-specific module was defined as the subset of genes on either tail of the signature distribution selected by controlling local FDR ≤0.001 [21]. The association between ICA expression patterns and smoking or plaques was tested in the same way as single gene expression, except that adjustment was made on all risk factors associated to pattern by stepwise regression analysis and not only on age and sex.

### Investigation of causality models

All triplets including smoking (*S*), plaques (*P*), and a gene expression pattern (or a single gene expression) (*G*) were constructed. Potential causal relationships among triplet members were represented as Bayesian Networks, i.e. graphical models, where variables are vertices and causal associations are indicated by directed edges, i.e. arrows, between nodes. The probability function used depended on the distribution of each variable. Smoking was modeled by a binomial, plaques by a negative binomial, and gene expression by a Gaussian distribution function. The inference of network structure and parameters was performed as follows. Each node in the graph was fitted a linear model with its parents as independent variables. Given a graph *M* with variables *X = {X_1_, …, X_N_}* represented as nodes, a BIC score [60] for *M* was computed as *BIC(M) = −2 log(L(X|M))+k log(n)*, where *n* is the sample size, *N* is the number of nodes, *k* is the number of parameters of the model, and *L(X|M)* is the maximum likelihood of the data given graph *M*, which by the *directed Markov property* of the graph, is given by 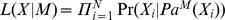. The term 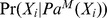 denotes the conditional probability of observation *X_i_* given its set of parents 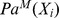 in graph *M*. The graph with the lowest BIC is selected as the most likely model, which produces identical values for equivalent networks [61]. Here, however, because different density functions were used for different variables, equivalent networks may results in slightly different scores. Therefore, we computed BIC scores for all models in a class, which was selected if any network in the class had the minimum score. The probability of selecting a model class, estimated by 1000 bootstraps of the data was used as a measure of confidence in the Bayesian network inference [62].

### Inference of network skeleton

The skeleton of the network connecting genes, smoking, risk factors and plaques was learned by conditional associations using the PC algorithm 1 implemented in the *pcalg* R package v 1.1–4 [22]. Briefly, the algorithm starts with a matrix of marginally associated variables (nodes connected by edges) and for each pair of connected, i.e. adjacent, nodes it successively tests whether the pair becomes independent after conditioning on any group of adjacent nodes. If for any set of conditioning nodes the pair is independent, the edge is removed. The Pearson correlation with significance level of 0.05 was used to test independence. The process was repeated for 1000 data bootstraps and the proportion of samples where the edge is recovered was recorded. Shortest paths between smoking and plaques were inferred with the Dijkstra's algorithm on a reduced graph after removing nodes corresponding to covariates. Whenever more than one shortest path existed, the algorithm chooses one according to a greedy search. The Dijkstra's algorithm implemented in the *sp.between* function of the RBGL package for R was used [63].

Note that although in its full version the PC algorithm (Algorithm 2 in [22]) can asymptotically infer causal associations in a network by directing some of the edges of the skeleton, when applied to samples of finite size the algorithm may lead to inconsistencies that make such inference impossible [64]. This was the case with the present data. Therefore, we decided to limit to inferring the skeleton of significant partial correlations among variables using a first part of the PC algorithm.

## Supporting Information

Figure S1Comparison of the magnitude of smoking effects on gene expression in two independent cohorts. SAFHS: San Antonio Family Heart Study, (Charlesworth *et al.*, 2010); GHS: Gutenberg Health Study (this manuscript). Dots correspond to the 268 smoking-associated genes in SAFHS that were detected in GHS. The x-axis shows the Pearson correlation between gene expression and smoking in SAFHS and the y-axis the signed-square root of the 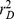 coefficient for smoking in GHS. Genes associated to smoking in GHS are represented by black dots (*n* = 151), others are in gray.(TIFF)

Figure S2 Density of the distribution of the edge recovery proportion from bootstraps for all possible node pairs. Patterns 4 to 28.
(TIFF)

Figure S3 Density of the distribution of the edge recovery proportion from bootstraps for all possible node pairs. Patterns 29 to 48.
(TIFF)

Figure S4 Density of the distribution of the edge recovery proportion from bootstraps for all possible node pairs. Patterns 49 to 58.
(TIFF)

Table S1 Risk factors associated to plaques count, selected by stepwise negative binomial regression.
(DOC)

Table S2 Results of association analysis of expression levels with smoking and plaques for all detected probes (n = 23,214).
(XLSX)

Table S3 Top 20 genes explaining covariation between smoking and plaques.
(DOC)

Table S4 Summary tables and figures for 29 ICA expression patterns that passed quality control.
(XLSX)

Table S5 Gene Ontology categories enriched for gene expressions in specific ICA modules.
(DOC)

Table S6 Enrichment of ICA module gene sets in monocytes for genes associated to smoking in lymphocytes [Charlesworth et al. 2010].
(DOC)

Table S7 Cardiovascular risk factors associated to expression patterns by stepwise regression.
(DOC)

Table S8 Genes with reported association to smoking in GWAS.
(XLSX)

Table S9 Quality control statistics for ICA components of variance.
(DOC)

Table S10 Pearson correlation coefficients between ICA patterns and 6 first SVD components of expression matrix.
(DOC)

Table S11 Pearson correlation coefficients between ICA patterns and surrogate variables for cell contamination by non-monocytic cells.
(DOC)

Text S1 Supporting information: further details of Methods.
(DOC)

Text S2 Network plots for all ICA modules.
(PDF)
