# Supplementary figures and images for: Graphical Modeling of Gene Expression in Monocytes Suggests Molecular Mechanisms Explaining Increased Atherosclerosis in Smokers
Source: PLoS One. 2013 Jan 23;8(1):e50888. doi: 10.1371/journal.pone.0050888 (PMC3553098; doi:10.1371/journal.pone.0050888)

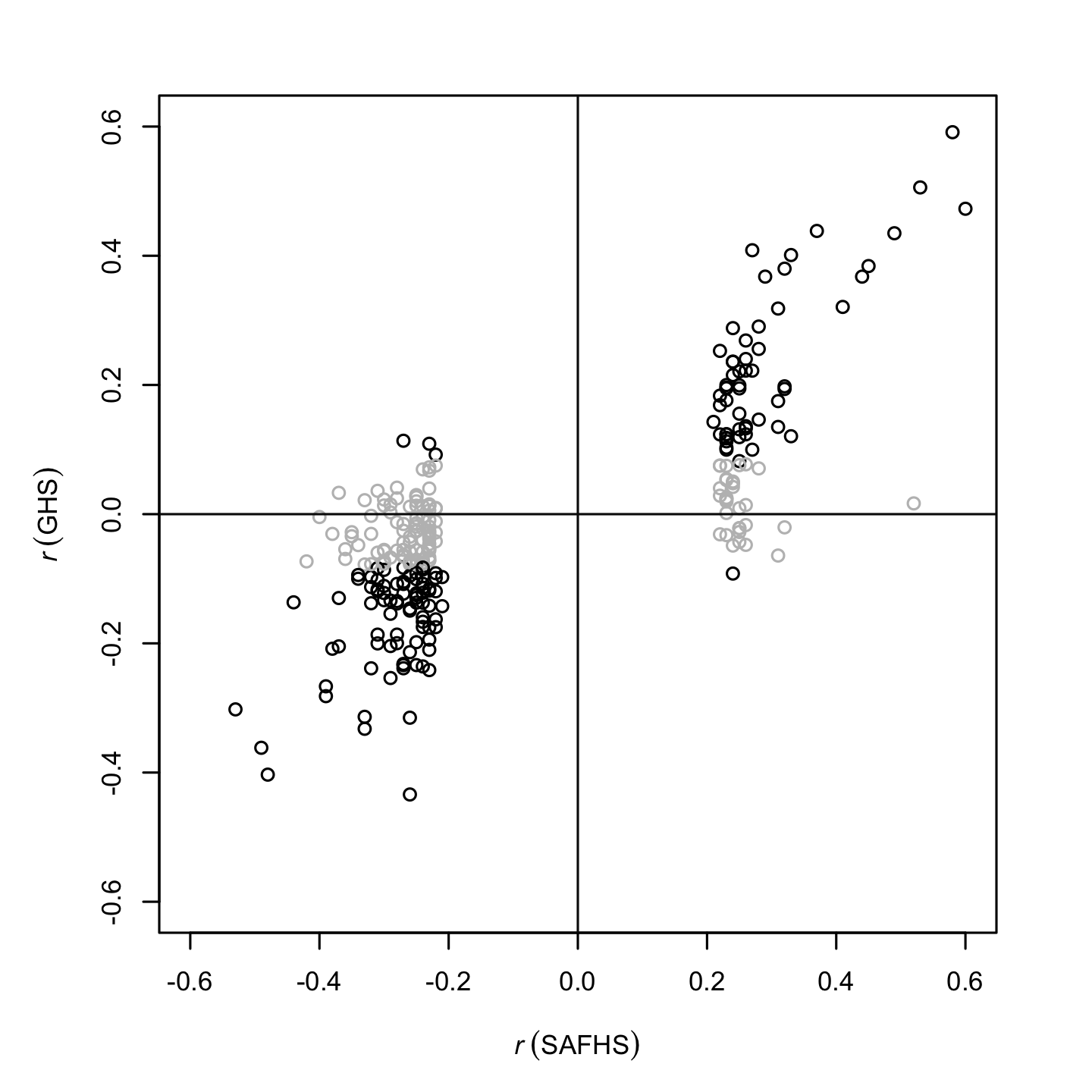

Supplement: Figure S1 — Comparison of the magnitude of smoking effects on gene expression in two independent cohorts. SAFHS: San Antonio Family Heart Study, (Charlesworth et al., 2010); GHS: Gutenberg Health Study (this manuscript). Dots correspond to the 268 smoking-associated genes in SAFHS that were detected in GHS. The x-axis shows the Pearson correlation between gene expression and smoking in SAFHS and the y-axis the signed-square root of the coefficient for smoking in GHS. Genes associated to smoking in GHS are represented by black dots (n = 151), others are in gray. (TIFF) [file pone.0050888.s001.tiff]

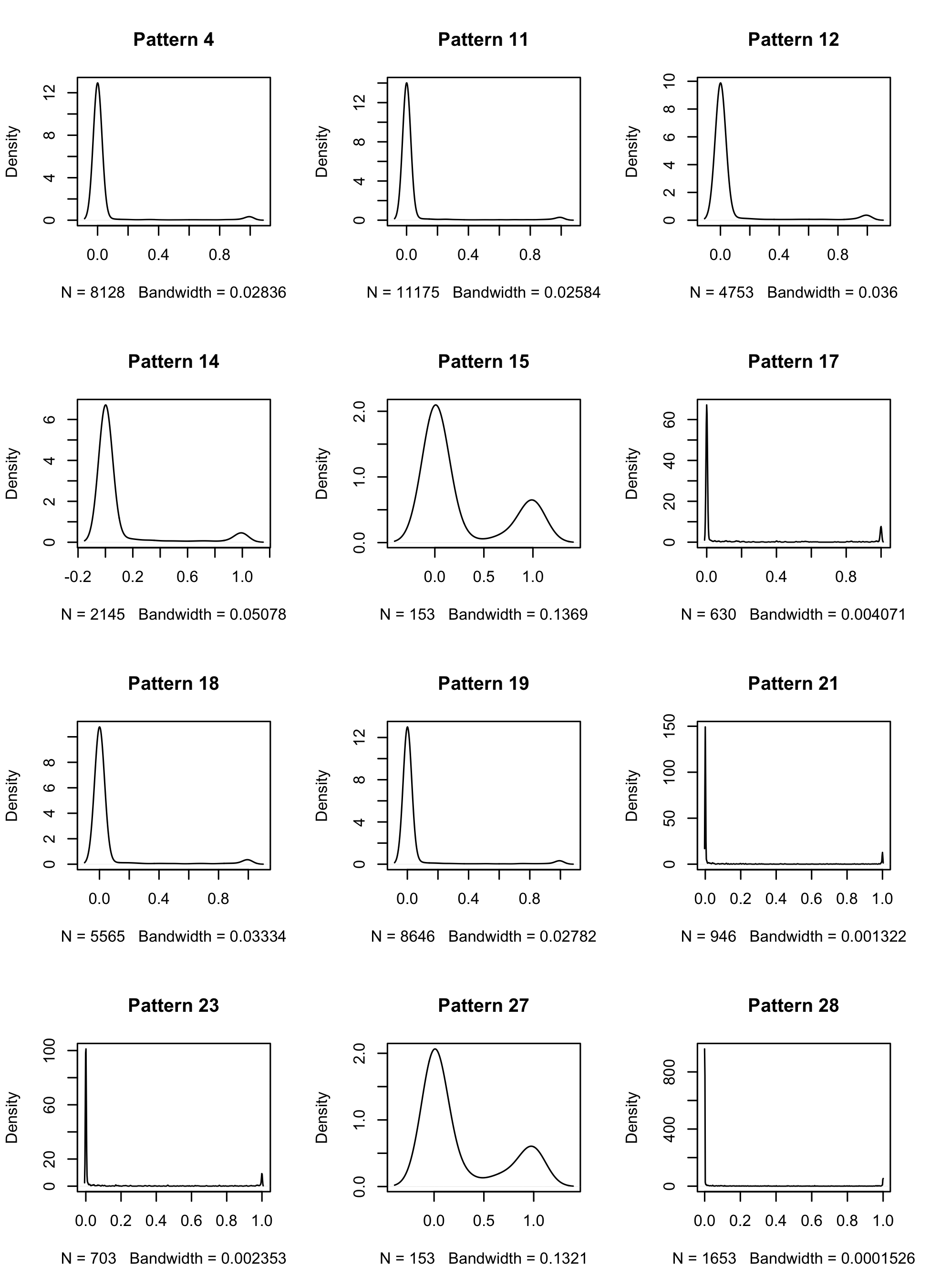

Supplement: Figure S2 — Density of the distribution of the edge recovery proportion from bootstraps for all possible node pairs. Patterns 4 to 28. (TIFF) [file pone.0050888.s002.tiff]

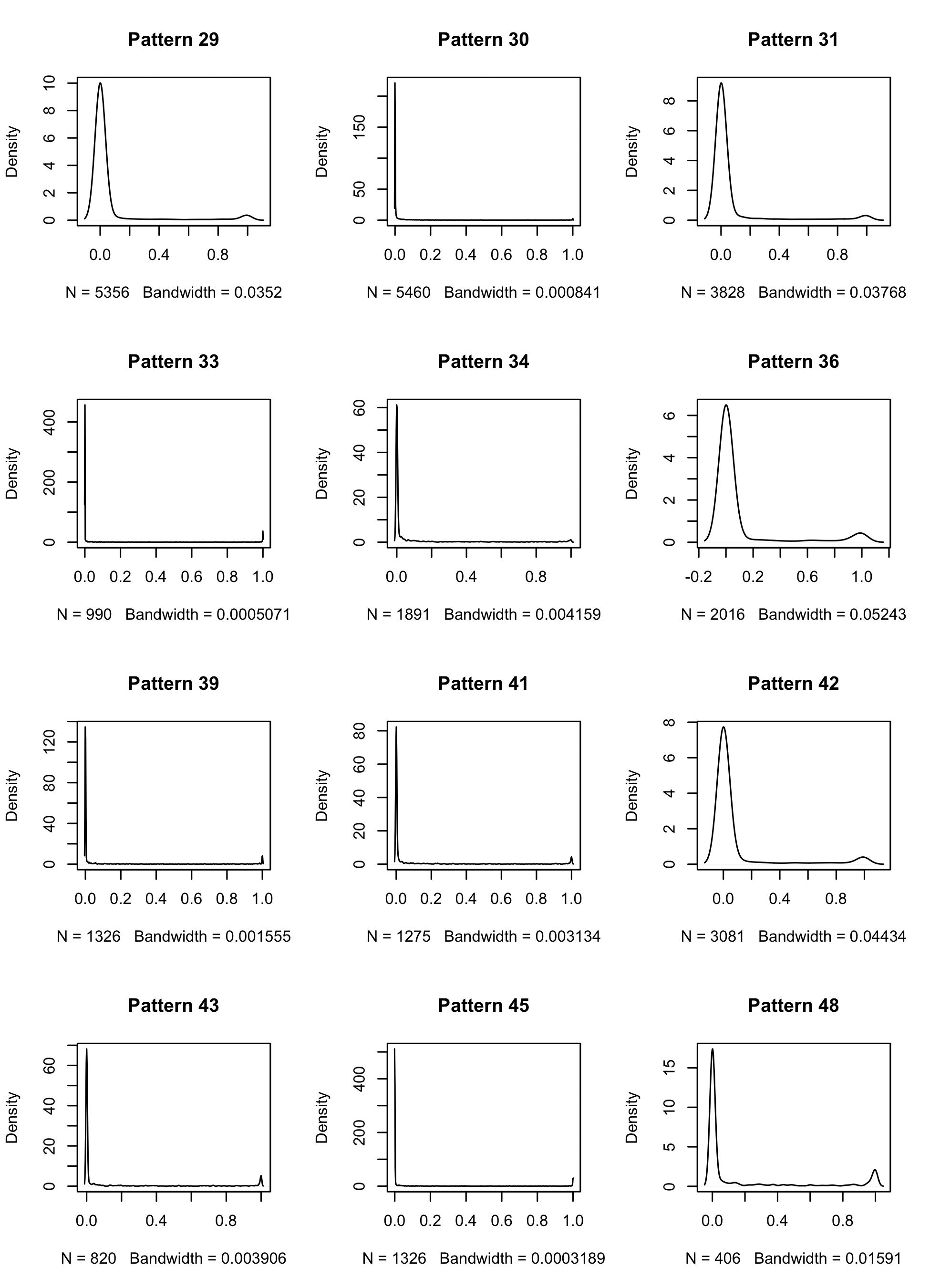

Supplement: Figure S3 — Density of the distribution of the edge recovery proportion from bootstraps for all possible node pairs. Patterns 29 to 48. (TIFF) [file pone.0050888.s003.tiff]

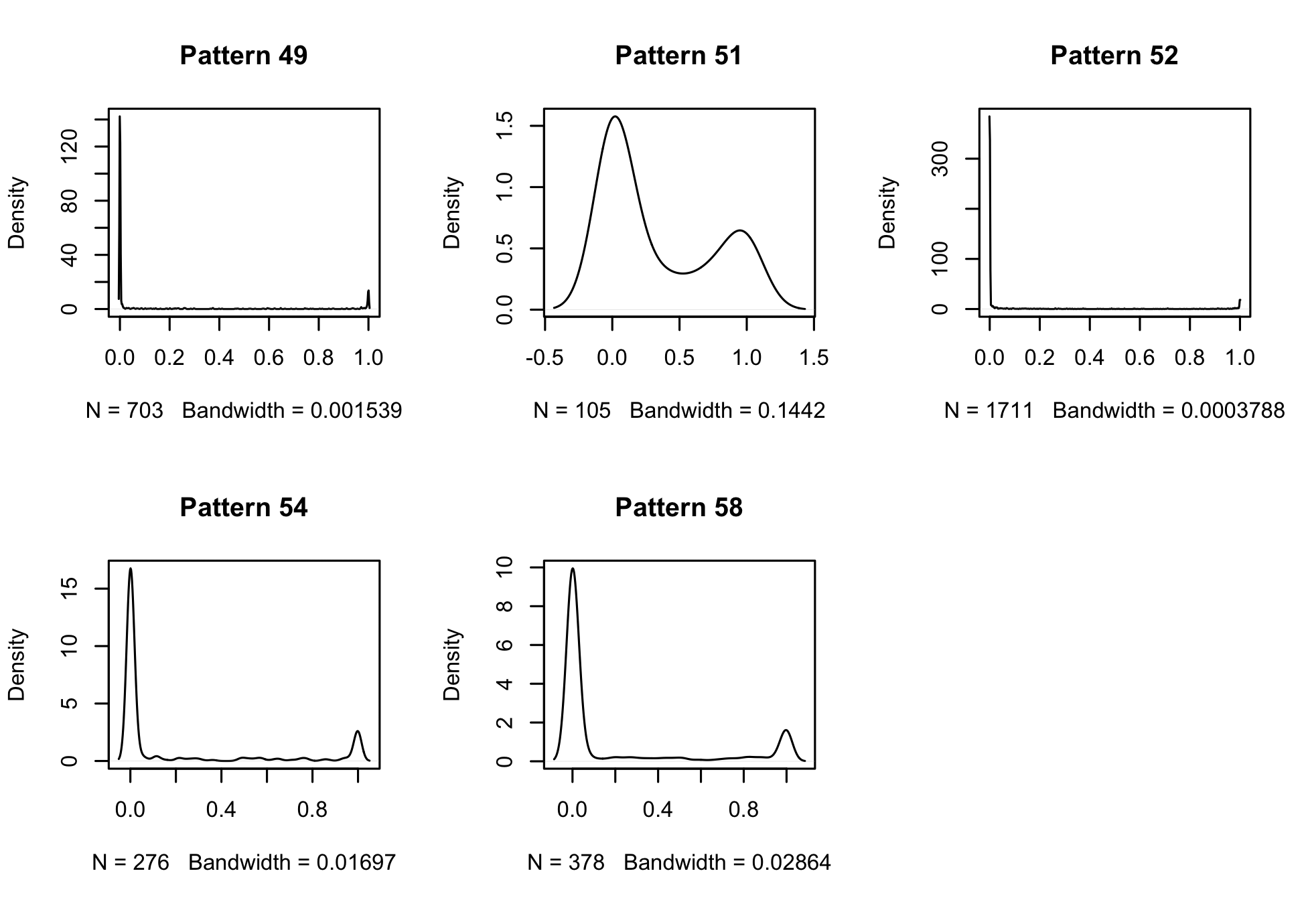

Supplement: Figure S4 — Density of the distribution of the edge recovery proportion from bootstraps for all possible node pairs. Patterns 49 to 58. (TIFF) [file pone.0050888.s004.tiff]
