# Supplementary material for: Graphical Modeling of Gene Expression in Monocytes Suggests Molecular Mechanisms Explaining Increased Atherosclerosis in Smokers
Source: PLoS One. 2013 Jan 23;8(1):e50888. doi: 10.1371/journal.pone.0050888 (PMC3553098; doi:10.1371/journal.pone.0050888)
Supplement: Table S1 — Risk factors associated to plaques count, selected by stepwise negative binomial regression. (DOC) [file pone.0050888.s005.doc]

| **Table S1.** Risk factors associated to plaques count, selected by stepwise negative binomial regression analysis. | | | | | | |
| --- | --- | --- | --- | --- | --- | --- |
| **Coefficient** | **beta** | **S.E.** | **CI95** | **z** | **P-value** | **r^2^_D_** |
| (Intercept) | -9.39 | 0.95 | (-11.3;-7.57) | -9.90 | 4.11E-23 |  |
| age | 0.10 | 0.01 | (0.08;0.11) | 14.39 | 6.15E-47 | 0.26 |
| sex | 0.39 | 0.13 | (0.14;0.64) | 3.03 | 0.0024 | 0.01 |
| HDL_CHOL | 0.04 | 0.01 | (0.01;0.07) | 2.94 | 3.30E-03 | 0.01 |
| LDL_CHOL | 0.02 | 0.01 | (0.01;0.03) | 4.28 | 1.84E-05 | 0.01 |
| diabetes | 0.55 | 0.20 | (0.17;0.94) | 2.82 | 0.0048 | 0.01 |
| smoking | 0.99 | 0.14 | (0.71;1.27) | 7.08 | 1.44E-12 | 0.07 |
| HDL_CHOL:LDL_CHOL | -4E-4 | 9E-5 | (-6E-4;-2E-4) | -3.90 | 9.48E-05 | 0.02 |
| Estimated θ parameter for negative binomial=0.89 (±0.12). **r^2^_D_** for HDL_CHOL and LDL_CHOL were estimated from a reduced model without interaction. | | | | | | |
