# Supplementary material for: Graphical Modeling of Gene Expression in Monocytes Suggests Molecular Mechanisms Explaining Increased Atherosclerosis in Smokers
Source: PLoS One. 2013 Jan 23;8(1):e50888. doi: 10.1371/journal.pone.0050888 (PMC3553098; doi:10.1371/journal.pone.0050888)
Supplement: Table S3 — Top 20 genes explaining covariation between smoking and plaques. (DOC) [file pone.0050888.s007.doc]

| **Table S3.** Top 20 genes explaining covariation between smoking and plaques. | | | | | | | | | |
| --- | --- | --- | --- | --- | --- | --- | --- | --- | --- |
| **ProbeID** | **Gene Symbol** | **Rank of association** | | | | **P~S|G** | | **P~G|S** | |
|  |  | **G~S** | **G~S\|P** | **G~P** | **G~P\|S** | **r^2^_D_** | **p-value** | **r^2^_D_** | **p-value** |
| ILMN_2185984 | SASH1 | 1 | 1 | 3 | 283 | 0.032 | 1.49E-06 | 0.020 | 0.0002 |
| ILMN_1664464 | PTGDS | 7 | 7 | 5 | 150 | 0.048 | 2.38E-09 | 0.019 | 0.0002 |
| ILMN_1800225 | PPARG | 48 | 69 | 1 | 1 | 0.052 | 4.10E-10 | 0.022 | 0.0001 |
| ILMN_1717207 | MMP25 | 4 | 4 | 36 | 5038 | 0.055 | 1.04E-10 | 0.005 | 0.0606 |
| ILMN_2103107 | ADAMDEC1 | 52 | 68 | 4 | 10 | 0.057 | 8.61E-11 | 0.033 | 0.0000 |
| ILMN_1818677 | LOC157627 | 11 | 14 | 32 | 1692 | 0.055 | 8.59E-11 | 0.009 | 0.0137 |
| ILMN_1752728 | FUCA1 | 5 | 5 | 96 | 7768 | 0.058 | 2.36E-11 | 0.007 | 0.0256 |
| ILMN_1654398 | RGL1 | 12 | 11 | 1128 | 22839 | 0.064 | 1.85E-12 | 0.005 | 0.0548 |
| ILMN_2352633 | ARHGAP24 | 606 | 1081 | 7 | 4 | 0.065 | 1.32E-12 | 0.021 | 0.0001 |
| ILMN_1664978 | TJP2 | 64 | 76 | 35 | 401 | 0.065 | 1.26E-12 | 0.011 | 0.0054 |
| ILMN_1796094 | CD36 | 86 | 112 | 19 | 117 | 0.066 | 7.15E-13 | 0.008 | 0.0178 |
| ILMN_1693338 | CYP1B1 | 136 | 155 | 306 | 2270 | 0.066 | 6.91E-13 | 0.004 | 0.0961 |
| ILMN_2102330 | COL8A2 | 47 | 53 | 27 | 341 | 0.067 | 4.82E-13 | 0.009 | 0.0130 |
| ILMN_1728478 | CXCL16 | 36 | 37 | 198 | 3384 | 0.067 | 4.33E-13 | 0.005 | 0.0602 |
| ILMN_1656951 | APCDD1 | 55 | 55 | 199 | 2640 | 0.068 | 3.03E-13 | 0.004 | 0.0994 |
| ILMN_1735495 | TBC1D8 | 21 | 22 | 304 | 6968 | 0.068 | 3.01E-13 | 0.003 | 0.1528 |
| ILMN_1763207 | BATF3 | 30 | 31 | 26 | 536 | 0.068 | 2.59E-13 | 0.005 | 0.0759 |
| ILMN_1656300 | GFRA2 | 8 | 8 | 959 | 22471 | 0.068 | 2.38E-13 | 0.000 | 0.6991 |
| ILMN_1776121 | C2orf55 | 24 | 25 | 246 | 5376 | 0.068 | 2.25E-13 | 0.002 | 0.2445 |
| ILMN_1667966 | FAM129A | 23 | 26 | 25 | 586 | 0.069 | 2.05E-13 | 0.016 | 0.0010 |
| G: gene expression, S: smoking, P: plaques. Association of expression with smoking and/or plaques was tested by linear models 1 (G~S), 2 (G~P), and 3 (G~P|S and G~S|P). The marginal effects of smoking and gene expression on plaques once conditioned on each other (P~S|G and P~G|P, respectively) were tested in a negative binomial model. Genes are ranked by increasing r^2^_D_ associated to S in the model P~S|G, which corresponds to decreasing reduction of the amount of covariation between smoking and plaques explained by gene expression. | | | | | | | | | |
