# Supplementary material for: Graphical Modeling of Gene Expression in Monocytes Suggests Molecular Mechanisms Explaining Increased Atherosclerosis in Smokers
Source: PLoS One. 2013 Jan 23;8(1):e50888. doi: 10.1371/journal.pone.0050888 (PMC3553098; doi:10.1371/journal.pone.0050888)
Supplement: Table S5 — Gene Ontology categories enriched for gene expressions in specific ICA modules. (DOC) [file pone.0050888.s009.doc]

| **Table S5.** Gene Ontology categories enriched for gene expressions in specific ICA modules. | | | | | |
| --- | --- | --- | --- | --- | --- |
| **GO** | **Term** | **Genes in GO class** | **Genes in Module** | **p-value** | **Bonferroni corrected*** |
| ***Module 18*** | | | | | |
| BP | interferon-gamma-mediated signaling pathway | 65 | 12 | 1.8E-13 | 2.6E-8 |
| CC | MHC class II protein complex | 12 | 4 | 3.5E-7 | 0.0071 |
| ***Module 39*** | | | | | |
| BP | antigen processing and presentation of peptide or polysaccharide antigen via MHC class II | 16 | 4 | 1.4E-7 | 0.0203 |
| CC | MHC class II protein complex | 12 | 4 | 2.4E-8 | 0.0005 |
| MF | MHC class II receptor activity | 8 | 3 | 1.2E-6 | 0.0449 |
| * Bonferroni correction on the number of GO terms represented in the reference set: 5010 (BP), 700 (CC), 1289 (MF). | | | | | |
