# Supplementary material for: Graphical Modeling of Gene Expression in Monocytes Suggests Molecular Mechanisms Explaining Increased Atherosclerosis in Smokers
Source: PLoS One. 2013 Jan 23;8(1):e50888. doi: 10.1371/journal.pone.0050888 (PMC3553098; doi:10.1371/journal.pone.0050888)
Supplement: Table S6 — Enrichment of ICA module gene sets in monocytes for genes associated to smoking in lymphocytes [Charlesworth et al. 2010]. (DOC) [file pone.0050888.s010.doc]

| **Table S6.** Enrichment of ICA module gene sets in monocytes for genes associated to smoking in lymphocytes [Charlesworth *et al.* 2010]. | | | | | |
| --- | --- | --- | --- | --- | --- |
| **Pattern** | **N** | **Observed** | **Expected** | **OR** | **P-value** |
| Pattern4 | 111 | 3 | 2.4 | 1.27 | 0.3699 |
| Pattern11 | 129 | 36 | 2.7 | 18.05 | **6.6E-32** |
| Pattern12 | 98 | 5 | 2 | 2.64 | 0.0431 |
| Pattern14 | 59 | 1 | 1.2 | 0.82 | 0.6889 |
| Pattern15 | 14 | 0 | 0.3 | 0.00 | 1.0000 |
| Pattern17 | 27 | 1 | 0.6 | 1.79 | 0.4135 |
| Pattern18 | 92 | 7 | 1.8 | 4.03 | 0.0022 |
| Pattern19 | 96 | 2 | 2 | 0.99 | 0.5630 |
| Pattern21 | 32 | 2 | 0.7 | 3.13 | 0.1290 |
| Pattern23 | 38 | 0 | 0.8 | 0.00 | 1.0000 |
| Pattern27 | 12 | 1 | 0.2 | 4.56 | 0.2110 |
| Pattern28 | 60 | 1 | 1.2 | 0.82 | 0.6950 |
| Pattern29 | 94 | 9 | 1.9 | 5.25 | **9.1E-05** |
| Pattern30 | 71 | 5 | 1.5 | 3.53 | 0.0126 |
| Pattern31 | 81 | 8 | 1.6 | 5.42 | **0.0002** |
| Pattern33 | 42 | 3 | 0.8 | 3.76 | 0.0485 |
| Pattern34 | 28 | 1 | 0.7 | 1.43 | 0.4250 |
| Pattern36 | 56 | 3 | 1.1 | 2.74 | 0.0964 |
| Pattern39 | 46 | 9 | 0.9 | 12.20 | **2.1E-07** |
| Pattern41 | 39 | 0 | 0.8 | 0.00 | 1.0000 |
| Pattern42 | 67 | 4 | 1.4 | 3.04 | 0.0420 |
| Pattern43 | 34 | 12 | 0.7 | 27.35 | **9.1E-13** |
| Pattern45 | 45 | 1 | 0.9 | 1.09 | 0.5893 |
| Pattern48 | 22 | 1 | 0.4 | 2.39 | 0.3526 |
| Pattern49 | 34 | 4 | 0.7 | 6.69 | 0.0042 |
| Pattern51 | 8 | 1 | 0.2 | 7.16 | 0.1462 |
| Pattern52 | 46 | 9 | 0.9 | 11.88 | **2.1E-07** |
| Pattern54 | 16 | 1 | 0.3 | 3.34 | 0.2710 |
| Pattern58 | 21 | 3 | 0.4 | 7.92 | 0.0076 |
| N is the number of genes in a module that are present in both studies. P-values exceeding Bonferroni correction threshold 0.05/29 = 0.0017 are in bold. | | | | | |
