# Supplementary material for: Graphical Modeling of Gene Expression in Monocytes Suggests Molecular Mechanisms Explaining Increased Atherosclerosis in Smokers
Source: PLoS One. 2013 Jan 23;8(1):e50888. doi: 10.1371/journal.pone.0050888 (PMC3553098; doi:10.1371/journal.pone.0050888)
Supplement: Table S7 — Cardiovascular risk factors associated to expression patterns by stepwise regression. (DOC) [file pone.0050888.s011.doc]

| **Table S7.** Risk factors associated to patterns by stepwise regression. | |
| --- | --- |
| **Pattern** | **Risk factors** |
| Pattern4 | bmi |
| Pattern11 | age + bmi |
| Pattern12 | HDL_CHOL |
| Pattern14 | age |
| Pattern15 | age |
| Pattern17 | MPO |
| Pattern18 | age + bmi + MPO |
| Pattern19 | sbp |
| Pattern21 | age + sex + HDL_CHOL + diabetes + CRP + MPO |
| Pattern23 | sex |
| Pattern27 | age |
| Pattern28 | age + CRP + MPO |
| Pattern29 | age + sex + bmi + sbp + TRIGLY |
| Pattern30 | CRP |
| Pattern31 | age + HDL_CHOL + CRP + MPO |
| Pattern33 | age |
| Pattern34 | CRP |
| Pattern36 | CRP |
| Pattern39 | age |
| Pattern41 | LDL_CHOL |
| Pattern42 | bmi + sbp + diabetes |
| Pattern43 | age + bmi + HDL_CHOL + HCY |
| Pattern45 | sex + sbp |
| Pattern48 | sex + LDL_CHOL + CRP |
| Pattern49 | age + sex + sbp + HDL_CHOL |
| Pattern51 | age + sex + sbp + CRP |
| Pattern52 | age + HDL_CHOL + CRP + MPO |
| Pattern54 | sex + bmi + TRIGLY + CRP |
| Pattern58 | HDL_CHOL |
