# Supplementary material for: Graphical Modeling of Gene Expression in Monocytes Suggests Molecular Mechanisms Explaining Increased Atherosclerosis in Smokers
Source: PLoS One. 2013 Jan 23;8(1):e50888. doi: 10.1371/journal.pone.0050888 (PMC3553098; doi:10.1371/journal.pone.0050888)
Supplement: Table S9 — Quality control statistics for ICA components of variance. (DOC) [file pone.0050888.s013.doc]

| **Table S9.** Quality control statistics for ICA components of variance. | | | | | | | | | | |
| --- | --- | --- | --- | --- | --- | --- | --- | --- | --- | --- |
| **Comp** | **Kur** | **Var (%)** | **Negen (%)** | **Module Stab** | **Signature** | | **Pattern** | | **Outlier var** | |
|  |  |  |  |  | **Stab** | **>0.95** | **Stab** | **>0.95** | **%** | **<10%** |
| 1 | 7.1 | 6.5 | 0.8 | 0.96 | 1 | x | 1 | x | 0.59 | x |
| 2 | 11 | 6.4 | 2.6 | 0.87 | 0.98 | x | 1 | x | 2.6 | x |
| 3 | 4.9 | 5.8 | 0.29 | 0.61 | 0.96 | x | 0.97 | x | 0.97 | x |
| 4 | 12 | 5.6 | 2.3 | 0.92 | 1 | x | 1 | x | 1.1 | x |
| 5 | 8.3 | 5.4 | 1.2 | 0.94 | 1 | x | 1 | x | 0.72 | x |
| 6 | 5.3 | 3.8 | 0.37 | 0.72 | 0.97 | x | 0.97 | x | 2.7 | x |
| 7 | 4.3 | 3.5 | 0.16 | 0.25 | 0.92 |  | 0.94 |  | 3.9 | x |
| 8 | 6.3 | 3.5 | 0.47 | 0.87 | 1 | x | 1 | x | 2.2 | x |
| 9 | 9.4 | 3.2 | 1.2 | 0.95 | 1 | x | 1 | x | 1.1 | x |
| 10 | 7.3 | 3.1 | 0.66 | 0.8 | 0.96 | x | 0.94 |  | 3.2 | x |
| 11 | 10 | 2.9 | 1.7 | 0.95 | 1 | x | 1 | x | 2.2 | x |
| 12 | 9.5 | 2.6 | 1.3 | 0.95 | 1 | x | 1 | x | 1.4 | x |
| 13 | 5 | 2.5 | 0.32 | 0.74 | 0.98 | x | 0.99 | x | 32 |  |
| 14 | 11 | 2.4 | 1.3 | 0.96 | 1 | x | 1 | x | 1.6 | x |
| 15 | 4.7 | 2.2 | 0.19 | 0.66 | 0.96 | x | 0.97 | x | 2.4 | x |
| 16 | 26 | 2.2 | 4.1 | 0.62 | 0.92 |  | 0.99 | x | 2.5 | x |
| 17 | 5.3 | 2.1 | 0.36 | 0.59 | 0.97 | x | 0.96 | x | 1.1 | x |
| 18 | 15 | 1.9 | 2.1 | 0.93 | 1 | x | 1 | x | 4.9 | x |
| 19 | 26 | 1.9 | 4.7 | 0.95 | 1 | x | 1 | x | 0.92 | x |
| 20 | 4.7 | 1.6 | 0.2 | 0.46 | 0.9 |  | 0.92 |  | 2.5 | x |
| 21 | 6.6 | 1.6 | 0.5 | 0.61 | 0.97 | x | 0.99 | x | 2.6 | x |
| 22 | 14 | 1.5 | 2 | 0.5 | 0.9 |  | 0.51 |  | 1.7 | x |
| 23 | 11 | 1.5 | 0.99 | 0.92 | 1 | x | 1 | x | 1.2 | x |
| 24 | 48 | 1.4 | 7.3 | 0.97 | 1 | x | 1 | x | 3 | x |
| 25 | 28 | 1.3 | 5.4 | 0.99 | 1 | x | 1 | x | 18 |  |
| 26 | 23 | 1.2 | 2.1 | 0.98 | 1 | x | 1 | x | 10 |  |
| 27 | 6.9 | 1.1 | 0.38 | 0.76 | 0.96 | x | 0.96 | x | 2.2 | x |
| 28 | 8.2 | 1.1 | 0.74 | 0.87 | 1 | x | 1 | x | 3.4 | x |
| 29 | 12 | 1.1 | 1.7 | 0.88 | 1 | x | 1 | x | 1.6 | x |
| 30 | 14 | 1 | 2.1 | 0.83 | 0.99 | x | 1 | x | 3.1 | x |
| 31 | 45 | 0.97 | 6.9 | 0.98 | 1 | x | 1 | x | 1.8 | x |
| 32 | 55 | 0.94 | 8 | 0.97 | 1 | x | 1 | x | 6.2 | x |
| 33 | 10 | 0.92 | 0.87 | 0.92 | 1 | x | 1 | x | 0.59 | x |
| 34 | 16 | 0.87 | 1.8 | 0.84 | 0.97 | x | 0.98 | x | 0.85 | x |
| 35 | 4.6 | 0.86 | 0.15 | 0.39 | 0.87 |  | 0.82 |  | 2.2 | x |
| 36 | 21 | 0.86 | 2 | 0.96 | 1 | x | 1 | x | 1.7 | x |
| 37 | 5.8 | 0.76 | 0.35 | 0.32 | 0.8 |  | 0.83 |  | 2.5 | x |
| 38 | 61 | 0.73 | 6.6 | 0.97 | 1 | x | 1 | x | 33 |  |
| 39 | 7.6 | 0.73 | 0.72 | 0.73 | 0.98 | x | 0.98 | x | 1.7 | x |
| 40 | 11 | 0.73 | 1.1 | 0.94 | 1 | x | 1 | x | 68 |  |
| 41 | 9.6 | 0.7 | 0.84 | 0.92 | 1 | x | 0.99 | x | 3 | x |
| 42 | 22 | 0.65 | 2.4 | 0.94 | 1 | x | 1 | x | 9.9 | x |
| 43 | 17 | 0.63 | 1.5 | 0.82 | 1 | x | 1 | x | 2.5 | x |
| 44 | 4.1 | 0.6 | 0.092 | 0.36 | 0.86 |  | 0.82 |  | 3.2 | x |
| 45 | 23 | 0.6 | 1.9 | 0.92 | 1 | x | 1 | x | 3.2 | x |
| 46 | 4.1 | 0.56 | 0.11 | 0 | 0.94 |  | 0.93 |  | 9 | x |
| 47 | 5.6 | 0.55 | 0.25 | 0.37 | 0.82 |  | 0.8 |  | 2 | x |
| 48 | 30 | 0.54 | 1.2 | 0.93 | 1 | x | 1 | x | 0.68 | x |
| 49 | 26 | 0.53 | 2 | 0.85 | 1 | x | 1 | x | 2.2 | x |
| 50 | 10 | 0.51 | 0.52 | 0.91 | 1 | x | 0.99 | x | 10 |  |
| 51 | 7 | 0.5 | 0.31 | 0.67 | 0.96 | x | 0.96 | x | 2.2 | x |
| 52 | 14 | 0.5 | 1.1 | 0.84 | 1 | x | 1 | x | 1.4 | x |
| 53 | 6.7 | 0.45 | 0.34 | 0.78 | 0.88 |  | 0.84 |  | 35 |  |
| 54 | 100 | 0.45 | 6 | 1 | 1 | x | 1 | x | 4.9 | x |
| 55 | 9.4 | 0.43 | 0.74 | 0.87 | 1 | x | 0.99 | x | 61 |  |
| 56 | 4.4 | 0.41 | 0.12 | 0.67 | 0.97 | x | 0.95 |  | 5.2 | x |
| 57 | 5.7 | 0.4 | 0.34 | 0.39 | 0.83 |  | 0.81 |  | 17 |  |
| 58 | 35 | 0.39 | 2.1 | 0.87 | 1 | x | 1 | x | 5.8 | x |
| 59 | 4.6 | 0.38 | 0.13 | 0.84 | 0.95 |  | 0.92 |  | 2.2 | x |
| Comp: ICA component, Kur: kurtosis, Var: variance, Negen: negentropy, Stab: stability, Maxvar Indiv: maximum of the pattern variance (%) explained by a single individual. The variance explained by a component (%) is relative to the total variance explained by ICA components, i.e. 65% of variance of expression data after normalization and scaling. The negentropy of a component (%) is relative to the total negentropy in the best ICA run (0.345).  From the 59 components, 29 were discarded because they had a signature or a pattern stability ≤ 0.95 across 1000 bootstraps, or they were attributable to a single individual explaining ≥ 10% of the variance. | | | | | | | | | | |
