# Supplementary material for: Graphical Modeling of Gene Expression in Monocytes Suggests Molecular Mechanisms Explaining Increased Atherosclerosis in Smokers
Source: PLoS One. 2013 Jan 23;8(1):e50888. doi: 10.1371/journal.pone.0050888 (PMC3553098; doi:10.1371/journal.pone.0050888)
Supplement: Table S10 — Pearson correlation coefficients between ICA patterns and 6 first SVD components of expression matrix. (DOC) [file pone.0050888.s014.doc]

| **Table S10.** Pearson correlation coefficient between ICA patterns and 6 first SVD components of expression matrix. | | | | | | |
| --- | --- | --- | --- | --- | --- | --- |
| **Pattern** | **SV1** | **SV2** | **SV3** | **SV4** | **SV5** | **SV6** |
| Pattern1 | -0.59 | **0.70** | 0.11 | 0.20 | -0.10 | 0.07 |
| Pattern2 | 0.03 | -0.11 | -0.08 | -0.02 | -0.16 | 0.34 |
| Pattern3 | **-0.83** | 0.19 | 0.36 | 0.05 | 0.10 | 0.08 |
| Pattern4 | 0.63 | 0.34 | 0.48 | 0.11 | 0.16 | -0.37 |
| Pattern5 | -0.08 | **0.76** | -0.43 | 0.39 | 0.01 | -0.03 |
| Pattern6 | -0.09 | **-0.71** | -0.21 | 0.41 | 0.05 | -0.21 |
| Pattern8 | 0.04 | 0.10 | -0.19 | -0.31 | **0.78** | 0.31 |
| Pattern9 | **-0.84** | -0.01 | 0.32 | 0.01 | -0.11 | 0.03 |
| Pattern11 | 0.12 | -0.04 | -0.04 | -0.02 | -0.09 | 0.10 |
| Pattern12 | -0.37 | -0.39 | 0.67 | -0.16 | -0.08 | 0.00 |
| Pattern14 | 0.02 | 0.56 | 0.01 | -0.51 | 0.32 | -0.06 |
| Pattern15 | -0.22 | -0.25 | -0.44 | -0.44 | -0.37 | 0.07 |
| Pattern17 | -0.22 | 0.01 | -0.28 | 0.32 | -0.06 | 0.07 |
| Pattern18 | -0.13 | -0.07 | 0.00 | 0.09 | -0.06 | -0.10 |
| Pattern19 | -0.23 | 0.39 | 0.31 | -0.19 | -0.08 | -0.11 |
| Pattern21 | 0.07 | -0.08 | -0.08 | 0.06 | -0.14 | 0.17 |
| Pattern23 | 0.11 | -0.55 | 0.15 | 0.47 | -0.09 | 0.02 |
| Pattern24 | 0.08 | 0.13 | 0.01 | 0.06 | -0.02 | 0.03 |
| Pattern27 | 0.17 | 0.41 | -0.32 | -0.09 | 0.35 | 0.19 |
| Pattern28 | 0.15 | 0.22 | -0.04 | -0.25 | 0.35 | -0.02 |
| Pattern29 | 0.09 | 0.07 | 0.00 | 0.05 | -0.04 | -0.09 |
| Pattern30 | -0.54 | -0.18 | -0.38 | -0.27 | 0.02 | -0.42 |
| Pattern31 | 0.00 | 0.00 | 0.10 | -0.07 | 0.07 | -0.13 |
| Pattern32 | -0.11 | 0.11 | 0.04 | 0.01 | 0.07 | 0.06 |
| Pattern33 | 0.09 | -0.21 | -0.16 | 0.27 | -0.43 | -0.07 |
| Pattern34 | 0.00 | 0.00 | -0.07 | 0.22 | 0.56 | -0.02 |
| Pattern36 | 0.28 | 0.15 | -0.10 | -0.17 | -0.18 | 0.09 |
| Pattern39 | -0.06 | -0.15 | -0.03 | 0.03 | 0.00 | -0.04 |
| Pattern41 | 0.02 | 0.07 | -0.16 | 0.00 | -0.11 | 0.57 |
| Pattern42 | -0.11 | -0.11 | 0.09 | 0.02 | -0.05 | 0.04 |
| Pattern43 | -0.09 | 0.06 | -0.01 | 0.08 | 0.01 | 0.11 |
| Pattern45 | 0.12 | 0.07 | 0.08 | -0.06 | 0.06 | -0.08 |
| Pattern48 | 0.00 | -0.04 | -0.04 | -0.02 | 0.03 | -0.12 |
| Pattern49 | -0.19 | -0.01 | -0.06 | 0.10 | 0.07 | 0.05 |
| Pattern51 | -0.03 | 0.07 | -0.03 | 0.02 | -0.15 | 0.14 |
| Pattern52 | 0.05 | -0.09 | -0.14 | -0.05 | 0.06 | -0.03 |
| Pattern54 | 0.00 | -0.04 | -0.06 | 0.03 | -0.12 | 0.11 |
| Pattern58 | 0.05 | -0.25 | 0.14 | 0.00 | -0.11 | -0.15 |
| SV1 to SV6 are the 6 first variance components from singular value decomposition (SVD) of the expression matrix. Since none of them was associated to any measured risk factor, they were assumed to reflect technical sources of variability in expression data. The patterns showing an absolute correlation ≥ 0.7 with any of the 6 SVD components (in bold) were discarded (n = 6). | | | | | | |
