# Supplementary material for: Graphical Modeling of Gene Expression in Monocytes Suggests Molecular Mechanisms Explaining Increased Atherosclerosis in Smokers
Source: PLoS One. 2013 Jan 23;8(1):e50888. doi: 10.1371/journal.pone.0050888 (PMC3553098; doi:10.1371/journal.pone.0050888)
Supplement: Table S11 — Pearson correlation coefficients between ICA patterns and surrogate variables for cell contamination by non-monocytic cells. (DOC) [file pone.0050888.s015.doc]

| **Table S11.** Pearson correlation coefficient between ICA patterns and surrogate variables for cell contamination by non-monocytic cells. | | | |
| --- | --- | --- | --- |
| **Pattern** | **B cells** | **T cells** | **MK** |
| Pattern1 | 0.17 | -0.08 | -0.05 |
| Pattern2 | 0.04 | 0.08 | **-0.77** |
| Pattern3 | 0.35 | -0.10 | -0.01 |
| Pattern4 | -0.15 | 0.08 | -0.05 |
| Pattern5 | -0.09 | 0.05 | 0.03 |
| Pattern6 | -0.10 | 0.02 | -0.06 |
| Pattern8 | 0.12 | -0.05 | -0.01 |
| Pattern9 | 0.29 | -0.08 | -0.01 |
| Pattern11 | -0.03 | 0.21 | 0.04 |
| Pattern12 | 0.17 | -0.10 | 0.03 |
| Pattern14 | 0.03 | -0.03 | 0.00 |
| Pattern15 | 0.01 | -0.04 | 0.07 |
| Pattern17 | -0.04 | 0.00 | 0.10 |
| Pattern18 | 0.02 | -0.08 | 0.08 |
| Pattern19 | 0.06 | 0.00 | -0.18 |
| Pattern21 | 0.14 | 0.26 | -0.05 |
| Pattern23 | 0.00 | 0.09 | -0.01 |
| Pattern24 | 0.18 | **0.91** | -0.27 |
| Pattern27 | -0.04 | -0.04 | -0.03 |
| Pattern28 | 0.06 | -0.13 | 0.05 |
| Pattern29 | -0.07 | -0.09 | 0.09 |
| Pattern30 | 0.15 | -0.06 | 0.04 |
| Pattern31 | -0.19 | -0.15 | 0.06 |
| Pattern32 | **0.82** | 0.08 | -0.25 |
| Pattern33 | -0.09 | 0.03 | 0.10 |
| Pattern34 | 0.03 | -0.01 | -0.04 |
| Pattern36 | -0.09 | 0.01 | 0.18 |
| Pattern39 | -0.16 | -0.19 | -0.09 |
| Pattern41 | 0.02 | 0.03 | -0.03 |
| Pattern42 | 0.12 | 0.03 | -0.01 |
| Pattern43 | 0.20 | -0.03 | -0.26 |
| Pattern45 | -0.14 | -0.10 | 0.16 |
| Pattern48 | -0.16 | -0.26 | 0.29 |
| Pattern49 | 0.13 | -0.04 | -0.08 |
| Pattern51 | 0.15 | 0.21 | 0.08 |
| Pattern52 | 0.06 | -0.07 | -0.01 |
| Pattern54 | -0.16 | -0.13 | 0.01 |
| Pattern58 | 0.09 | 0.00 | 0.01 |
| Contamination of samples by non-monocytic RNA was estimated from the expression of genes that are considered to be cell-type specific (Text S1). MK: megakaryocytes. The patterns showing an absolute correlation ≥ 0.7 with any of the 3 cell types (in bold) were discarded (n = 3). | | | |
