# Supplementary material for: Graphical Modeling of Gene Expression in Monocytes Suggests Molecular Mechanisms Explaining Increased Atherosclerosis in Smokers
Source: PLoS One. 2013 Jan 23;8(1):e50888. doi: 10.1371/journal.pone.0050888 (PMC3553098; doi:10.1371/journal.pone.0050888)
Supplement: Text S1 — Supporting information: further details of Methods. (DOC) [file pone.0050888.s016.doc]

Supplementary Text S1

*Graphical models of smoking and plaques gene networks*

Verdugo *et al.*

# Modeling plaques


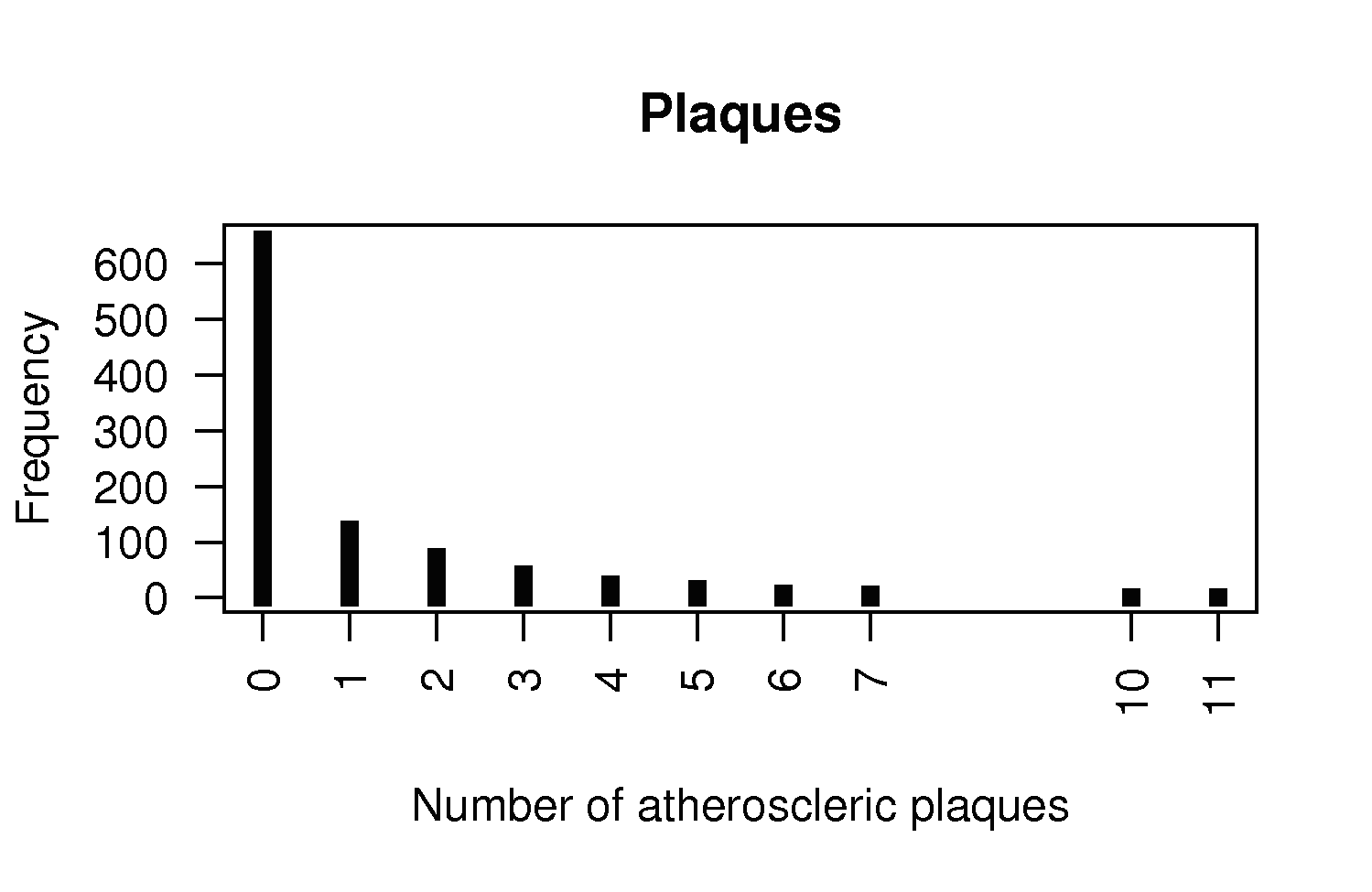
Figure A1. Distribution of count of atherosclerotic plaques in GHS.

For modeling the discrete phenotype "count of plaques", four distribution functions were compared: Poisson, Negative Binomial (Negbin-2), Hurdle, and Zero-Inflated. The Poisson model was fitted by the *glm* function in R. The Negative Binomial was fit by the *glm.nb* function of the MASS package in R. The Hurdle and Zero-Inflated (ZI) models were fit with the hurdle and *zeroinfl* of the *pscl* package in R. Models were compared in terms of likelihood of the data given the model, model complexity, and fitted number of zero values. The Poisson model had the lowest likelihood, clear overdispersion evidenced by a deviance much larger than the residual degrees of freedom (Table A1). This was not corrected by adding an interaction term between sex and smoking (PoisInt model). The negative binomial distribution showed better fit while using fewer degrees of freedom than the more complex models Hurdle and ZI. Its residual deviance was much lower than the degrees of freedom, indicating that the extra parameter properly accounted for the observed overdispersion. Therefore, the negative binomial distribution was chosen for modeling count of plaques in all analyses.

| **Table A1.** Comparison of plaques fit by alternative distribution functions (covariables: age, sex and smoking) | | | | | |
| --- | --- | --- | --- | --- | --- |
|  | **Poisson** | **PoisInt** | **NB** | **Hurdle** | **ZI** |
| logLik | -1034 | -1031 | -928 | -913 | -913 |
| Deviance | 1317 | 1312 | 685 |  |  |
| No.param | 4 | 5 | 5 | 9 | 9 |
| D.f. | 932 | 931 | 932 | 927 | 927 |
| BIC | 2094 | 2096 | 1890 | 1888 | 1887 |
| EZ | 541 | 540 | 634 | 644 | 643 |
| There were 644 zeros observed. PoisInt: Poisson with sex-by-smoking interaction, NB:Negative binomial, ZI: Zero-Inflated, No.param: number of parameters, EZ: expected zeros. | | | | | |

# Differential gene expression testing

Statistical association between gene expression, smoking and plaques was tested by linear models:

*g_i_ =ß_0_ + ∑_h_ ß_h_v_hi_ + ß_7_a_i_ + ß_8_x_i_ + ß_9_s_i_ + e_i_*, (1)

*g_i_ =ß_0_ + ∑_h_ ß_h_v_hi_ + ß_7_a_i_ + ß_8_x_i_ +* *ß_10_*log(*p_i_*) *+ e_i_*, (2)

*g_i_ =ß_0_ + ∑_h_ ß_h_v_hi_ + ß_7_a_i_ + ß_8_x_i_ + ß_9_s_i_* *+ ß_10_*log(*p_i_*) *+ e_i_*, (3)

log(*p_i_*) *=γ_0_ +* *γ_7_a_i_ + γ_8_x_i_ + γ_9_s_i_ + γ_10_g_i_ + e_i_*, (4)

where *g_i_* is the normalized intensity for one given probe in individual *i*, *v_hi_* (*h*=1 to 6) are surrogate variables for technical effects on expression measurement, *a_i_* is age, *x_i_* is sex, *s_i_* is smoking, *p_i_* is the number of plaques, and *e*_i_ is a residual. All models were tested by the *glm* function in *R* v2.13 [1]. Models 1-3 were fitted a Gaussian family function and Model 4 a Negative Binomial (NB-2) with the *negative.binomial* family function in the MASS package in R. The dispersion parameter *θ* was held constant across genes to a value of 0.75, which was estimated with the *glm.nb* function on the reduced model without the gene effect. P-values were corrected for multiple comparisons by the FDR method [2].

# Quality control criteria for ICA components

The fastICA was repeated 500 times with 59 components and the best run (with the maximal negentropy) was selected. The Pearson correlation between each signature from the best run (column of S) and all signatures from all other runs was calculated. The same was done for patterns (columns of A). For each run, the signature (pattern, resp.) with the highest absolute correlation to that in the best run was regarded as estimating the same underlying signature (pattern, resp.). The average correlation between corresponding signatures (patterns) across runs was denoted as the signature (pattern, resp.) stability. A minimum stability for signatures and patterns of 0.95, in a scale from 0 to 1, was required. In addition, patterns with ≥10% of their variance explained by a single individual were removed. Thirty-eight of the 59 components passed these filtering steps (Table S9). We further excluded 6 components that were correlated (r ≥ 0.7) to one of the six SVD components reflecting potential experimental variability (Table S10).

Because the method of monocyte enrichment used in GHS did not yield a 100% purity and even modest heterogeneity of cell content may induce artifactual correlation among expressions, we estimated the degree of potential RNA contamination by non-monocytic cells as previously described [3]. Briefly, gene expression profiles for different blood cell types (B and T cells, neutrophils, erythroblasts, and megakaryocytes) were obtained from HaemAtlas [4]. Although megakaryocytes are not present in circulating blood, expression signatures from these cells can derive from their generated platelets. The average expression of genes specific to each cell type was used as a surrogate variable of the degree of contamination of the RNA sample by that cell type. Only B and T cells, and megakaryocytes were present in detectable amounts. We estimated the correlation of ICA patterns with each of these three surrogate variables and further excluded 3 components that were correlated to any one of them (Table S11).

# Equivalence classes of causality models

Model of causality considered in this manuscript were evaluated as Bayesian Networks (BNs). BNs are composed of a directed acyclic graph (DAG) and density functions describing associations among nodes, here {S, G, P} representing smoking, gene expression and plaques, respectively. The causal model of interest can be represented by S→G→P. In this manuscript, we refer to this particular graph as the causal model where G and P are descendents of S and P is descendent of G. DAGs encode a number of statements of statistical associations among variables that follow the directed Markov condition: each variable is independent of variables that are not parents nor descendents, conditional on its parents (for an introduction to the topic on causal inference, see Spirtes [5]). In the present graph, this property translates in the conditional independence P⊥S|G, that is, a gene whose expression can account for all association between plaques and smoking, making the two statistically independent once conditioning on gene expression. The causal model is not, however, the only model that satisfies this condition. The same is true for models *S←G←P* and *S**←G→P*. Because all three models encode the same set conditional independencies, they cannot be distinguished by likelihood-based statistics and they make a single *equivalence class* of models (for more on this see [6]). Similarly, a closed tripled were all three nodes are directly connected us consistent with six different causal models (Figure A2). Here we aimed at finding genes, or set of genes, that fitted any class that included the causal model. The model class that best fitted the data was chosen by maximum likelihood and the confidence in the chose model was evaluated by bootstrapping (see Materials and Methods in main text).


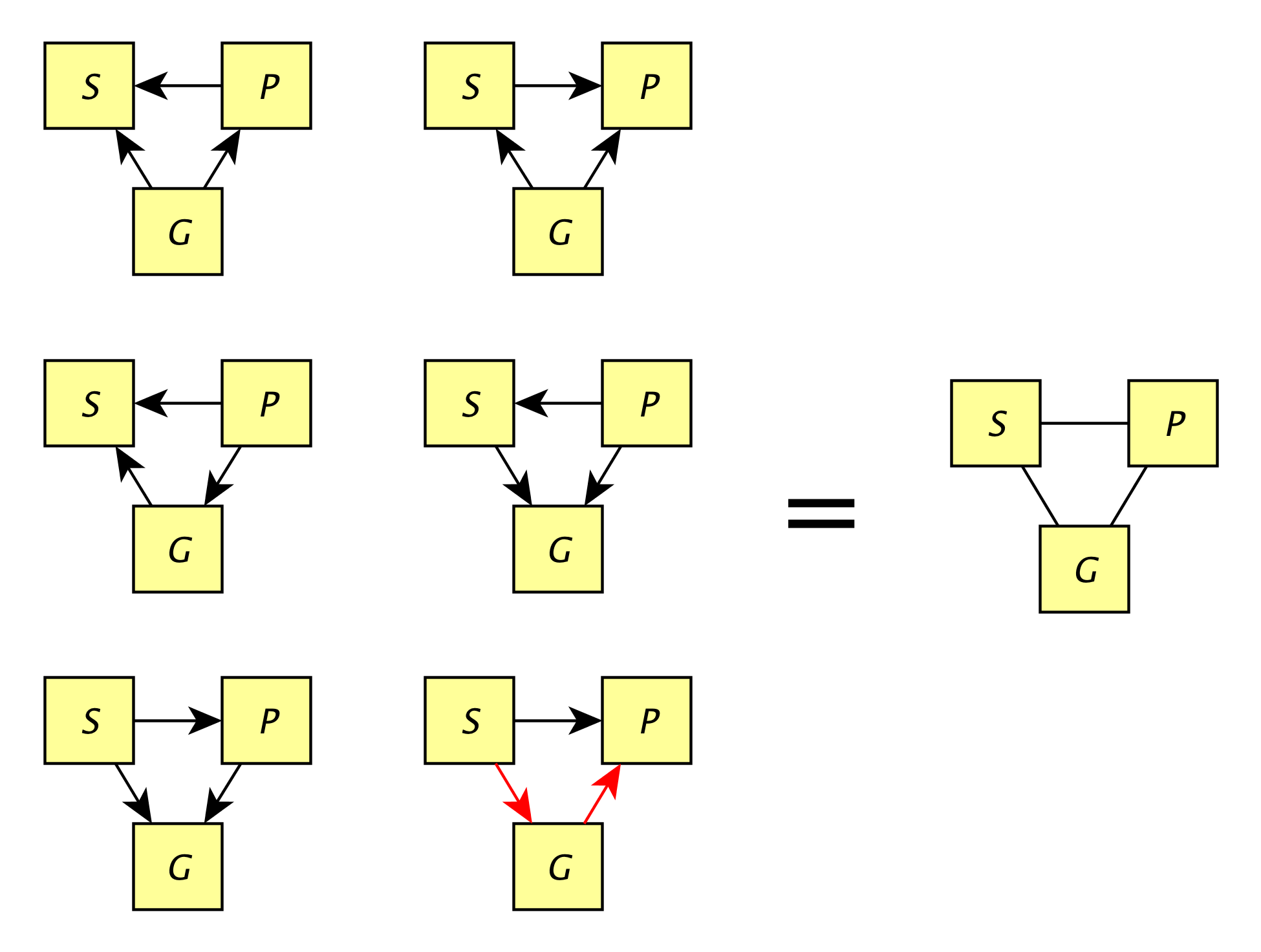


Figure A2. Causal DAGs for the closed triplet equivalent class of models. Edges of the causal path of interest *S* → *G* → *P* are in red.

# Causal models favored by *a priori* information

The causality test employed in this paper can only distinguish among equivalence classes of Bayesian networks, which may include multiple causal models. Because this is an observational study, we could not discard some causal models as done in experimental crosses, where causal effects from phenotypes or gene expression to genotypes can safely be ignored [7]. However, field knowledge may be used to favor one candidate network versus another. A Bayesian framework for network inference could accommodate these different sources of information as priors for the probability of certain association structures. Deciding how best to balance prior information with data is not, however, straightforward, and a wrong choice risks incorporating bias into the interference. We opted for selecting genes based on data-driven-only tests and then using *a priori* information for discerning among likely models for at the time of interpreting results.

We investigated the literature for genes with evidence of *G*→*S*. We collected a list of 156 genes with genetic variants previously associated to smoking phenotypes, primarily from GWAS studies (Table S8). We only observed three modules containing genes previously associated to smoking (patterns 4, 14, and 18; Table S4, spreadsheet “ICA phenos”)). This is in agreement with the fact that genes associated to smoking are mostly expressed in brain, not monocytes, and are associated to biological processes of the nervous system [8,9]. Therefore, for the expression of genes in monocytes, *a priori* we favor *S*→*G*.

Finally, information about the design of the study was used to favor one direction in *S–P* edges. An *S*←*P* effect may occur if, for instance, a person is diagnosed with atherosclerosis or cardiovascular disease and he (or she) decides to stop smoking, probably following a doctor’s advice. In the present study, the importance of this effect, however, was minimized because recent ex-smokers were not included. Thus, we regard it safe to assume that the most likely net causal effect here was *S*→*P.* It must be noted, however, that the set of criteria just described were only used for guiding the biological interpretation of results, but did not bias the derivation of such results in any way.

# References of supplementary text

1. R Development Core Team (2011) R: A Language and Environment for Statistical Computing Vienna, Austria: R Foundation for Statistical Computing. Available:http://www.R-project.org.

2. Benjamini Y, Hochberg Y (1995) Controlling the False Discovery Rate: A Practical and Powerful Approach to Multiple Testing. Journal of the Royal Statistical Society Series B (Methodological) 57: 289–300.

3. Rotival M, Zeller T, Wild PS, Maouche S, Szymczak S, et al. (2011) Integrating genome-wide genetic variations and monocyte expression data reveals trans-regulated gene modules in humans. PLoS Genet 7: e1002367. doi:10.1371/journal.pgen.1002367.

4. Watkins NA, Gusnanto A, de Bono B, De S, Miranda-Saavedra D, et al. (2009) A HaemAtlas: characterizing gene expression in differentiated human blood cells. Blood 113: e1 -e9. doi:10.1182/blood-2008-06-162958.

5. Spirtes P (2010) Introduction to Causal Inference. J Mach Learn Res 99: 1643–1662.

6. Chickering DM (2002) Learning equivalence classes of Bayesian-network structures. The Journal of Machine Learning Research 2: 445–498.

7. Schadt EE, Lamb J, Yang X, Zhu J, Edwards S, et al. (2005) An integrative genomics approach to infer causal associations between gene expression and disease. Nat Genet 37: 710–717.

8. Uhl GR, Drgon T, Li C-Y, Johnson C, Liu Q-R (2009) Smoking and smoking cessation in disadvantaged women: assessing genetic contributions. Drug Alcohol Depend 104 Suppl 1: S58–63. doi:10.1016/j.drugalcdep.2009.03.012.

9. Uhl GR, Drgon T, Johnson C, Ramoni MF, Behm FM, et al. (2010) Genome-wide association for smoking cessation success in a trial of precessation nicotine replacement. Mol Med 16: 513–526. doi:10.2119/molmed.2010.00052.
