# Supplementary material for: Graphical Modeling of Gene Expression in Monocytes Suggests Molecular Mechanisms Explaining Increased Atherosclerosis in Smokers
Source: PLoS One. 2013 Jan 23;8(1):e50888. doi: 10.1371/journal.pone.0050888 (PMC3553098; doi:10.1371/journal.pone.0050888)

Supplementary Text S2

Network plots for all ICA modules.

Verdugo *et al.*

The following graphs represent network skeletons for each ICA module estimated by the PC-algorithm applied to 1000 data bootstraps (see Methods in main text). Edges among variables are drawn if detected in at least 60% of data resamples. The recovery percentages are indicated to the right of the medial section of each edge. Line thickness is proportional to the edge's partial correlation. Black edges denote positive and pink edges negative partial correlations. Plaques and risk factors are in blue. Genes directly connected to smoking are in green and those directly connected to plaques are in orange. Other genes are in gray. Only genes that are involved in the shortest paths connecting smoking to plaques are shown. Genes connected to both smoking and plaques are in red.

Network Skeleton for Pattern 4

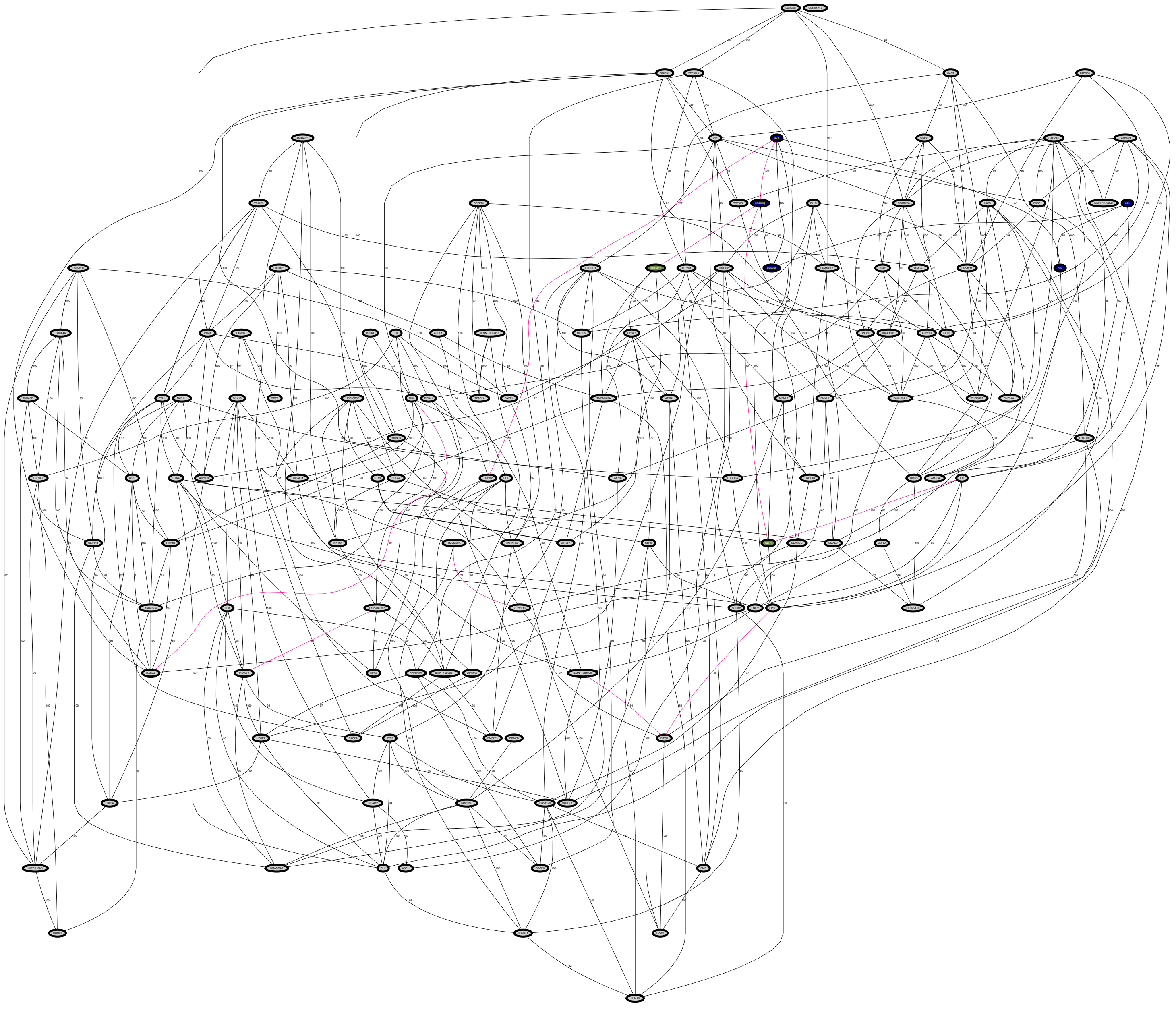

Network Skeleton for Pattern 11

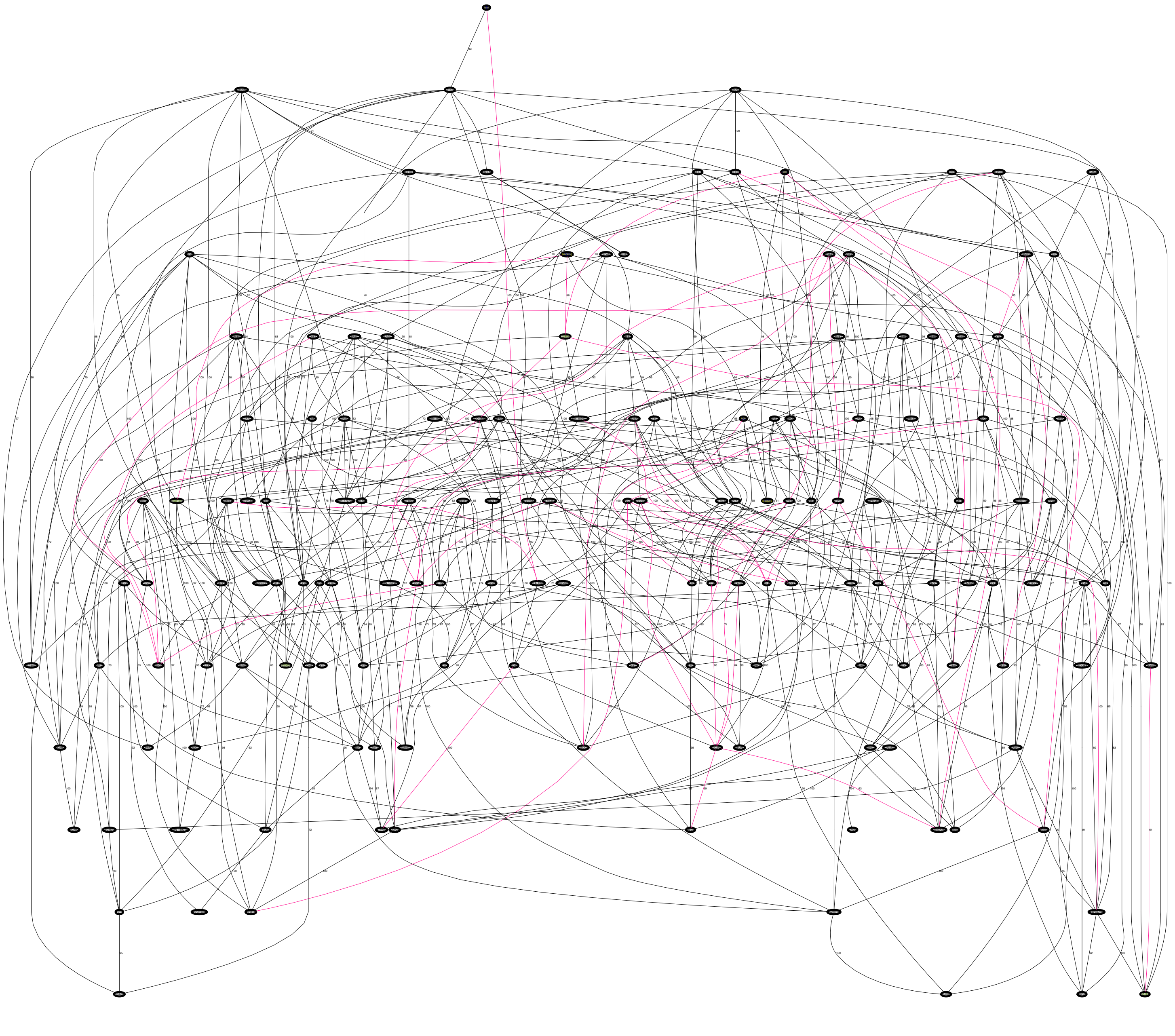

Network Skeleton for Pattern 12

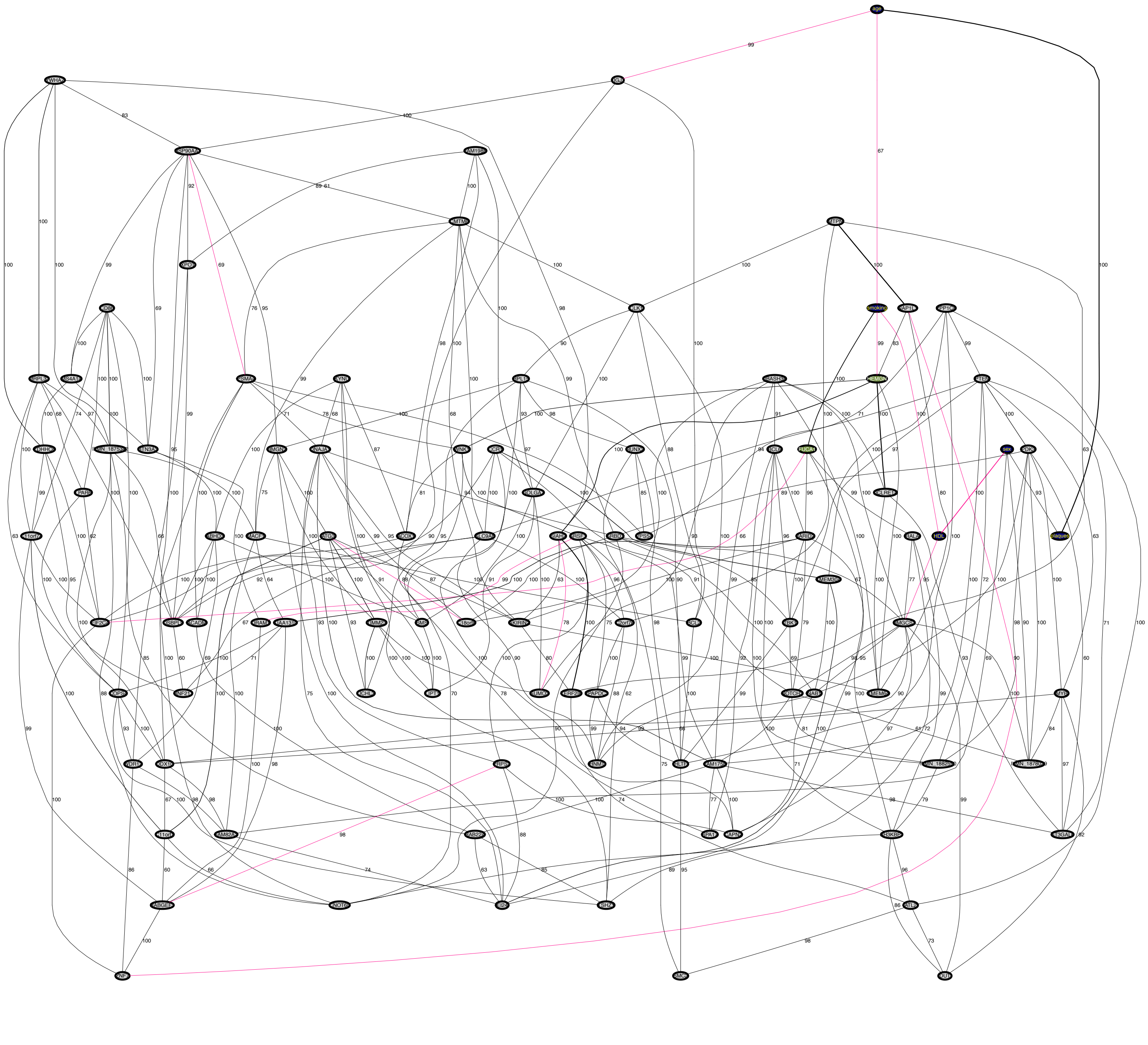

Network Skeleton for Pattern 14

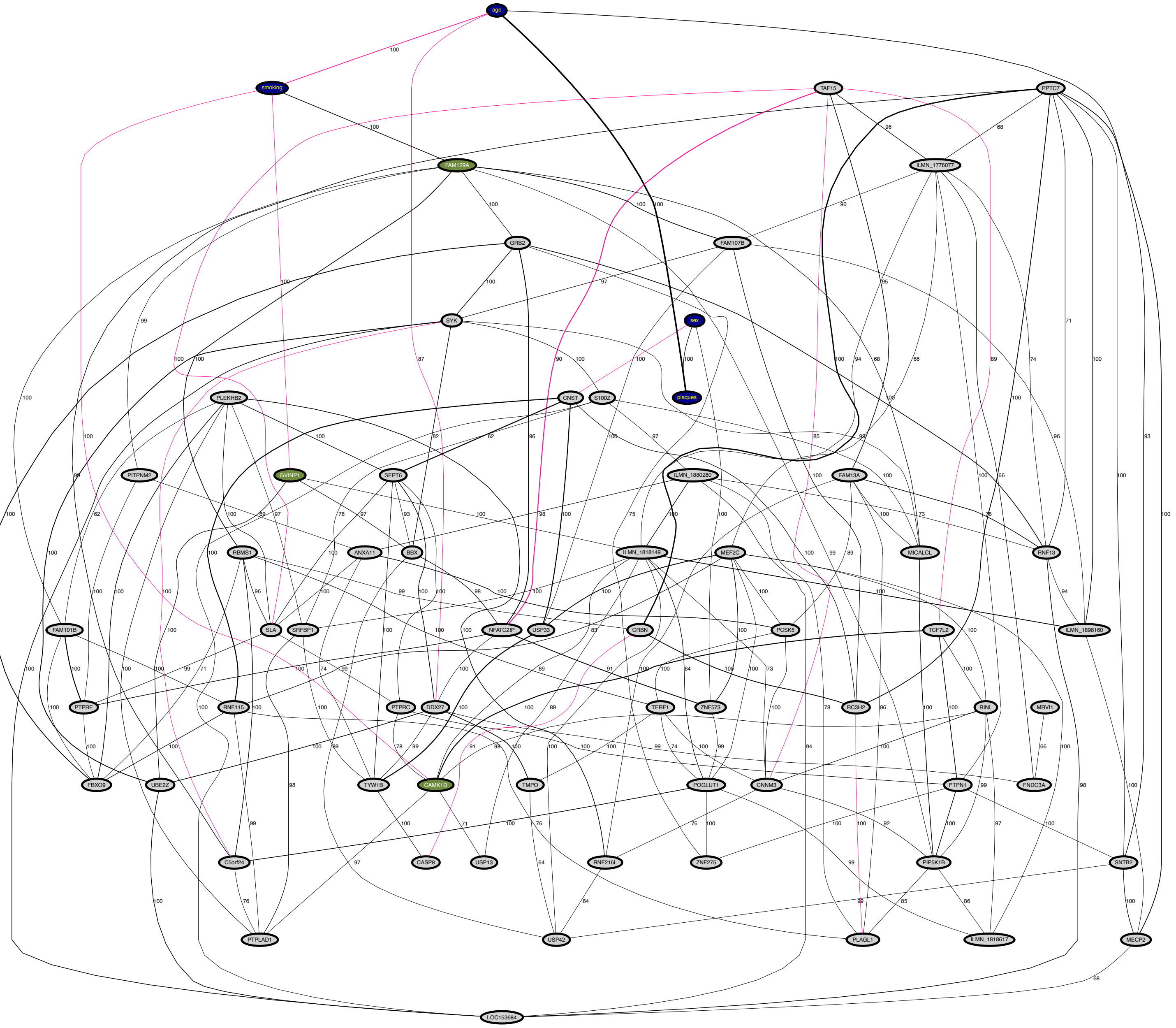

Network Skeleton for Pattern 15

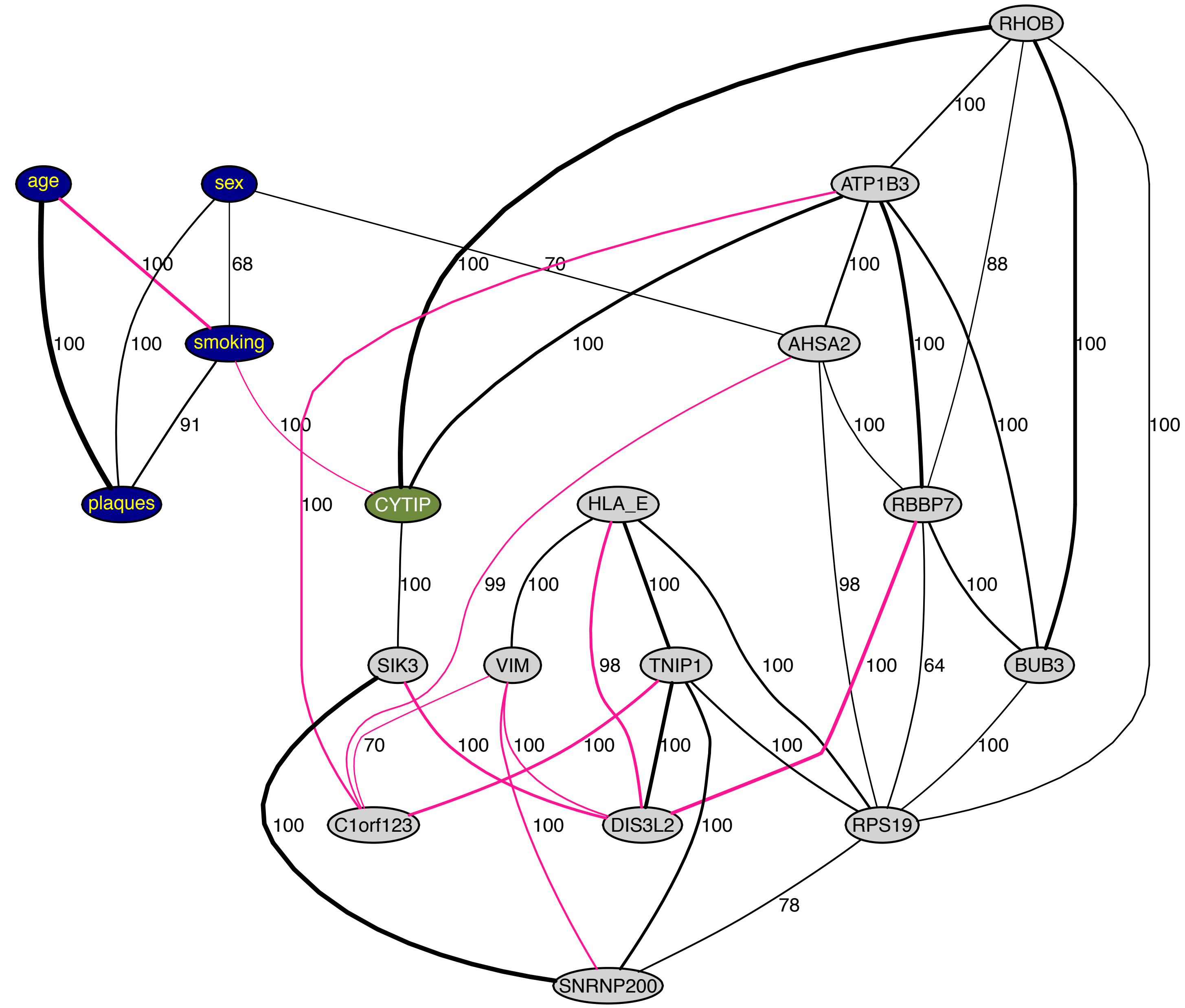

Network Skeleton for Pattern 17

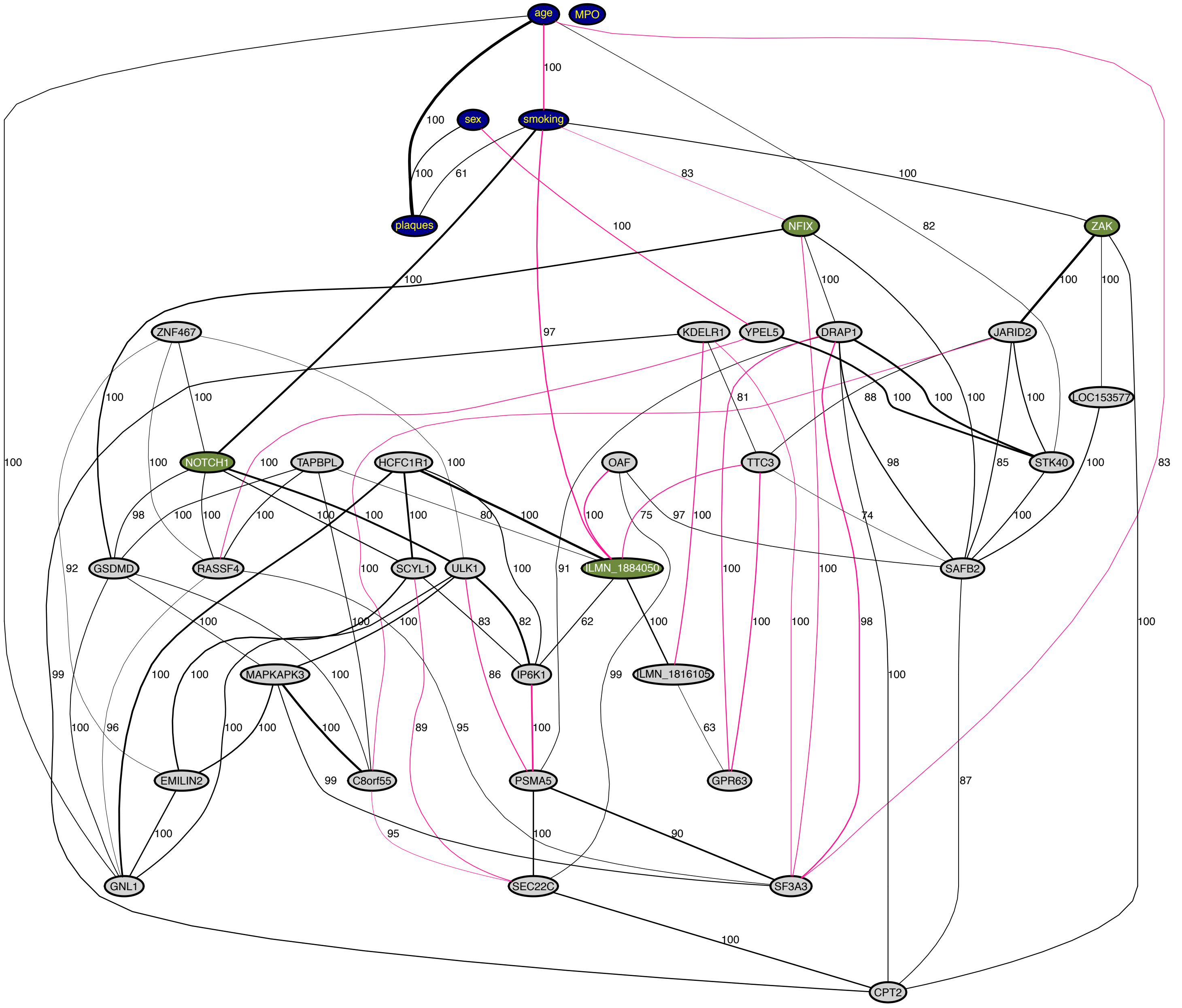

Network Skeleton for Pattern 18

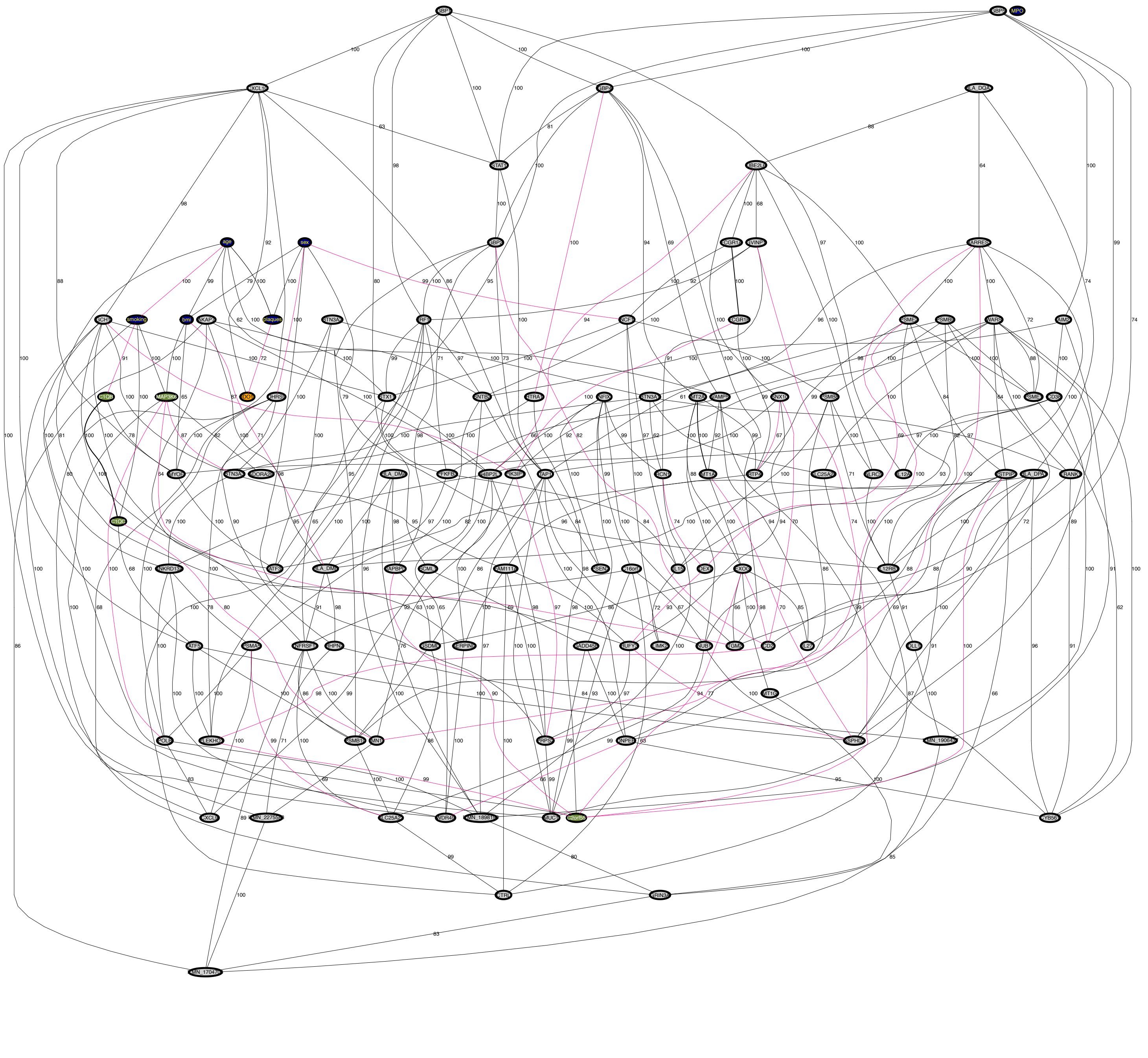

Network Skeleton for Pattern 19

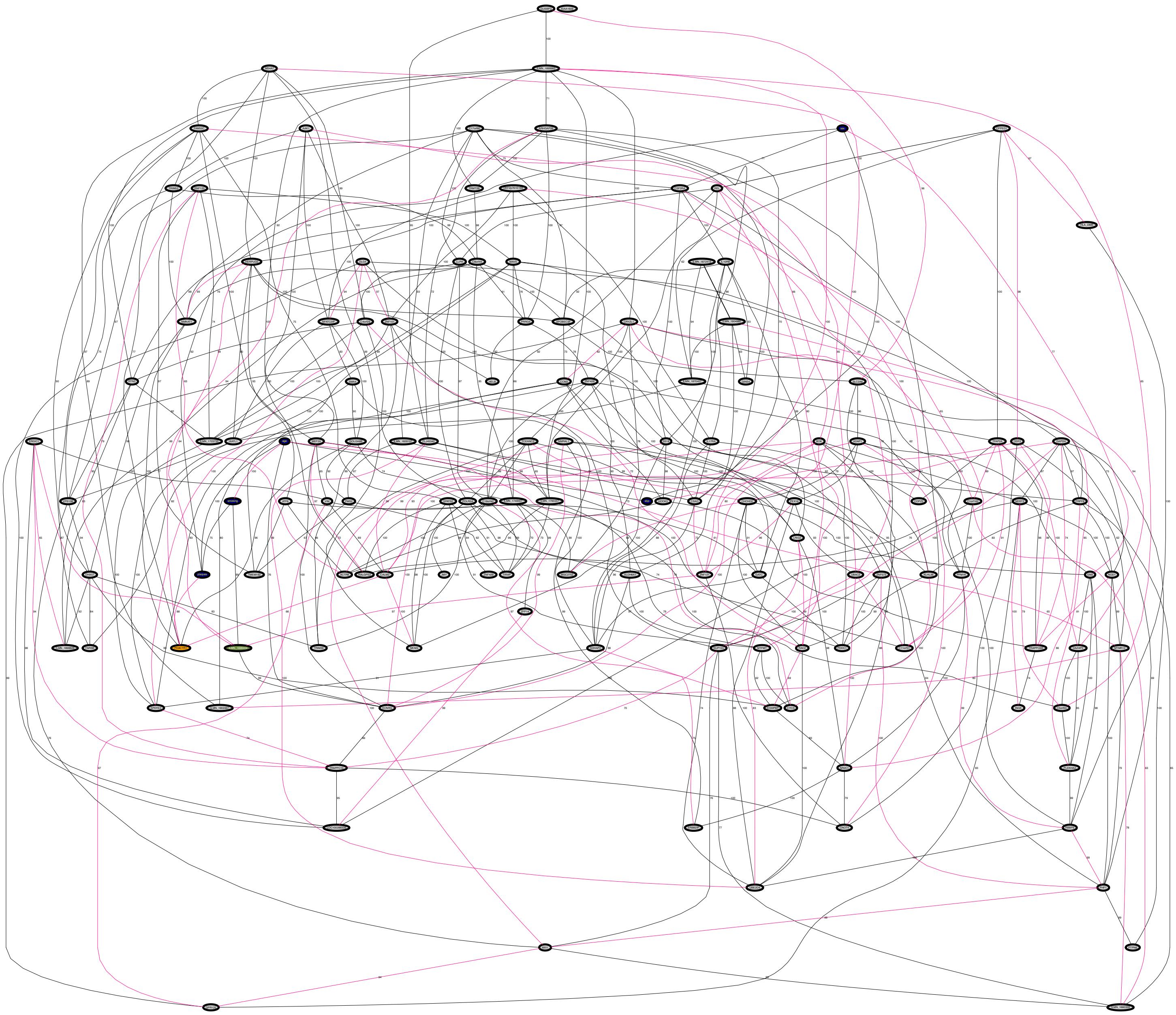

Network Skeleton for Pattern 21

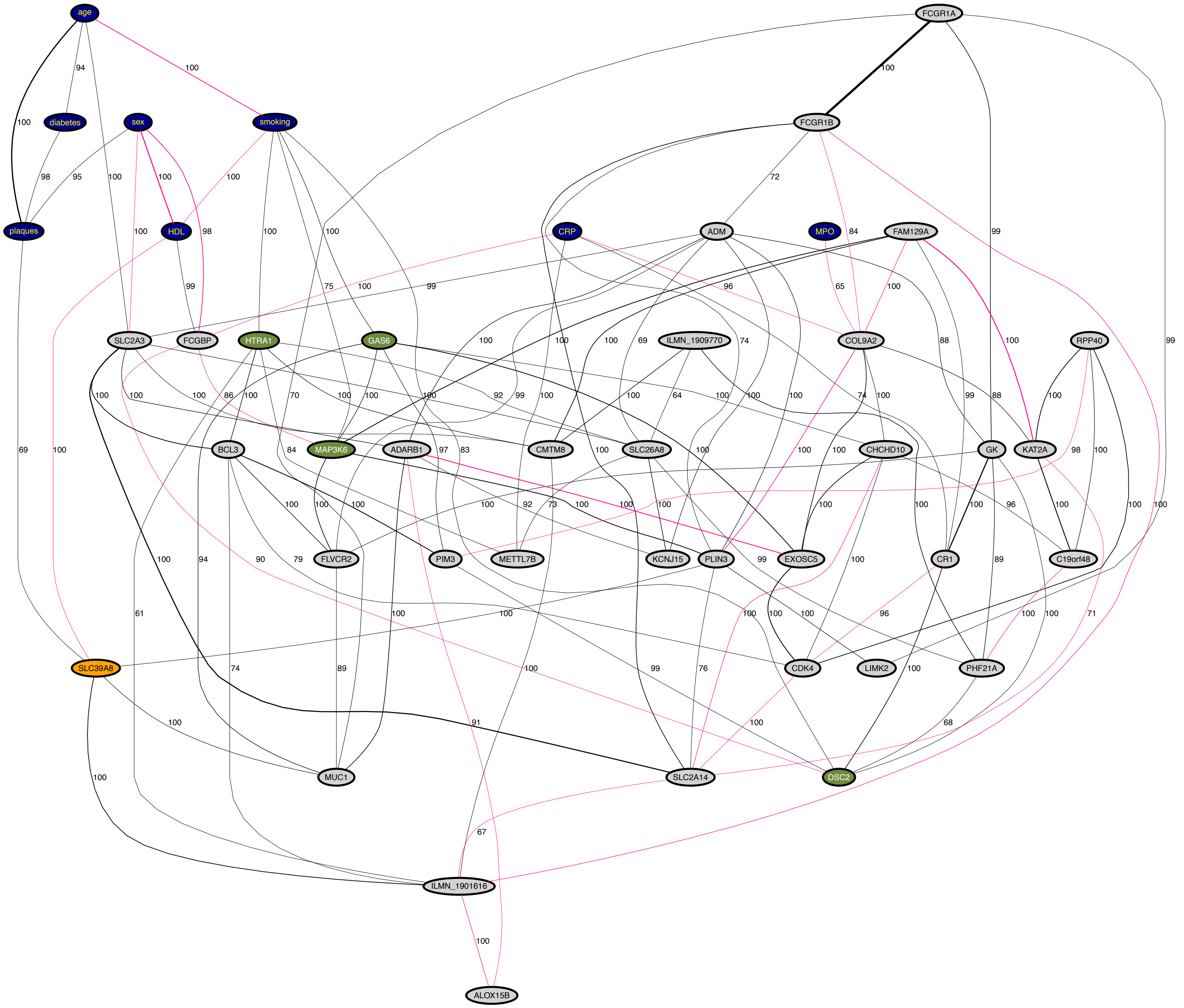

Network Skeleton for Pattern 23

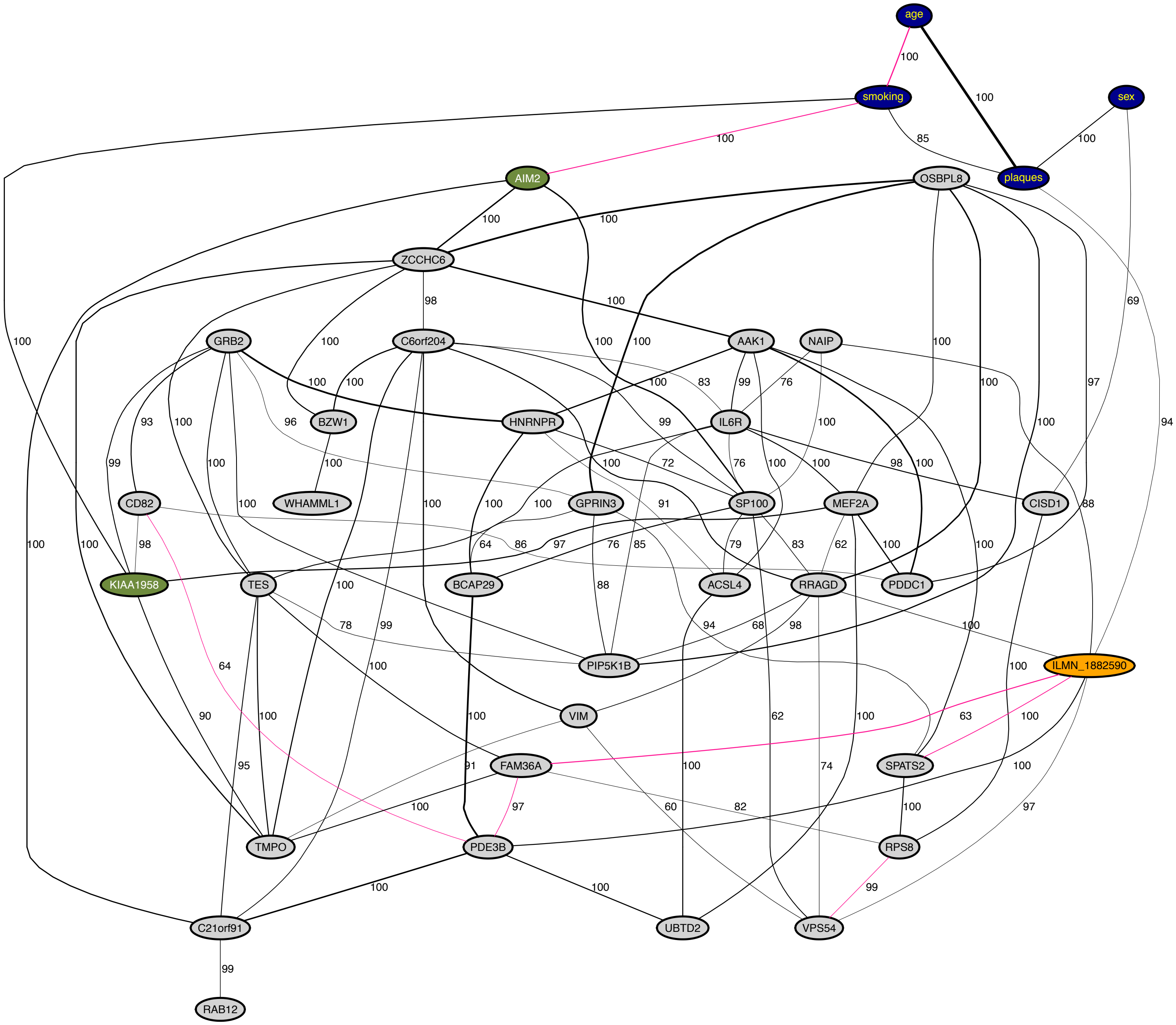

Network Skeleton for Pattern 27

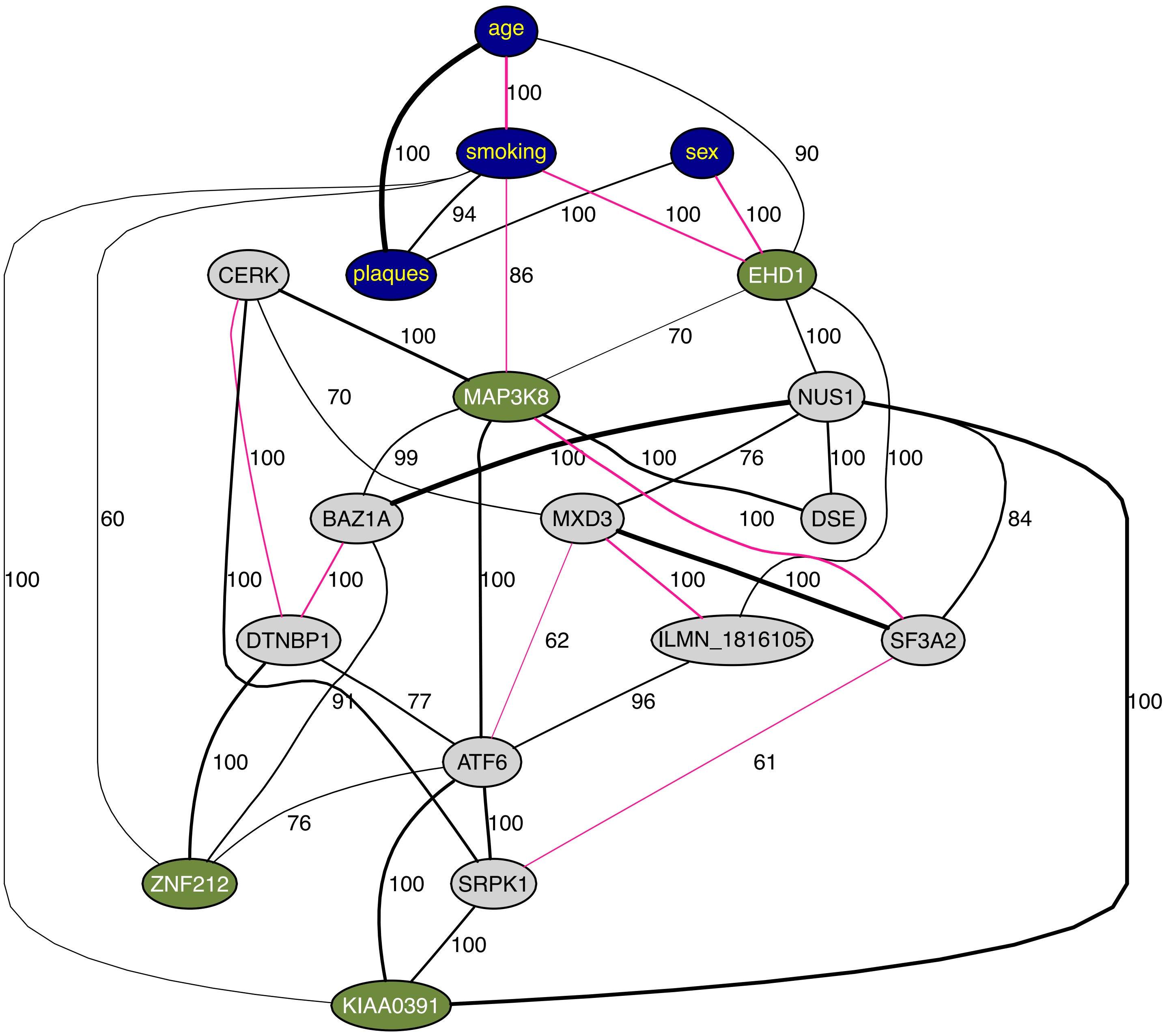

Network Skeleton for Pattern 28

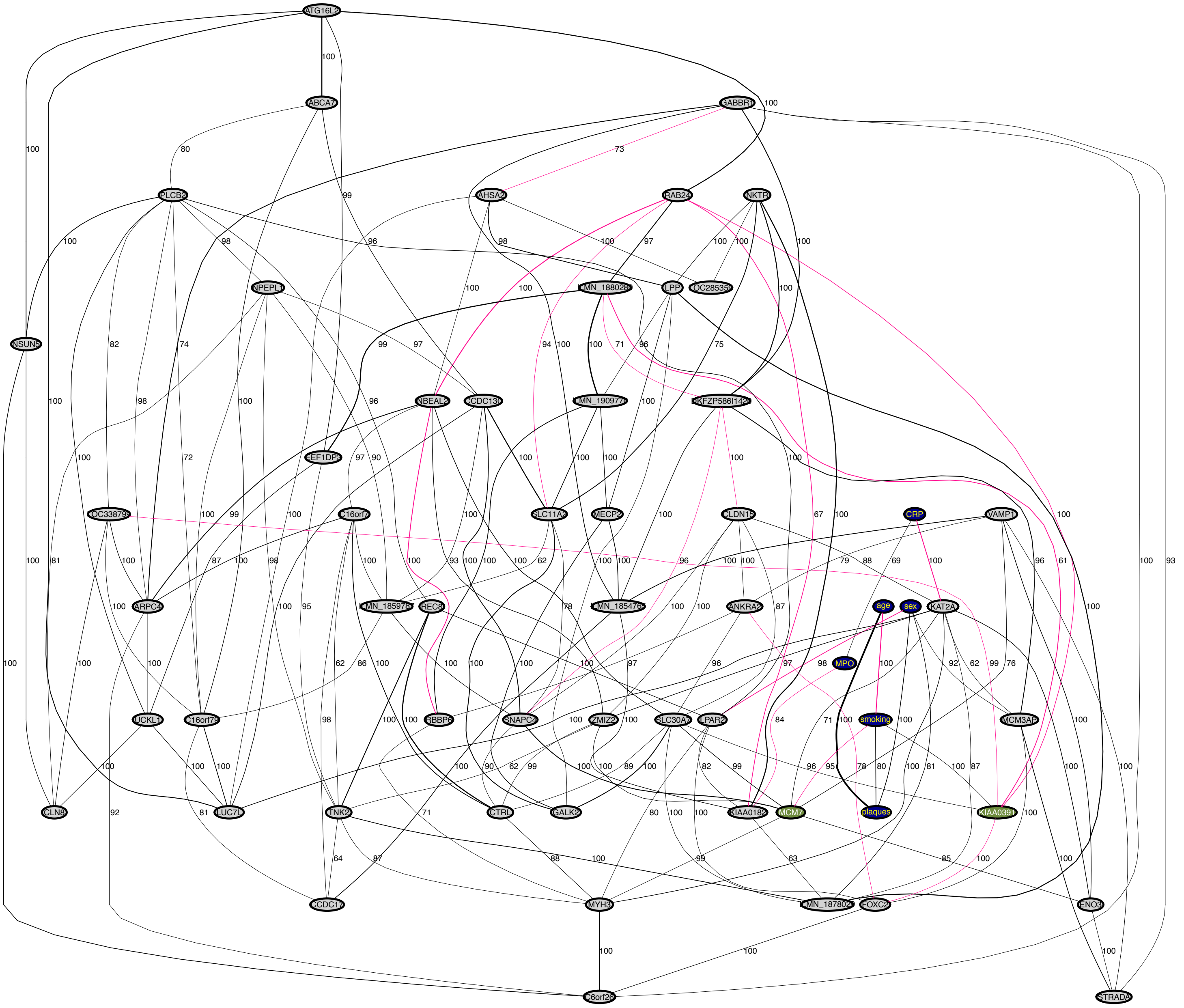

Network Skeleton for Pattern 29

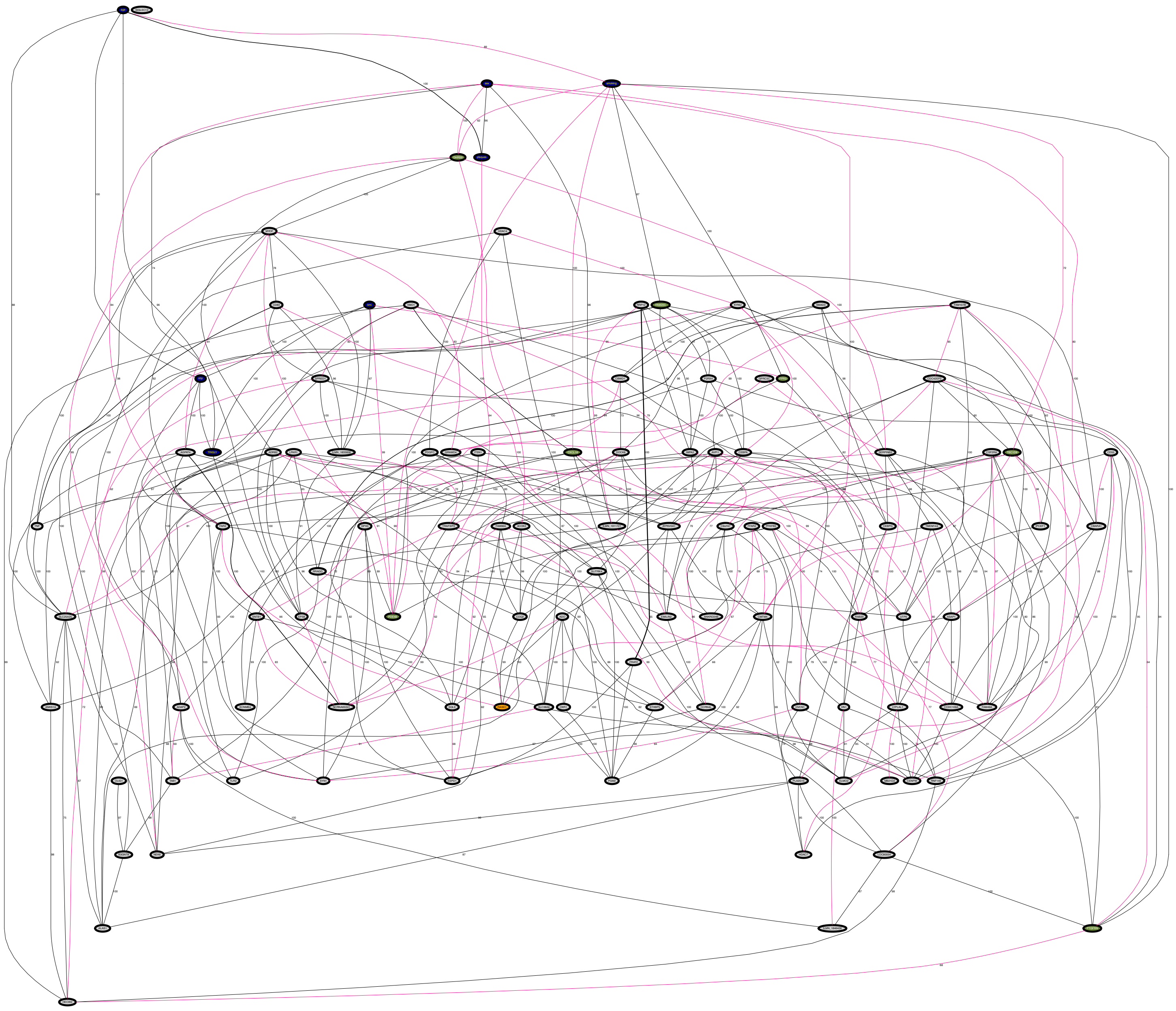

Network Skeleton for Pattern 30

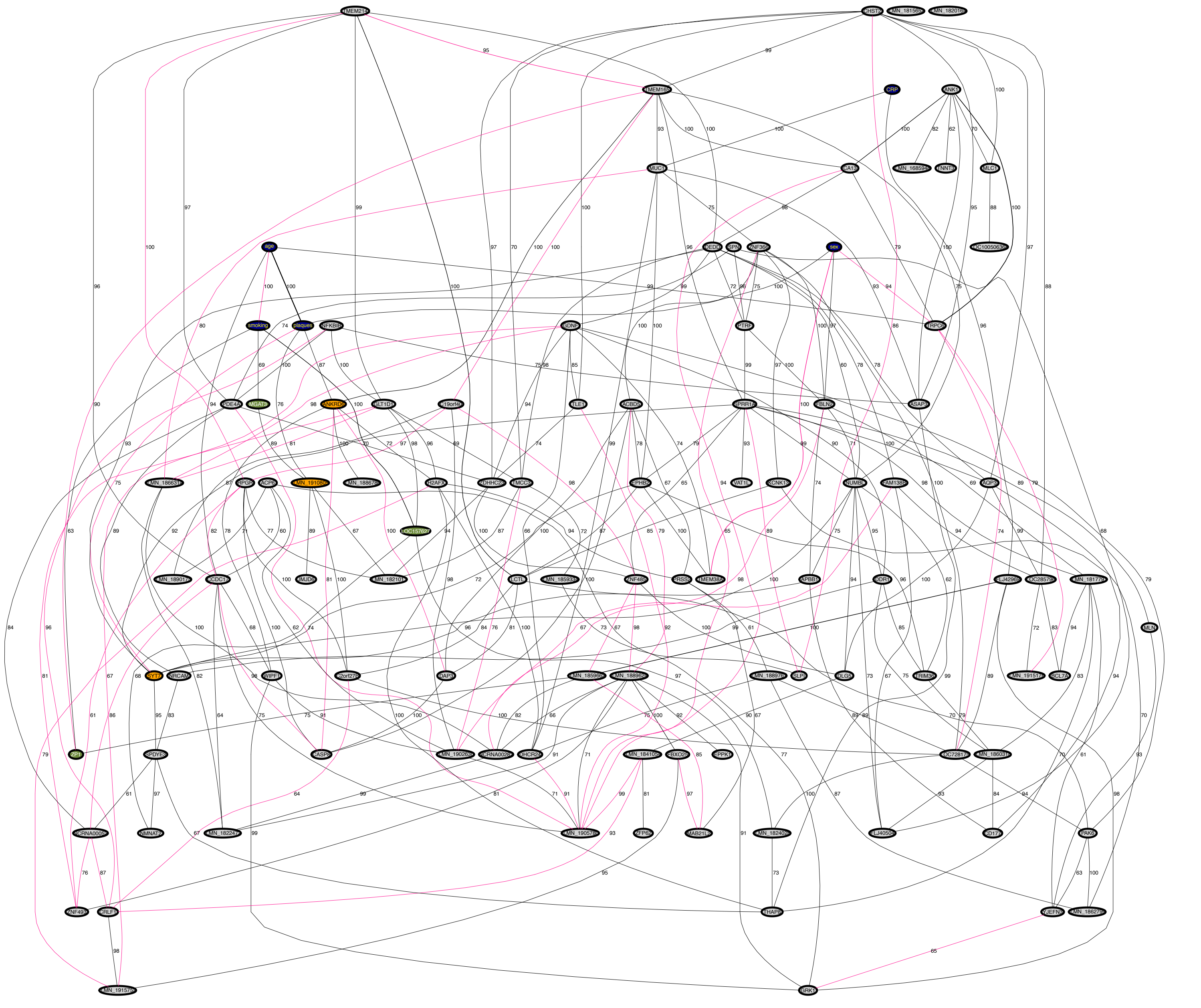

Network Skeleton for Pattern 31

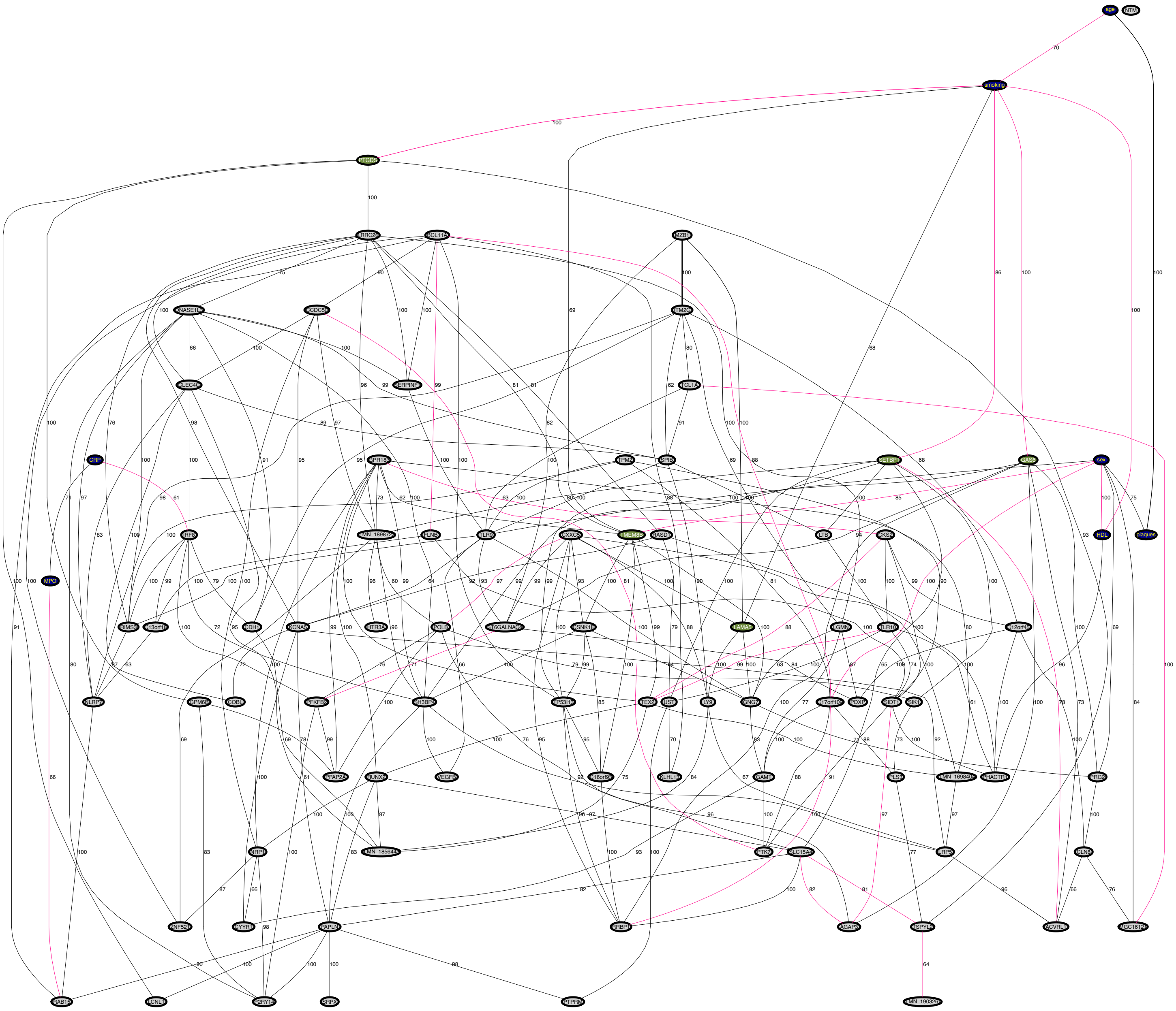

Network Skeleton for Pattern 33

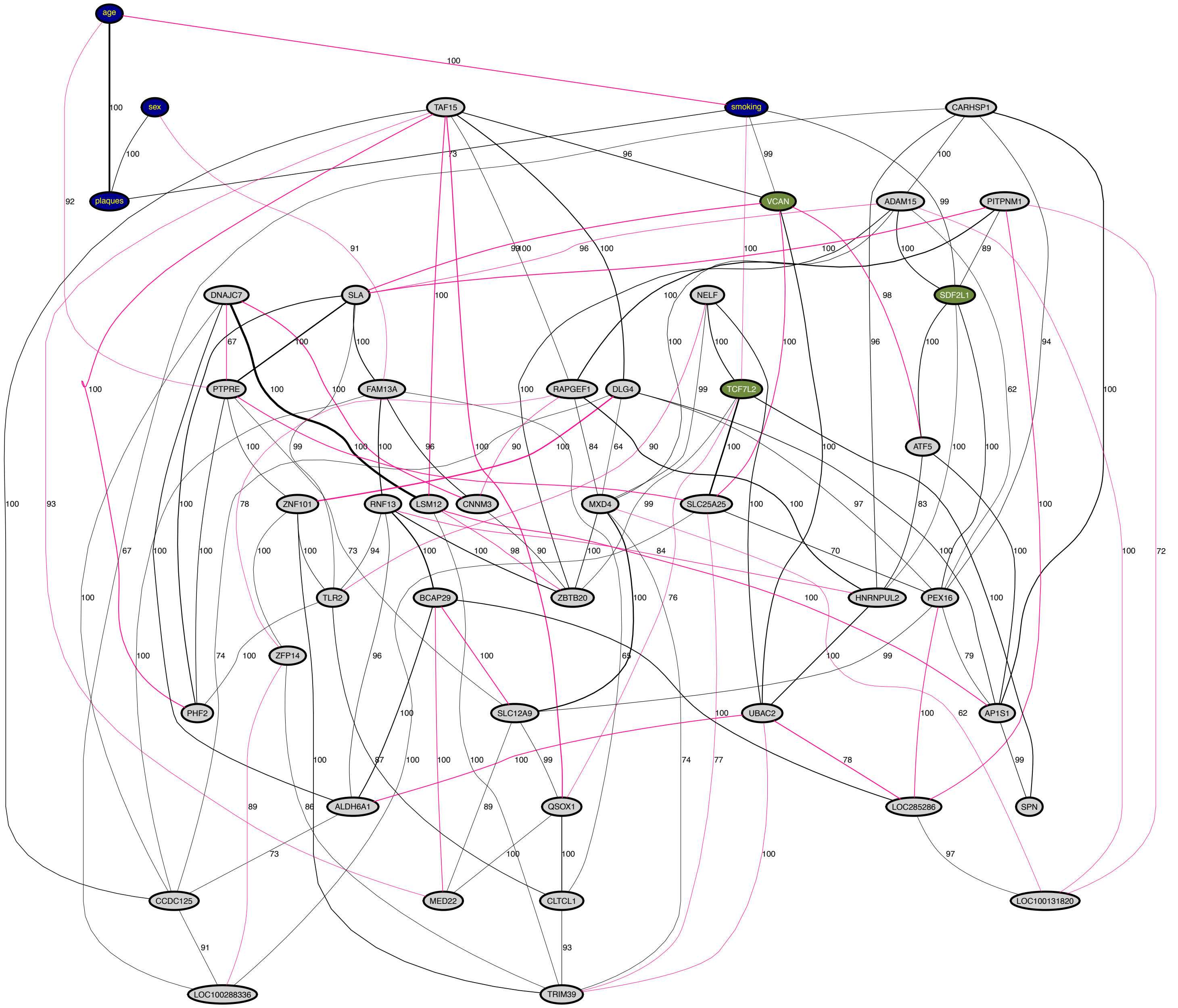

Network Skeleton for Pattern 34

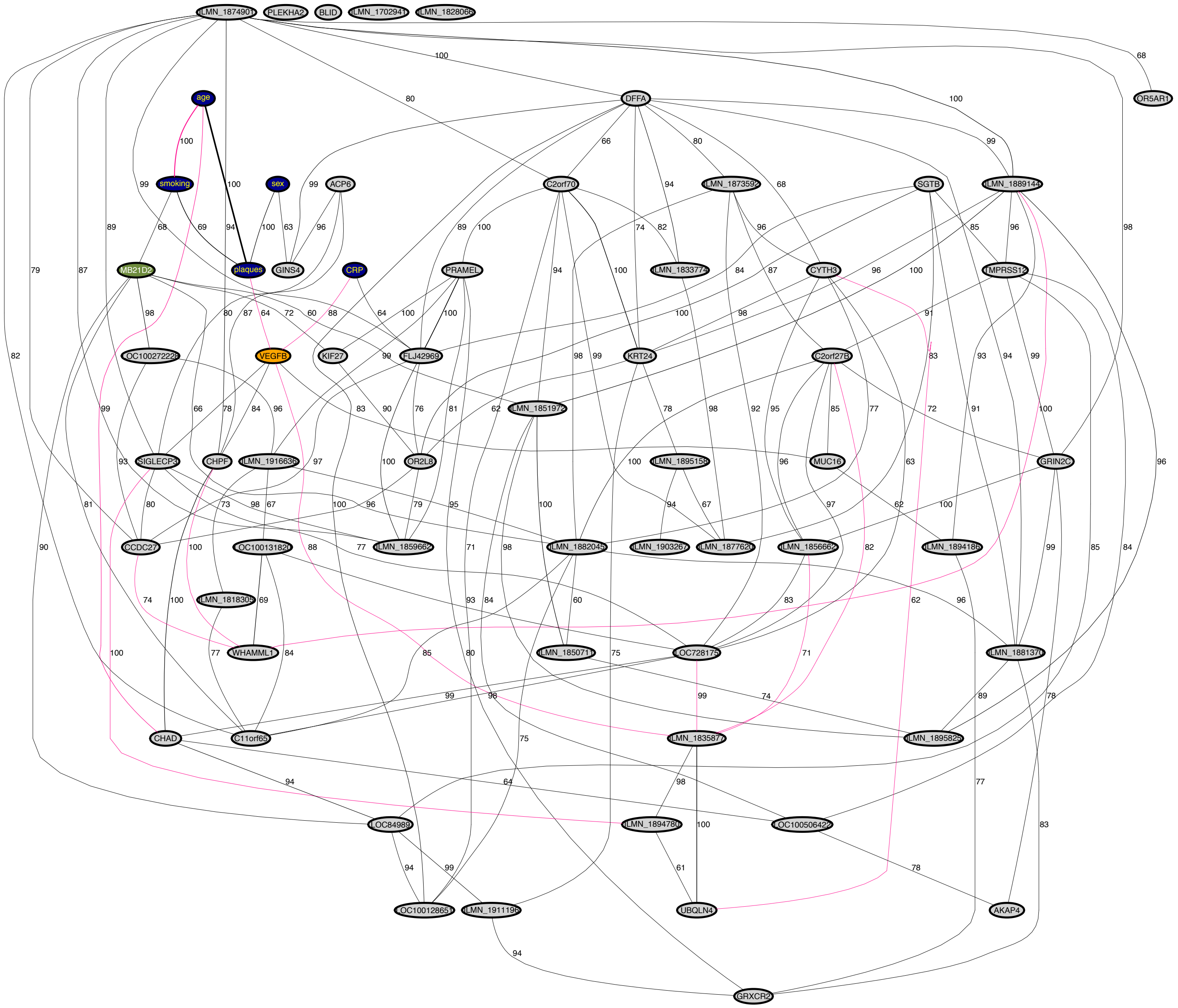

Network Skeleton for Pattern 36

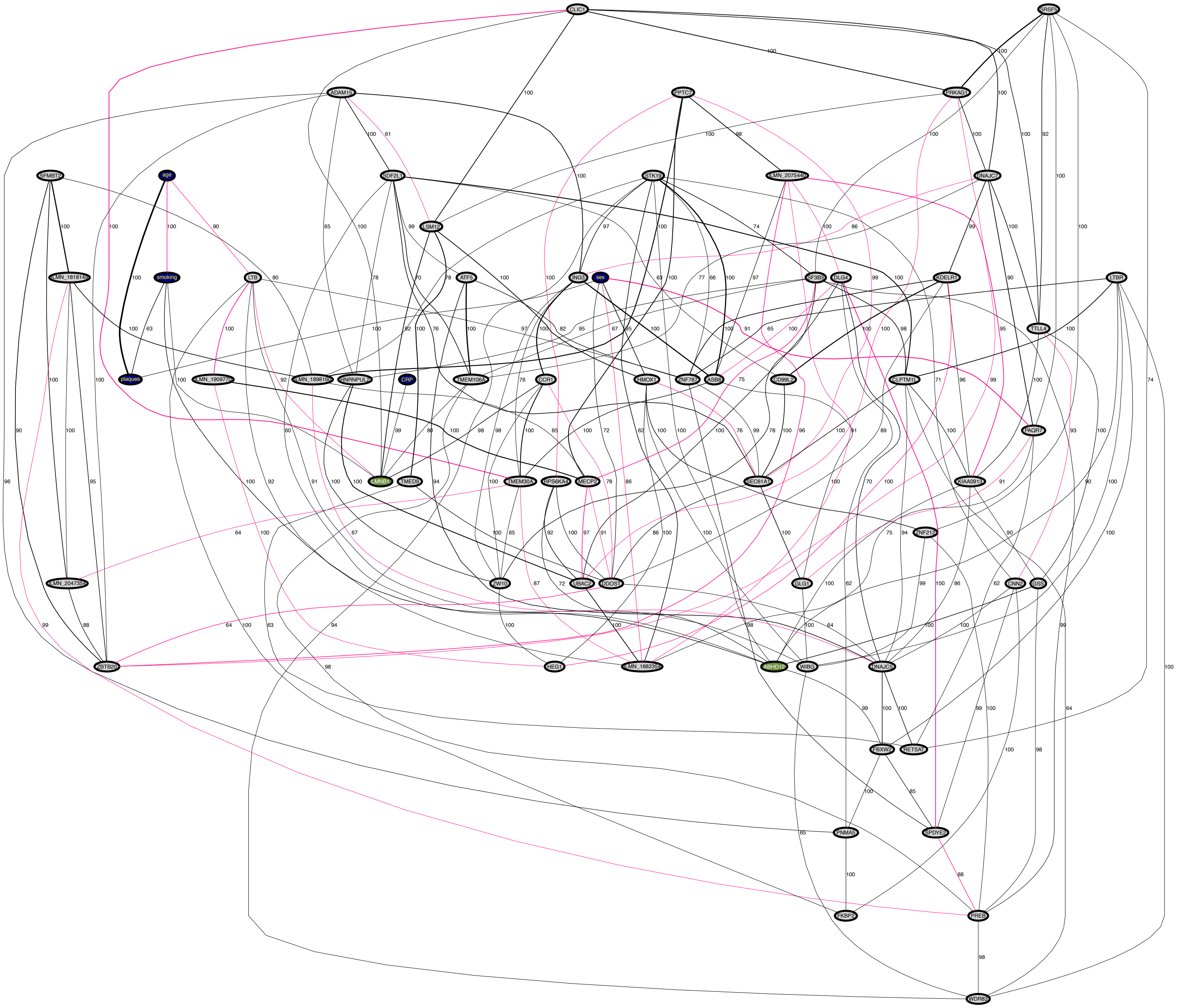

Network Skeleton for Pattern 39

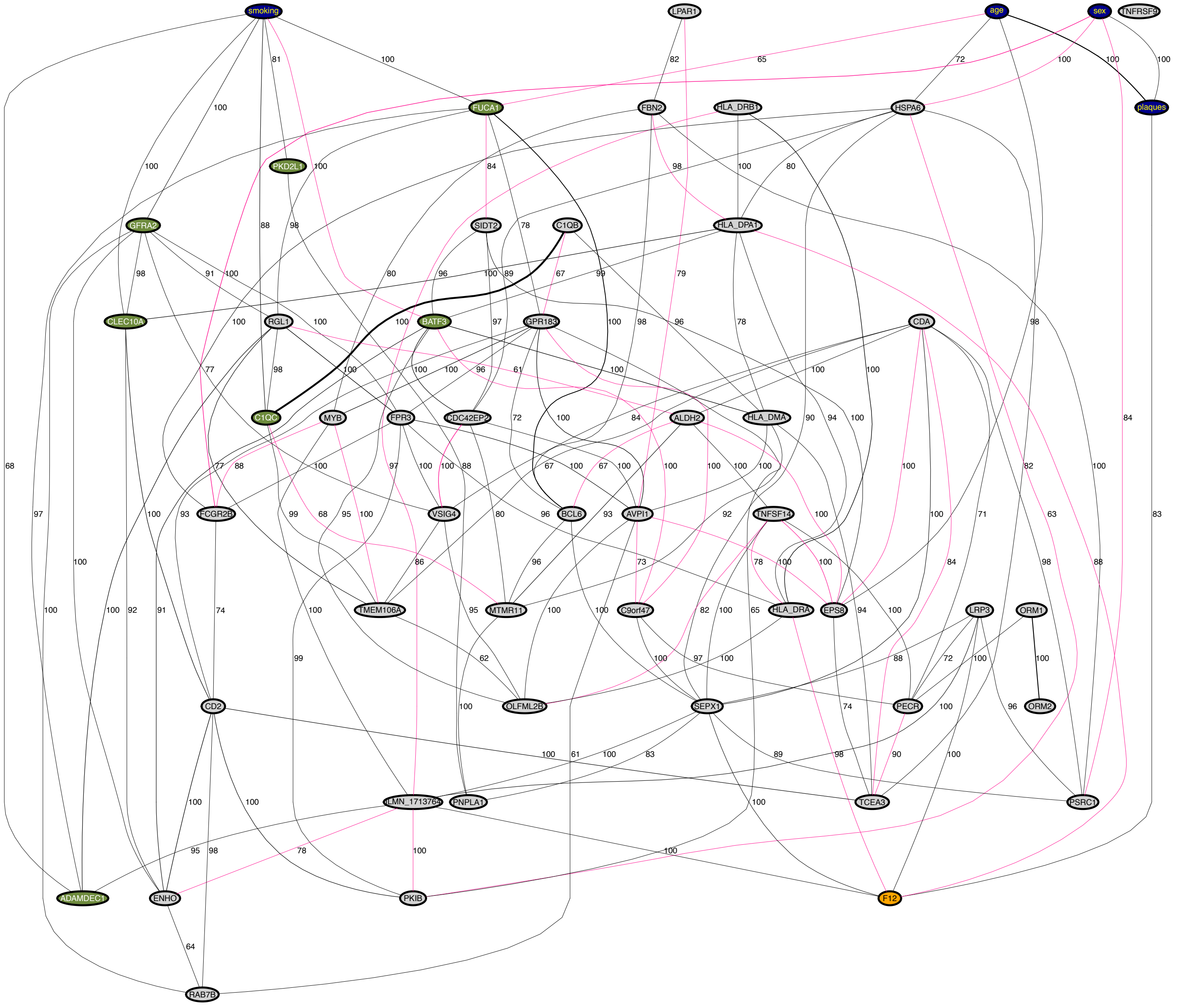

Network Skeleton for Pattern 41

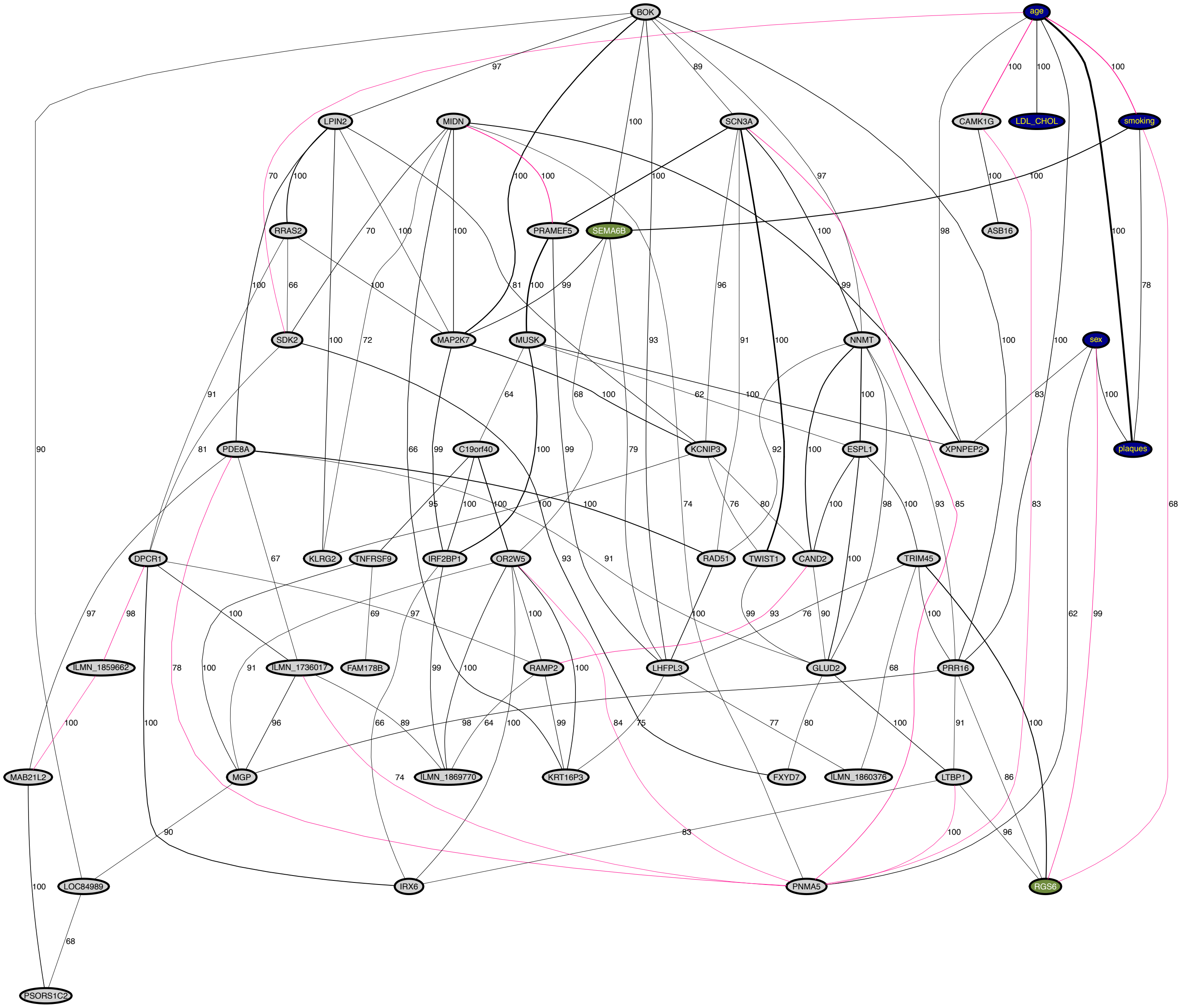

Network Skeleton for Pattern 42

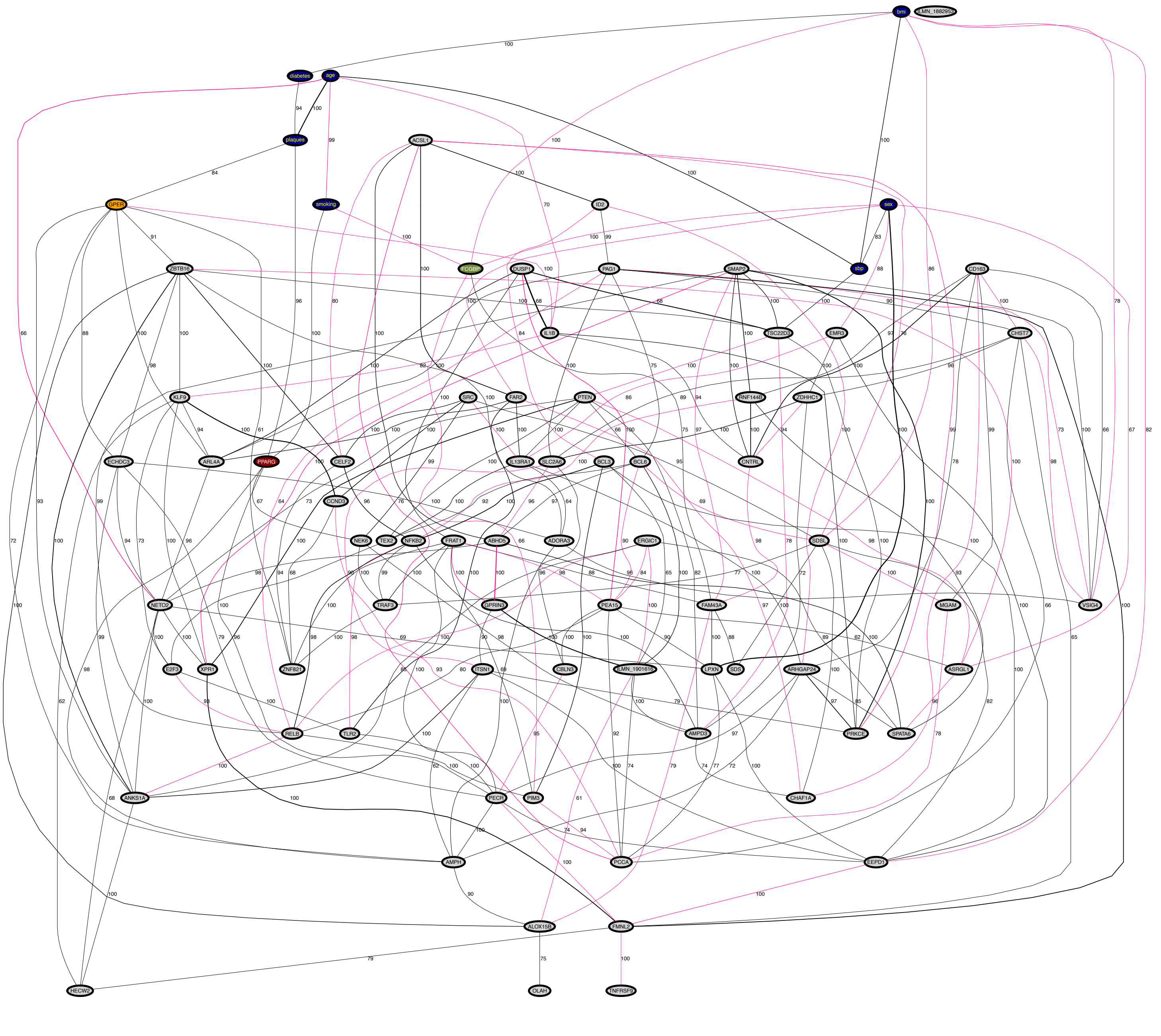

Network Skeleton for Pattern 43

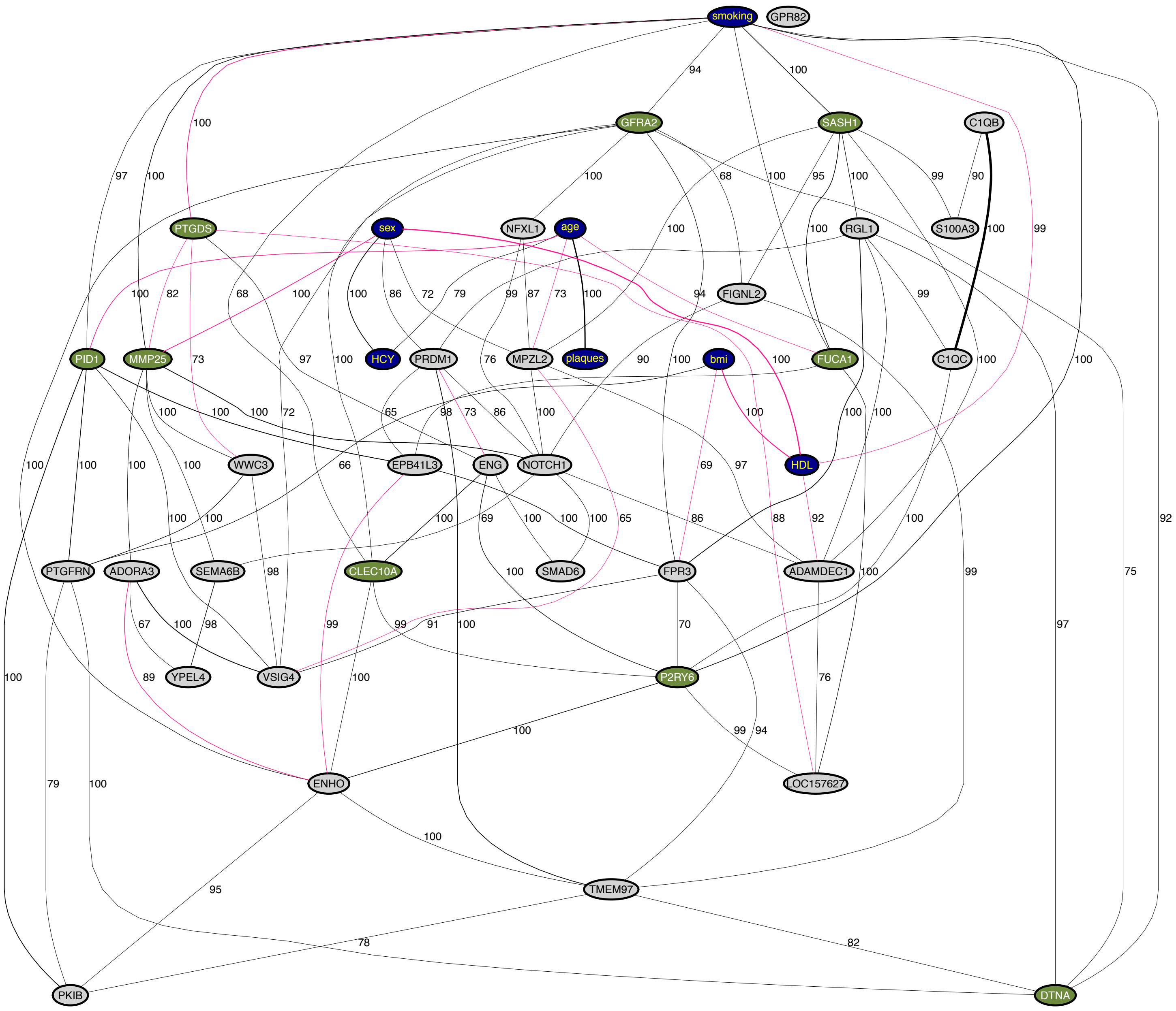

Network Skeleton for Pattern 45

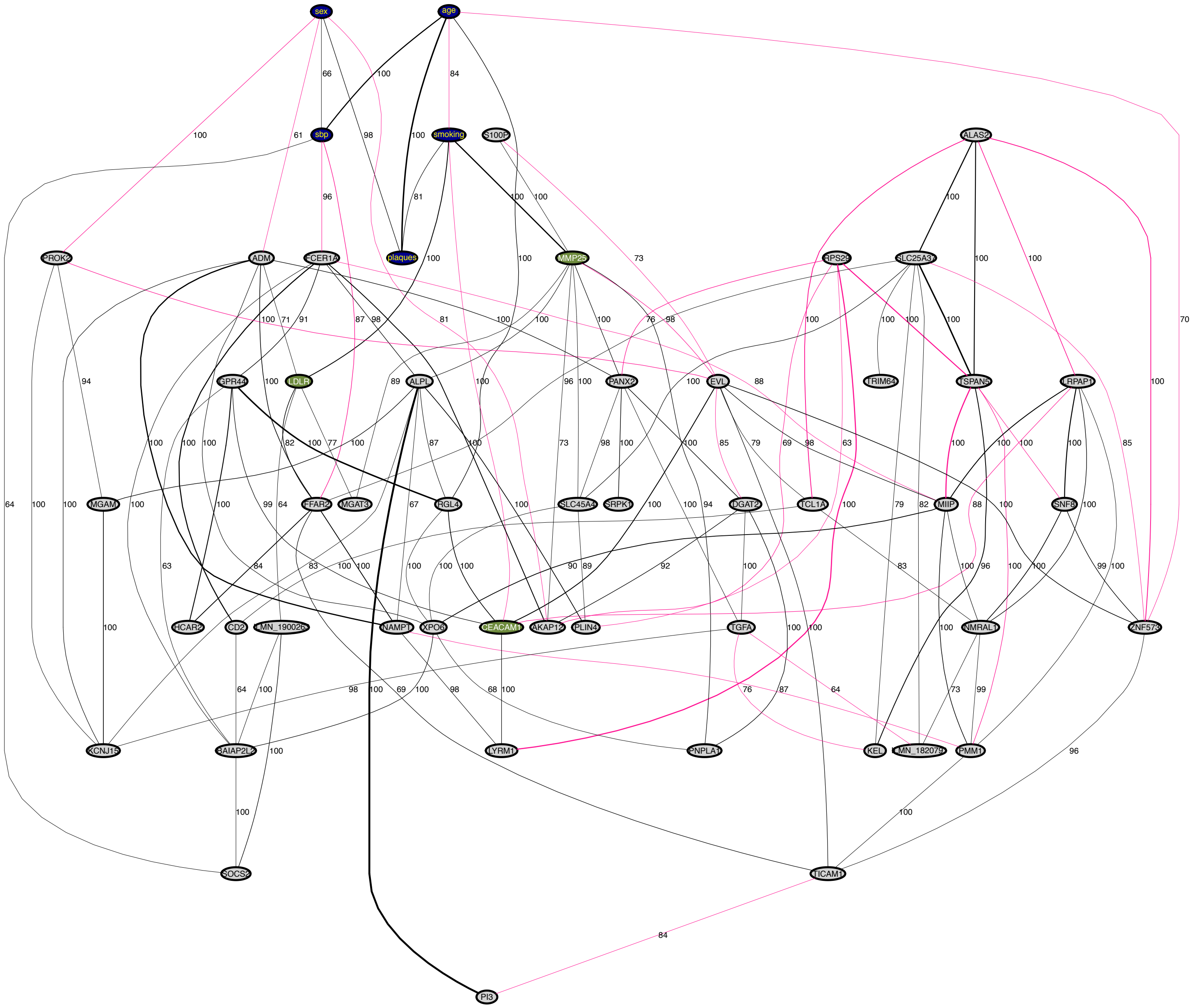

Network Skeleton for Pattern 48

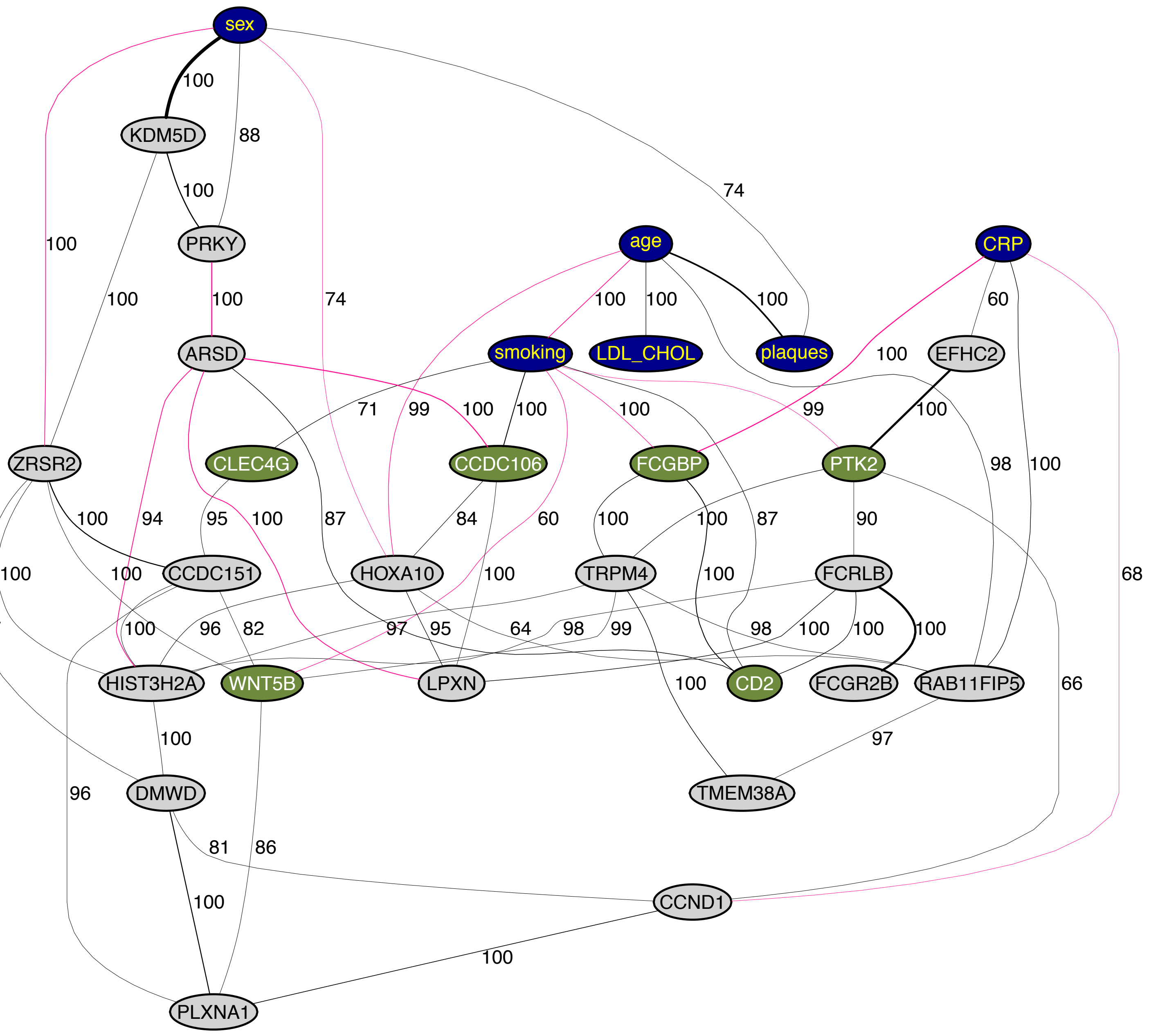

Network Skeleton for Pattern 49

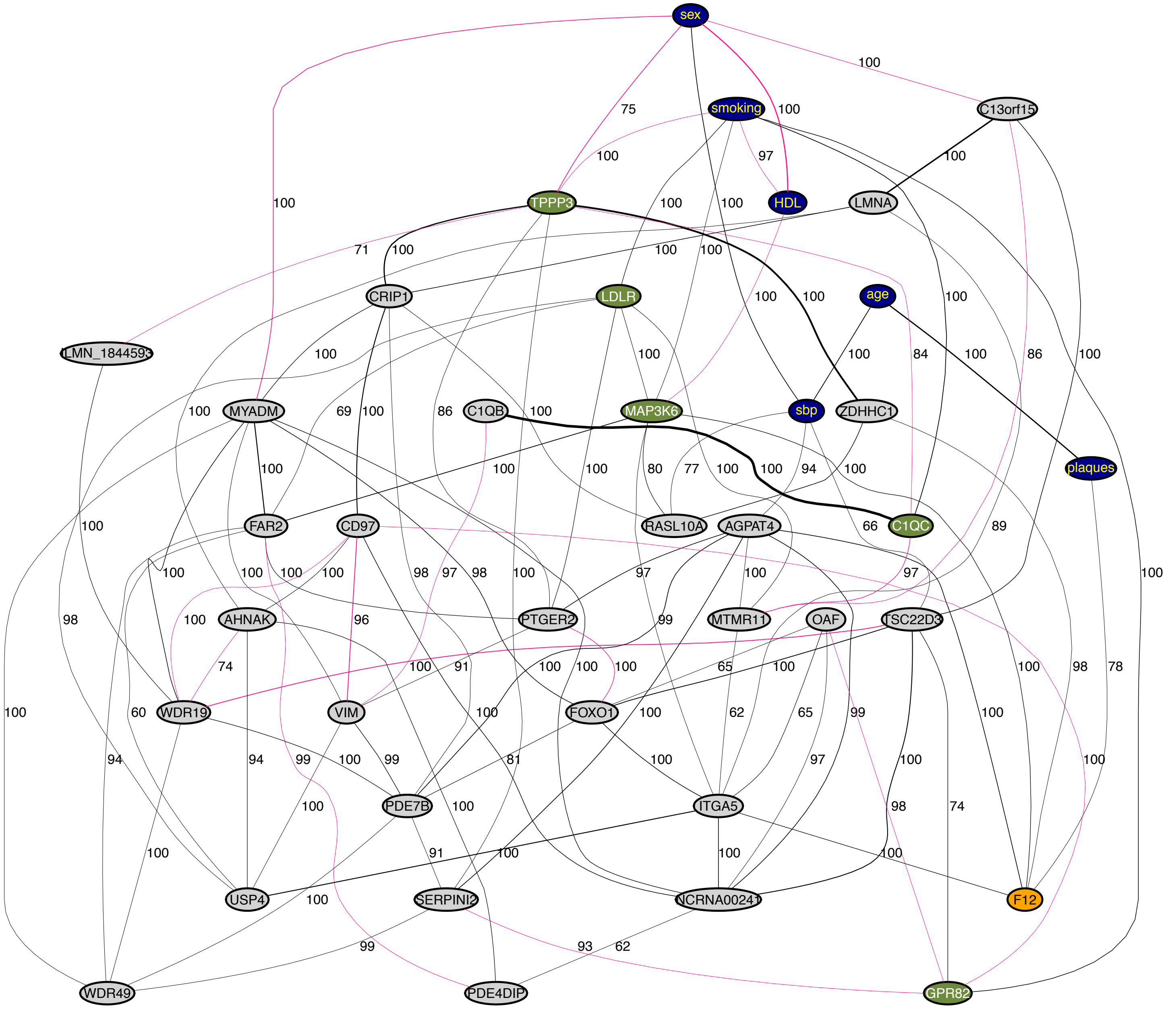

Network Skeleton for Pattern 51

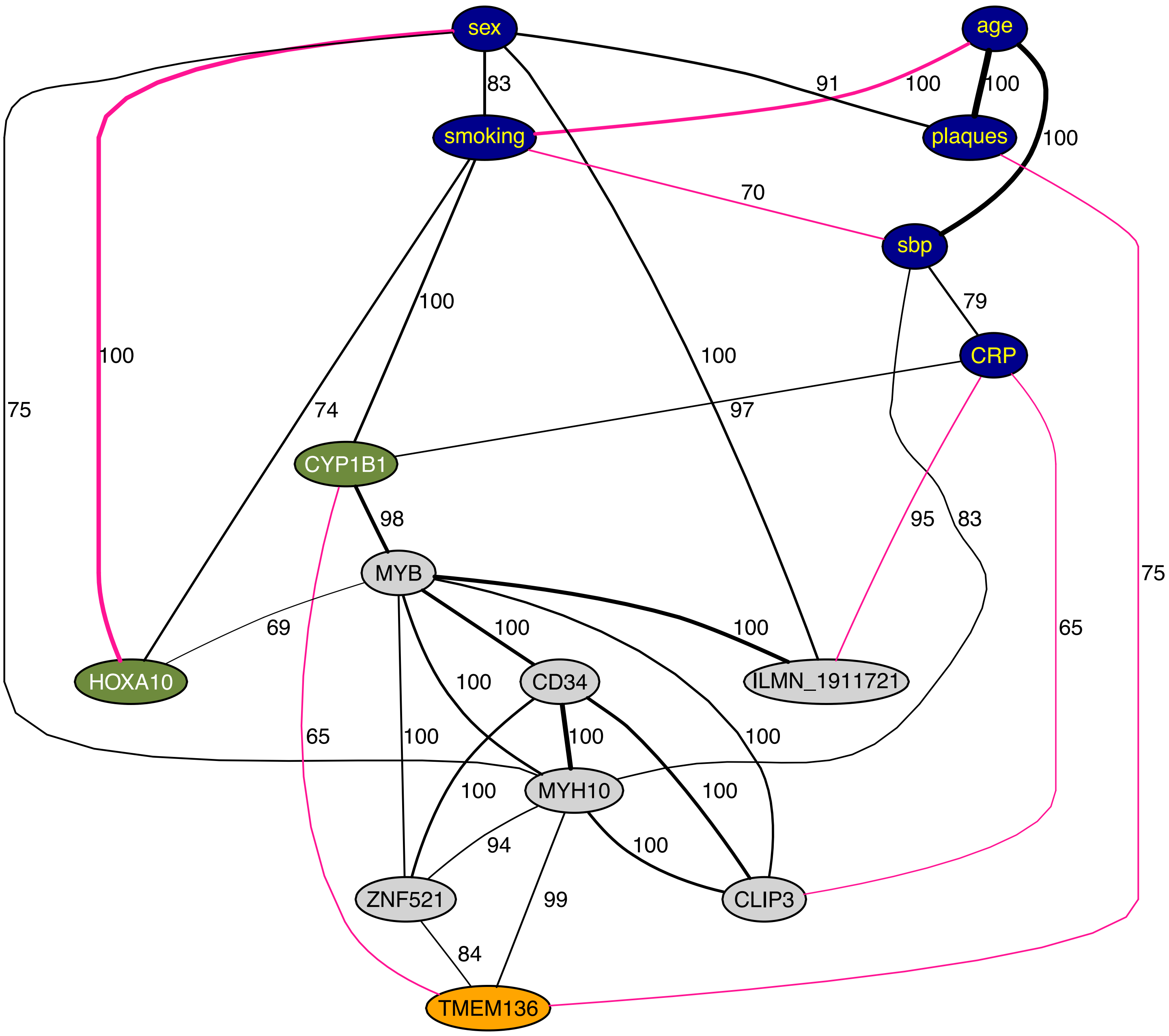

Network Skeleton for Pattern 52

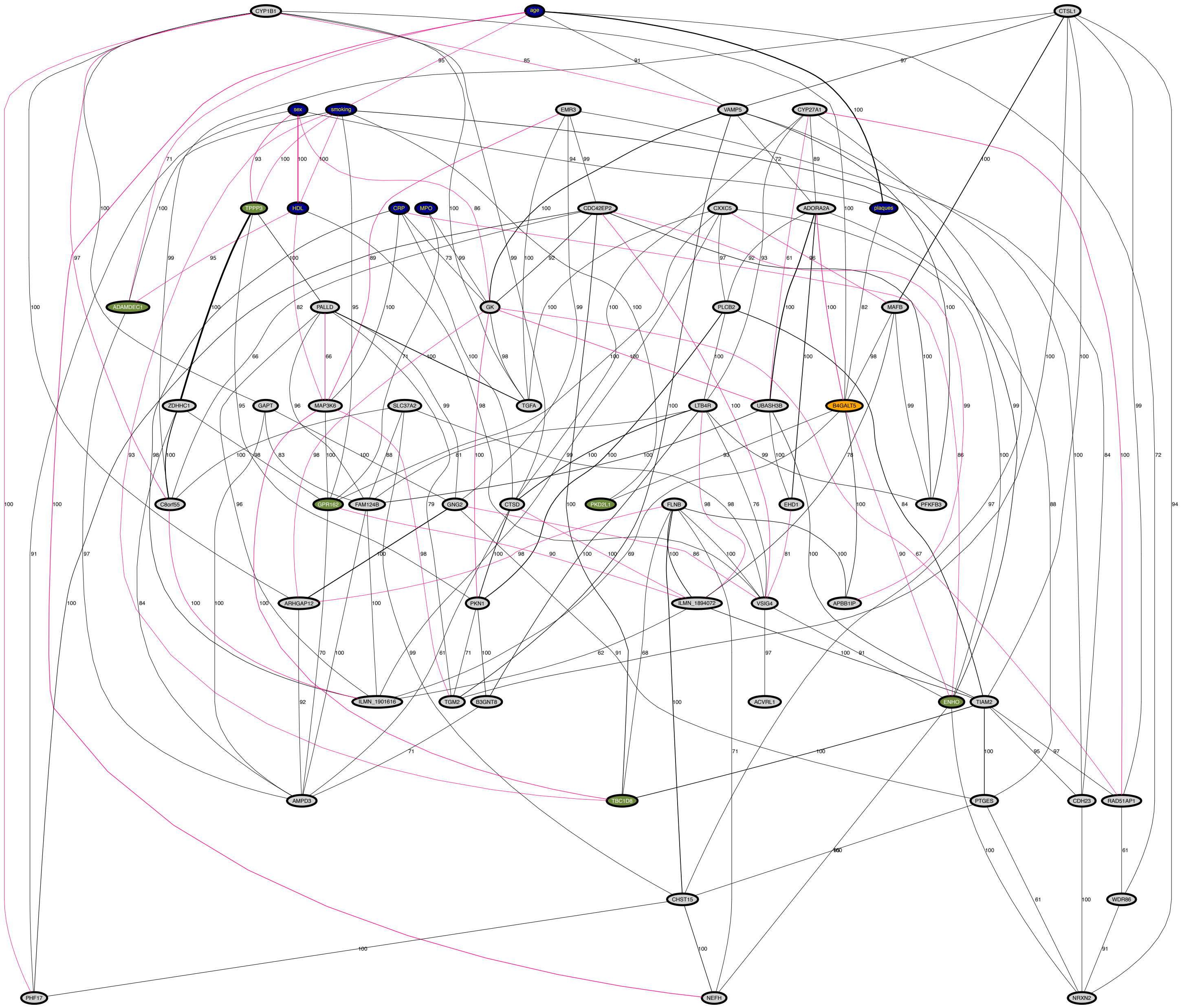

Network Skeleton for Pattern 54

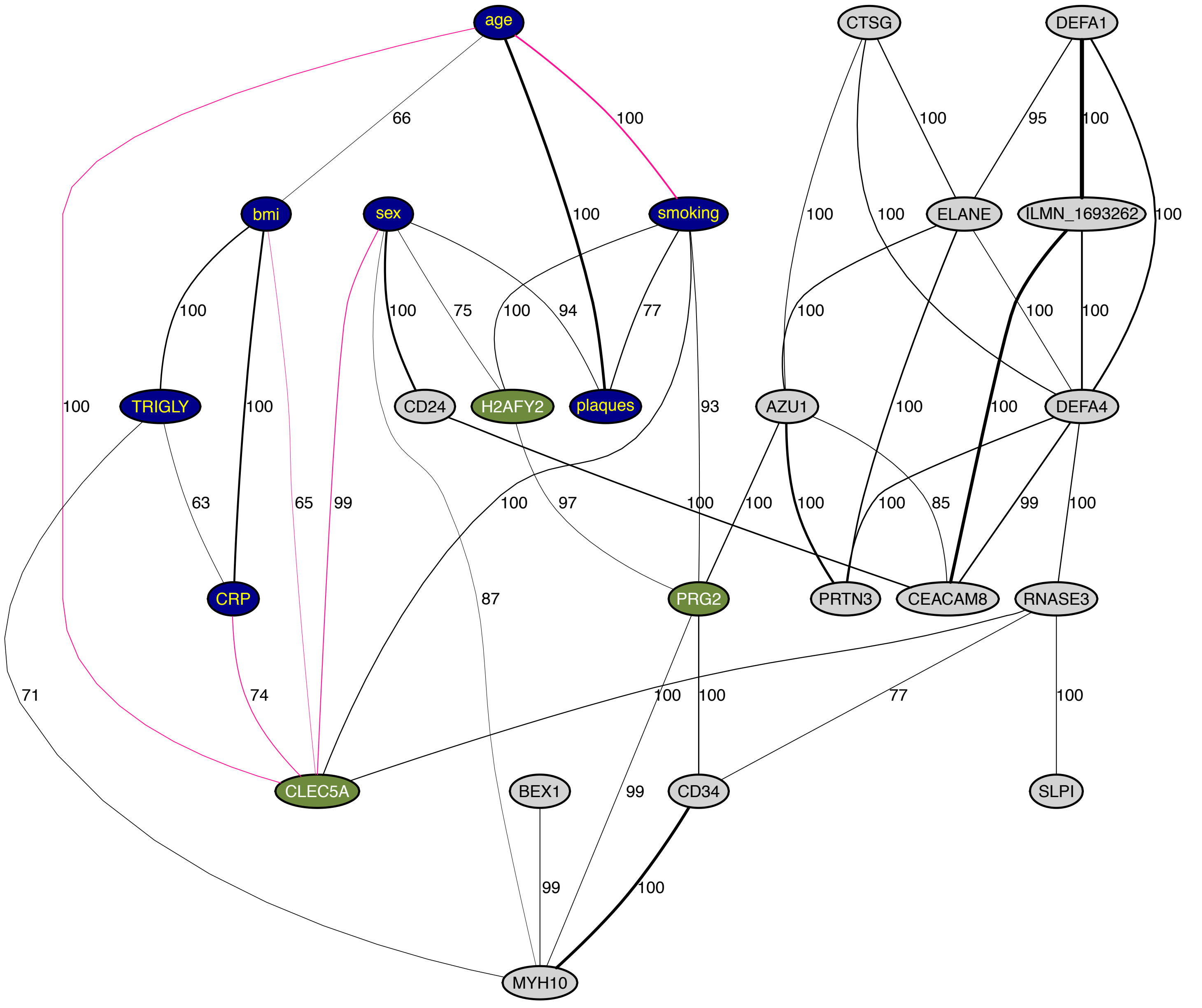

Network Skeleton for Pattern 58

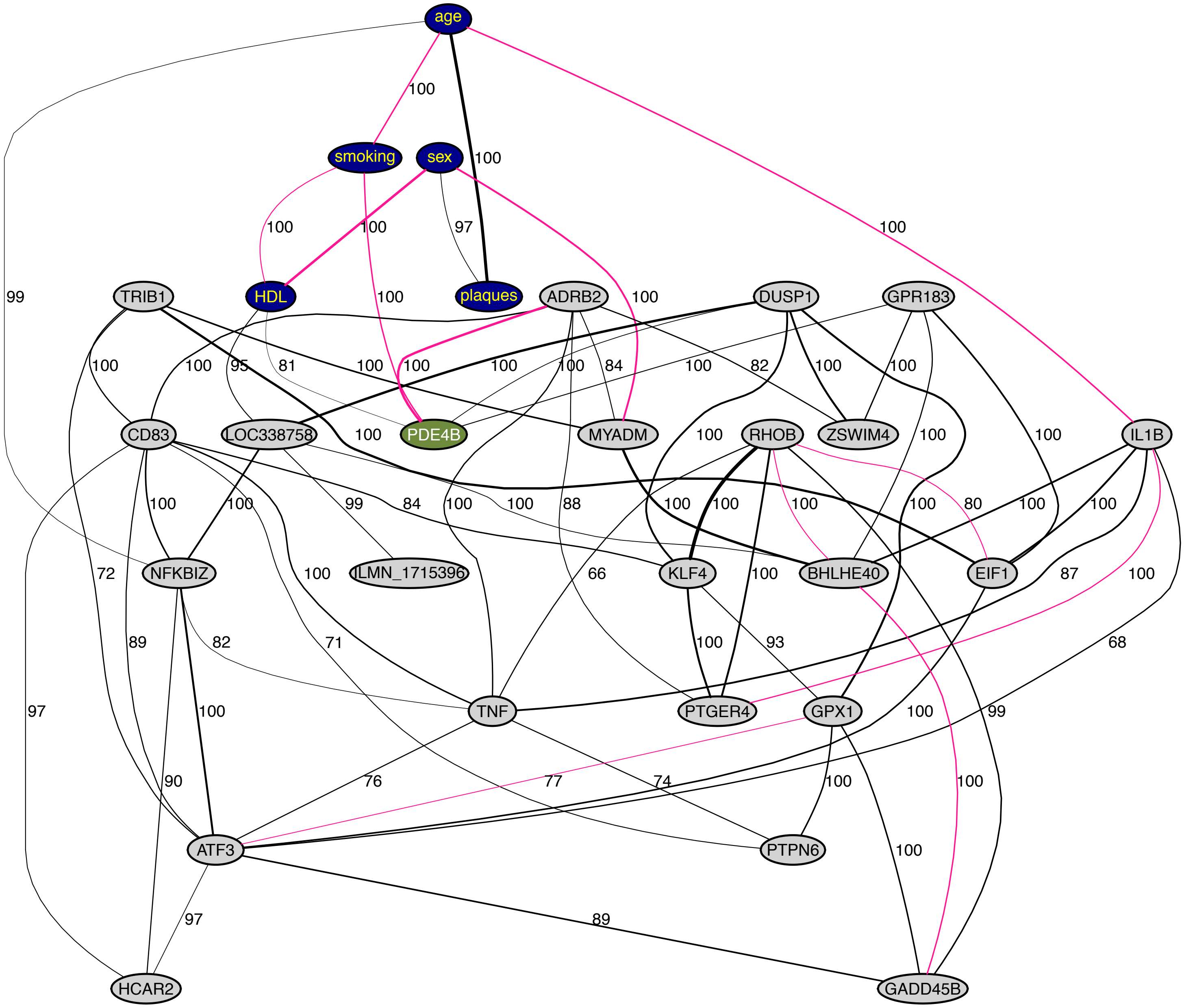

Supplement: Text S2 — Network plots for all ICA modules. (PDF) [file pone.0050888.s017.pdf]
